# Livestream Experiments: The Role of COVID-19, Agency, Presence, and Social Context in Facilitating Social Connectedness

**DOI:** 10.3389/fpsyg.2021.647929

**Published:** 2021-05-24

**Authors:** Kelsey E. Onderdijk, Dana Swarbrick, Bavo Van Kerrebroeck, Maximillian Mantei, Jonna K. Vuoskoski, Pieter-Jan Maes, Marc Leman

**Affiliations:** ^1^Department of Art, Music and Theatre Sciences, Institute for Psychoacoustics and Electronic Music, Ghent University, Ghent, Belgium; ^2^RITMO Centre for Interdisciplinary Studies in Rhythm, Time and Motion, University of Oslo, Oslo, Norway; ^3^Department of Musicology, University of Oslo, Oslo, Norway; ^4^Institute of Law and Economics, University of Hamburg, Hamburg, Germany; ^5^Department of Psychology, University of Oslo, Oslo, Norway

**Keywords:** livestream, concert COVID-19, social connectedness, agency, presence, parasocial interaction, virtual reality

## Abstract

Musical life became disrupted in 2020 due to the COVID-19 pandemic. Many musicians and venues turned to online alternatives, such as livestreaming. In this study, three livestreamed concerts were organized to examine separate, yet interconnected concepts—agency, presence, and social context—to ascertain which components of livestreamed concerts facilitate social connectedness. Hierarchical Bayesian modeling was conducted on 83 complete responses to examine the effects of the manipulations on feelings of social connectedness with the artist and the audience. Results showed that in concert 1, where half of the participants were allowed to vote for the final song to be played, this option did not result in the experience of more agency. Instead, if their preferred song was played (regardless of voting ability) participants experienced greater connectedness to the artist. In concert 2, participants who attended the concert with virtual reality headsets experienced greater feelings of physical presence, as well as greater feelings of connectedness with the artist, than those that viewed a normal YouTube livestream. In concert 3, attendance through Zoom led to greater experience of social presence, but predicted less connectedness with the artist, compared to a normal YouTube livestream. Crucially, a greater negative impact of COVID-19 (e.g., loneliness) predicted feelings of connectedness with the artist, possibly because participants fulfilled their social needs with this parasocial interaction. Examining data from all concerts suggested that physical presence was a predictor of connectedness with both the artist and the audience, while social presence only predicted connectedness with the audience. Correlational analyses revealed that reductions in loneliness and isolation were associated with feelings of shared agency, physical and social presence, and connectedness to the audience. Overall, the findings suggest that in order to reduce feelings of loneliness and increase connectedness, concert organizers and musicians could tune elements of their livestreams to facilitate feelings of physical and social presence.

## Introduction

March 2020 saw lockdown orders enforced on residents of Belgium in an effort to prevent COVID-19 from spreading among its population. From the beginning there were concerns about the consequences of a lockdown on issues such as people’s livelihood, their mental well-being, and the economic impact on specific sectors, such as hospitality and cultural industries. Consequently, this led to people searching for ways to endure. In the music industry it became a popular phenomenon for musicians to move their work online, resulting in a stark increase in livestreamed concerts (Quartz, 2020; Ren, 2020; Weaver et al., 2020; Vandenberg et al., 2021). Livestreaming is not a new phenomenon, but as usage increased, some argued that the pandemic could function as a ‘cultural catalyst for change’ toward a more virtually situated music industry (Lee et al., 2020), and that it enforced an already ongoing increase toward digital content consumption (Golden, 2019; Sim et al., 2020).

However, this assumption does not seem to be generalizable to all online music undertakings. Sim et al. (2020) found that music streaming (e.g., Spotify) services actually saw a decrease in usage during the pandemic. The acceleration of usage was seen in visual forms of entertainment, such as livestreaming and video streaming (Forbes, 2020). Possible reasons given for this disparity are shortened transit times, as a large group of people stream music during their commute (Sim et al., 2020), as well as consistent access to WiFi (Forbes, 2020), which exemplifies a preference for incorporating visuals in online music consumption when given the opportunity. However, an additional reason for people’s engagement with online livestreaming could be more socially oriented.

Indeed, previous research has demonstrated the importance of the social aspects of livestreaming. Hilvert-Bruce et al. (2018) found that viewer engagement on Twitch could be motivated by social interaction, sense of community, meeting new people, and lack of external support in real life. Hamilton et al. (2014) argued that livestreams on Twitch functioned as virtual third places where people create informal communities in which to socialize. Also, Friedländer (2017) revealed social motives to engage in livestreaming, such as socializing, the need to reach a specific group, and the need to communicate. Next to this, the fact that musicians moved to free and easily accessible platforms like YouTube, Twitch, Facebook, and Instagram (Quartz, 2020; Vandenberg et al., 2021), with no direct economic gratification for their performances, suggests that there is another underlying motive to engage their audiences in online livestreaming. With many artists emphasizing social connection, social resilience, and feelings of togetherness in their online performances (Vox, 2020) this seems to hint at social motivations as well.

Music making and listening serve a social function, with research showing music can facilitate social bonding, group cohesion, and feelings of togetherness (Nettl, 1983, 2000; McNeill, 1995; Freeman, 2000; Harland et al., 2000; Dunbar, 2004; Laiho, 2004; Olaveson, 2004; Bakagiannis and Tarrant, 2006; Bensimon, 2012; Demos et al., 2012; Boer and Abubakar, 2014; Volpe et al., 2016). However, the facilitation of these social feelings within musical livestream contexts needs further investigation. For example, Nguyen (2018) found that livestreaming of classical music concerts moved the experience out of rigid conventions of classical music culture into a virtual space where social interaction could exist more freely. As a result, livestreaming of a concert enhanced the sense of community among the audience. However, the context of the ‘live concert’ (i.e., the concert where performance and audience exist in the same physical space) seems to be of importance. During the current pandemic Vandenberg et al. (2021) investigated livestreamed rave parties. While highly popular, these were often accompanied with frustration of not experiencing the ‘real thing,’ during which audience members would actually dance and attune to others to feel and move as a unified entity. While the audience members were aware of each other (i.e., through means of online chat), they were inhibited to become fully immersed in the livestreamed experience. These studies encourage us to look closer at what variables are important in facilitating social connectedness in virtual concert environments.

Therefore, a main objective of the present exploratory study was to investigate variables that might facilitate and enhance feelings of social connectedness in livestreamed concerts. The importance of this question becomes apparent in light of the current pandemic, where mental health issues and social isolation have become critical concerns (Brooks et al., 2020). Social isolation can have detrimental health effects that are comparable to high blood pressure, obesity, or smoking (House et al., 1988), and previous studies on the imposition of quarantine during previous outbreaks show increased risk of suicide (Barbisch et al., 2015), depression (Hawryluck et al., 2004), stress (DiGiovanni et al., 2004), and post-traumatic stress symptoms (Reynolds et al., 2008). Music has the ability to relieve feelings of stress, anxiety, and loneliness (e.g., Thayer et al., 1994; McKinney et al., 1997; North et al., 2000; Saarikallio and Erkkilä, 2007; Särkämö et al., 2008; Lippman and Greenwood, 2012), regulate moods (e.g., North et al., 2000; Saarikallio and Erkkilä, 2007; Särkämö et al., 2008; Juslin and Sloboda, 2010; Thoma et al., 2012; Groarke and Hogan, 2016; van den Tol et al., 2016), act as a social surrogate (e.g., Schäfer and Eerola, 2020; Schäfer et al., 2020), and foster social bonding. Therefore, music may support people during times of crisis and social isolation.

During the period of De Gentse Feesten – one of Europe’s biggest music and theater city festivals in Ghent (Belgium) – we organized three livestreamed concerts, as it was expected that people would be looking for alternatives as a result of its physical cancelation due to the pandemic. The decision to livestream these concerts (as opposed to streaming pre-recorded videos) is rooted in previous research showing the experience to be more comparable to live concerts than pre-recorded videos (Shoda and Adachi, 2012; Jola and Grosbras, 2013; Kjus and Danielsen, 2014; Skjuve and Brandtzaeg, 2020). All three concerts focused on feelings of social connectedness, but each concert investigated an additional variable of interest.

The first concert focused on the concept of agency (i.e., subjective feeling of control). We aimed to give half of the participants a sense of agency by providing them with the option to vote for the last song to be played. Feelings of shared agency (i.e., feeling part of a larger whole; Pacherie, 2012) were also measured. While previous research has encouraged audience involvement to enhance the concert experience (Sloboda and Ford, 2019), here it was specifically investigated how feelings of agency affected feelings of social connectedness during a livestreamed concert. Additionally, the effect of whether the preferred song was played on feelings of connectedness was investigated, as previous research has shown success and failure influence feelings of agency (Miller and Ross, 1975; Phillips et al., 1998; Aarts, 2007; Obhi and Hall, 2011). We hypothesized that voters would feel more agency and more socially connected, as having a say would give a sense of control, and this sense of control would make their action feel as if they were contributing as part of a bigger whole.

The second concert focused on the concept of presence by dividing the audience into three groups, namely, a group watching a normal livestream, another watching a livestream with Youtube’s 360° option, and a group watching with a virtual reality (VR) headset. The ability to feel present inside a digital world (e.g., as if the computer interface disappears) has received increasing interest from researchers (Västfjäll, 2003; Sanchez-Vives and Slater, 2005; Riva et al., 2007; Takatalo et al., 2008; Slater et al., 2009; Glowinski et al., 2015; Çamcı and Hamilton, 2020). While a connection between presence and togetherness has been theorized before (Durlach and Slater, 2000), the aim of the current study was to investigate how feelings of presence contribute to feeling socially connected during a livestreamed concert. We hypothesized that as presence would increase over the groups, respectively, the amount of social connection would increase as well, as they would experience the digital environment as more of a shared space with the performers and other audience members.

Finally, in the third concert the emphasis was on social context. As the study by Nguyen (2018) showed, livestreamed concerts are capable of fostering social connectedness. However, in Vandenberg et al. (2021) participants were aware of each other, but only through means of a chat function. During this concert, comparisons were made between an audience that attended via a normal livestream, and an audience that attended via Zoom. In both cases participants were allowed to chat. We hypothesized that social connectedness would be highest for the audience attending in Zoom, as actually seeing others would make it easier to see the experience as shared.

Taking all three concerts together, our study provides an exploratory investigation into some initial variables to consider for enhancing social connectedness during livestreamed concerts. These are all considered in the context of the current pandemic and investigate the (short-term) effects of virtual concerts on feelings of loneliness, anxiety, isolation, lack of companionship, worrying about self and others, and forgetting of COVID-19. Furthermore, general musical behaviors during the pandemic [e.g., (livestream) concert attendance, music listening habits] are considered.

## Materials and Methods

### Participants

One hundred and twenty eight (66 women, 59 men, 3 preferred not to say) people registered to attend one or more concerts. Ages ranged from 19 to 76 years (*M* = 36.9, *SD* = 12.4). Participants came from 15 different countries. The majority resided in Belgium (*n* = 70) and the Netherlands (*n* = 32). The remaining participants resided in Italy (*n* = 8), France (*n* = 3), Canada (*n* = 2), Germany (*n* = 2), India (*n* = 2), Spain (*n* = 2), Argentina (*n* = 1), Chile (*n* = 1), the Czech Republic (*n* = 1), the Ivory Coast (*n* = 1), the United Kingdom (*n* = 1), the United States (*n* = 1), and Switzerland (*n* = 1). Fifty three people registered for the first concert, 68 for the second, and 55 for the last concert. The post-concert questionnaire was completed by a smaller portion, which resulted in a sample size of 29 participants in concert one, 32 participants in concert two, and 28 participants in concert three. An overview of the demographics of these participants can be found in Table 1. Two people were excluded for data analysis from the first concert, and one for both the second and third concert due to unadjusted hearing and vision problems.

**TABLE 1:** Amount of people who completed the post-concert questionnaire, number of participants per subgroup, and general demographics.

	Concert 1: Agency	Concert 2: Presence	Concert 3: Social Context
**Total *n***	**29**	**32**	**28**
*n* subgroup	16 voted 13 could not vote	12 YouTube 2D 9 YouTube 360° 11 VR headsets	6 live attendance 11 Zoom 11 YouTube (2D)

Age	Range: 25–62 years *M* = 37.7 *SD* = 11.2	Range: 19–76 years *M* = 36.5*SD* = 15.8	Range: 26–62 years *M* = 37.0*SD* = 11.1

Gender	15 women 12 men 2 preferred not to say	18 women 13 men 1 preferred not to say	17 women 11 men

### Procedure

**FIGURE 1:**
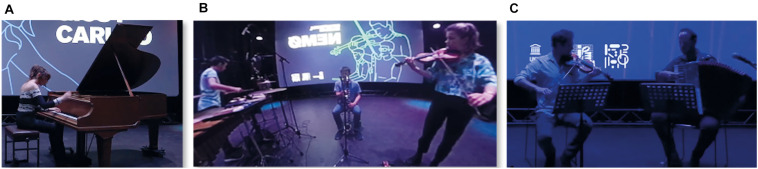
Snapshots of the three livestreamed concerts in order of occurrence. **(A)** Concert 1: Agency, **(B)** Concert 2: Presence, **(C)** Concert 3: Social Context. The snapshots of the first **(A)** and third **(C)** concerts provide a standard perspective, while the second snapshot comes from the livestream with 360 view **(B)**.

All procedures were approved by the Ethics Commission of the Faculty of Arts and Philosophy of Ghent University. Three concerts were livestreamed from the Art and Science Interaction Lab (ASIL) of Ghent University during the Gentse Feesten festival that occurs annually during 1 week in July (see Figure 1). In 2020, the festival was conducted under the social distancing constraints caused by the COVID-19 pandemic. The first concert took place on the 21st of July and included a pianist performing five classical pieces for solo piano. The second concert took place on the 22nd of July, where three musicians performed a contemporary experimental improvisation on bass clarinet, violin, and percussion. Lastly, on the 27th of July, the third concert included two musicians playing five pieces of a mix of Arabic and Western music on violin and accordion (see Supplementary Material 1 for titles of stimuli). All concerts lasted approximately 30 min, including an introduction, explanations, and/or concluding remarks by a presenter (see Supplementary Material 2 in the Supplementary Material). Participants pre-registered for each concert by filling out a pre-experimental questionnaire (see section “Questionnaire Design”), and were assigned a personal ID code to maintain anonymity for the duration of the experiment. After registration participants were randomly assigned to different audience groups for their chosen concert(s). Links to the concerts were sent via email. Participants were encouraged to watch the concert with a laptop (with the exception of the VR group) while using headphones in order to reduce variability among the sample. At the end of each concert participants immediately completed a questionnaire (using their personal ID code) on their experience of the concert, which they received via email as well.

#### Concert 1: Agency

To manipulate agency, one group of participants voted to select the final piece of the performance while the other group was not given the option to vote. After the concert was finished, participants who did not vote reported which piece they would have chosen. This allowed us to measure whether participants’ preference was played regardless of whether they voted or not. Studies have shown people can feel agency over a successful task (here: preferred song played) even when they had little influence on the outcome (Tennen and Sharp, 1983; Aarts et al., 2005). Participants who could not vote were aware that other members of the audience were voting to select the final piece. Participants in the voting condition chose between two pieces (i.e., it was stated they could vote between a rhythmic and a melodic piece). Voting was done via a Microsoft Forms link they received in their invitation email. The rhythmic piece received the most votes and thus was played by the pianist (for full concert, see: https://youtu.be/Ou191NRHBDs).

#### Concert 2: Presence

Feelings of presence were manipulated with three different viewing conditions. One group watched the concert as a regular YouTube livestream^1^. Another group watched the livestream with a 360° view^2^ on a standard screen (e.g., laptop screen, desktop monitor). To create this condition a 360° camera (Ricoh Theta S 360) was positioned in the middle and in front of the performers. In this condition, participants could control their viewing direction using their mouse. The third group watched the concert in virtual reality using the 360° stream. Specifically, participants were provided with virtual reality glasses which were hard plastic smartphone holders, and participants were provided with instructions on how to adjust lens-width and image focus (see Supplementary Material 2.2.3). In this condition participants streamed the concert to their phone and could control their viewing direction by moving their heads to “look around” at the performers, creating an immersive experience.

#### Concert 3: Social Context

During the final concert, one group physically attended the live performance. These participants wore face masks, disinfected their hands upon entry, and kept a safe distance (i.e., at least 1.5 m apart) from other audience members in accordance with social distancing restrictions that were present at the time. A second group watched the concert via a standard YouTube livestream, but for this particular concert, the chat function was enabled. A third group watched via Zoom, where the chat function was enabled as well, and participants were asked to keep their webcams on. The video livestream presented to both the group watching via YouTube and Zoom is available here: https://youtu.be/RZbyA6a_rPk.

Additionally, during this concert all participants were voluntarily asked to install and use a smartphone application developed by RITMO to track their movement using gyroscope, accelerometer, and location data (the MusicLab App^3^). Instructions on how to install, calibrate and use the app were given to participants via email beforehand (see Supplementary Material 2.3.4), and again during the introduction of the concert. Participants were asked to position their smartphone on their chest while the app recorded their motion. After the concert, participants stopped the app and data was sent anonymously to a secure database for analysis (along with their personal ID code). Movement data of the two musicians was recorded via this app as well.

### Technical Specifications

Materials used for each concert are listed in Table 2. Audio was recorded using several microphones and routed over a Dante network using Focusrite Rednet interfaces to Ableton Live 10 (version 10.1.15). Audio and video were streamed to YouTube and Zoom using the Open Broadcaster Software (Version 25.0.8). A visual animation was projected on a large screen behind the musicians using a 4K projector to enhance the concert atmosphere. These could be seen by all participants and consisted of silhouette portraits of the artists next to their names and to the words “Experimental Sessions,” which was the working title for the concert series during recruitment. During concert one, the visuals were static. However, during the second and third concert small colored lights moved through the silhouettes sporadically.

**TABLE 2:** Technical setups per concert.

	Concert 1: Agency	Concert 2: Presence	Concert 3: Social Context
**Audio**	2x Rode NT1-A for piano 2x Shure SM58 LC for speaking	2x Oktava MK-012 MSP2, and 1x Devine SM100 for percussion 1x DPA d:vote 4099 for violin 1x Shure SM57 for bass clarinet 1x Shure SM58 LC for speaking	3x Rode NT1-A for music 3x Shure SM58 LC for speaking

**Video**	1x Sony PXW-X70 camera 1x Ephiphan HD video grabber	1x Logitech conference camera 1x Ricoh Theta S 360 camera 2 instances of OBS 11x Virtual reality glasses for smartphone	1x Sony PXW-X70 camera 1x Epiphan HD video grabber

*Concert 1 was streamed to YouTube (video 1080 p). Concert 2 had two different YouTube streams: a 2D view (720 p), and one with 360° view (720 p). A group of participants received VR glasses with which they could watch the concert in 3D with the 360° view livestream. Concert 3 had three different audience groups: one group attended live, one group watched the concert via a YouTube stream (1080 p), and one group of participants attended via Zoom (1080 p). For the latter two, chat functions were enabled.*

### Questionnaire Design

Upon registration every participant filled out the same questionnaire (see Supplementary Materials 3 and 4 in the Supplementary Material for all English and Dutch questionnaires, respectively). This consisted of informed consent, questions on general demographics, music listening habits, concert behavior before and during the pandemic, emotional state during the pandemic, the Empathic Concern scale from the Interpersonal Reactivity Index (Davis, 1980), and questions related to organizational aspects (e.g., which concerts they aimed to attend, interest in physically attending the third concert, experience with Zoom).

After each concert, participants were asked to fill out a questionnaire on their experience. These questionnaires were highly similar across concerts, but varied slightly according to the concerts’ particular focus. All questionnaires contained questions on: the audio and video technology that was used and its perceived quality, familiarity with the artist and others in the (online) audience, general experience (e.g., how long they watched, concentration level, whether they watched alone or with others), feelings of social connection with the artist(s) and audience [which was asked explicitly and with the Inclusion of Other in the Self scale (IOS; Aron et al., 1992)], experience of presence using the multimodal presence scale (MPS; Makransky et al., 2017), and feelings of agency they felt independently (self-agency) and shared with other participants (shared agency). Feelings of agency were gathered by first giving a definition of the concepts. The questions read: “Feelings of agency can be described as having a subjective feeling of control. In other words, agency is feeling a general sense of control over what you are doing or over the situation you are in. Given this definition, to what extent did you feel you had agency over your concert experience (on a scale from 1 Not at all – 5 Very much)?”, and “When two or more people partake in something together, they can feel a sense of shared agency over what they are doing or the situation they are in. When you feel this shared agency, you feel as if you are doing something together (your experience is the product of something done together). When you do not feel shared agency, you feel as if others did not have any control over your experience, and that you acted completely independently. Given this definition, to what extent did you feel shared agency over your concert experience (on a scale from 1 No shared control – 5 Complete shared control)?”.

Furthermore, participants were asked whether the concert temporarily influenced their emotional states (e.g., change in feelings of loneliness, anxiety, forgetting worries of COVID-19). Here, forgetting worries of COVID-19 was questioned through a 1 (Do not agree at all) – 5 (Agree completely) scale, based on the statement “The concert (temporarily) made me forget my worries surrounding COVID-19.” Further inquiry on emotional states was assessed through the question “To what extent did you feel the following more or less after the concert?” on a 5-point scale (i.e., a lot less, less, unchanged, more, a lot more) regarding loneliness, lack of companionship, isolation from others, and anxiety. Finally, an opportunity was given to leave comments or remarks.

Additionally, the first concert included questions on familiarity with the pieces participants could choose from and the influence of being able or unable to vote on concert experience. The second concert was extended with a question on previous experience using VR (for participants watching with VR), and the Igroup Presence Questionnaire (IPQ; Schubert et al., 2001). Lastly, the third concert’s questionnaire contained an additional question on whether participants used the chat function during the concert.

## Results

Analyses were performed using R (version 4.0.3). Manipulation checks were conducted using robust non-parametric tests to evaluate if the experimental conditions effectively manipulated participants’ feelings of agency and presence. Bayesian ordinal regression was used to assess the main question of the study (i.e., determine what variables explained feelings of social connectedness to the artist and the audience). Finally, correlational analysis was conducted to better understand the impact of COVID-19 on concert behaviors and experiences.

However, some considerations are in order. First, the experience of presence was collected at each concert using the multimodal presence scale (MPS; Makransky et al., 2017). However, the questions were re-worded to match the virtual concert context. Rephrasing the questions resulted in a slightly different subscale structure as measured by a principal component analysis, such that one item that initially loaded onto the social presence subscale loaded on the physical presence subscale (Swarbrick et al., accepted; see Supplementary Material 5). Furthermore, data of the physically attending group in Concert 3, and the motion data collected through the MusicLab App during this concert, are not included in inferential analyses due to low sample sizes. Descriptive statistics on the physically attending group (*n* = 6) can be found in the Supplementary Material 6. Motion data (*n* = 4) is available upon request to author DS. Moreover, all experimental groups had relatively low sample sizes (max. *n* = 16; min. *n* = 9; refer to Table 1). Therefore, all results should be interpreted with caution and future research should aim to replicate the findings presented here.

### Manipulation Checks

**FIGURE 2:**
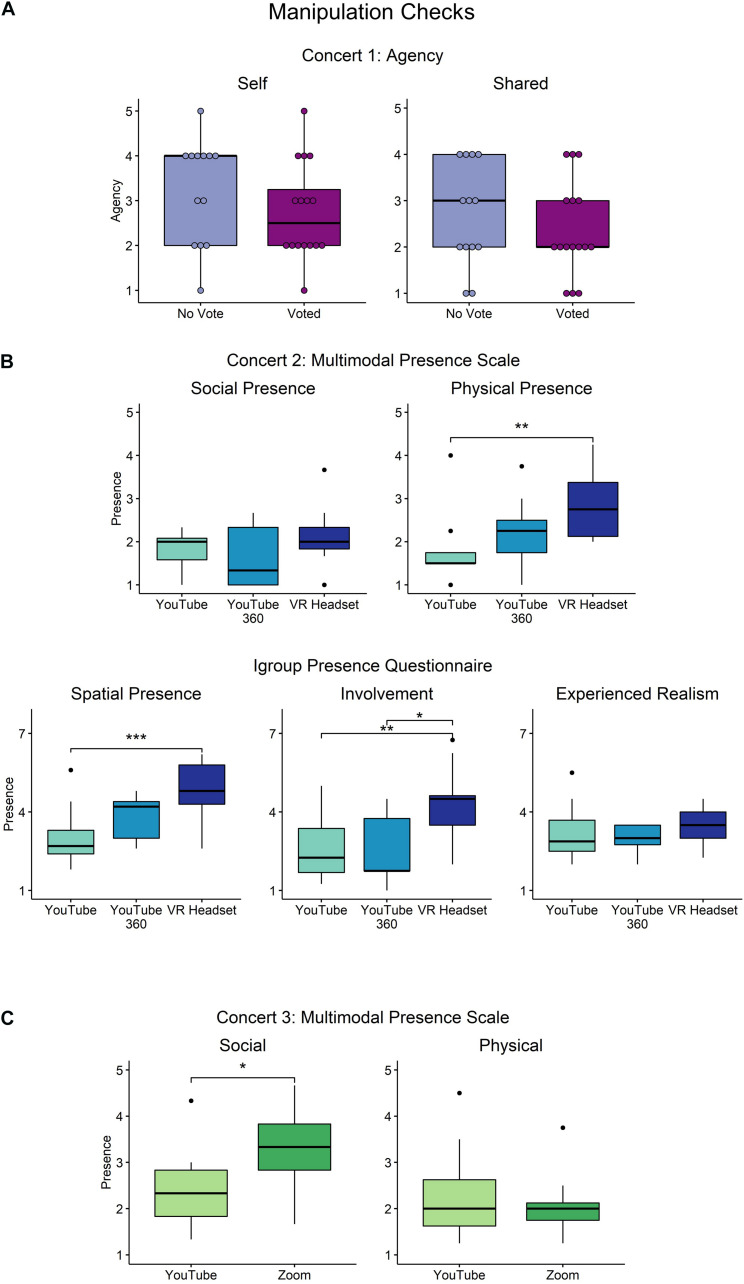
**(A)** Concert 1: The manipulation of voting was not effective in producing differences in self- or shared agency. **(B)** Concert 2: The manipulation of using a VR headset was effective in increasing physical presence (MPS), spatial presence (IPQ), and involvement (IPQ) relative to a regular YouTube livestream. **(C)** Concert 3: The manipulation of social context by viewing in Zoom was successful in increasing social presence. The BH method was used for adjusting *p*-values. ***p < 0.001, **p < 0.01, *p < 0.05.

To robustly evaluate the manipulations, we tested for differences in agency (concert 1) and presence (concerts 2 and 3) between experimental groups using non-parametric methods. An alpha level of *p* = 0.05 was used (see Figure 2).

#### Concert 1: Agency

A Kendall rank correlation test was conducted to assess whether the ability to vote (categorical predictor) increased participants’ self or shared agency (ordered variables). Providing participants with the ability to vote did not increase participants’ sense of self-agency (*r*_τ_ = –0.21, *p* = 0.23) or participants’ sense of shared agency (*r*_τ_ = –0.14, *p* = 0.42). Another Kendall rank correlation test was performed to compare participants whose preferred song was performed and participants whose preferred song was not performed (regardless of voting ability), as participants who preferred the voted option might feel a false sense of agency. Results showed no influence on their sense of self-agency (*r*_τ_ = –0.02, *p* = 0.93) or sense of shared agency (*r*_τ_ = –0.15, *p* = 0.38) (see Figure 2A).

#### Concert 2: Presence

Two measures of presence were collected: the Multimodal Presence Scale (MPS) and the IPQ (Schubert et al., 2001; Makransky et al., 2017). The effectiveness of the manipulation was assessed by comparing the average presence scales and their respective subscales across the viewing conditions of the normal YouTube stream, the YouTube 360° stream, and the VR headset. Kruskal–Wallis rank sum tests were used to assess differences between the three groups on the measures of presence. Pairwise comparisons were performed with the Wilcoxon rank sum test and *p*-values were adjusted using the BH false discovery rate method (Benjamini and Hochberg, 1995; see Figure 2B). Effect size estimates are provided in Supplementary Material 7.

##### Multimodal presence scale

A significant difference was found in the average MPS between the three groups [*H*(2) = 9.21, *p* = 0.01]. The group that watched with the VR headset experienced more presence than the group that watched the normal YouTube livestream (*p* = 0.013). There were no differences between the 360° view and the other conditions (regular vs. 360°: *p* = 0.19, 360° vs. VR headset: *p* = 0.12). The MPS has two subscales: social presence and physical presence. No differences were found between groups in the subscale of social presence [*H*(2) = 1.66, *p* = 0.43]. In the subscale of physical presence, there was a significant difference between groups [*H*(2) = 10.53, *p* = 0.005]. Participants who watched the concert with the VR headset reported experiencing more physical presence than those watching the regular YouTube livestream (*p* = 0.004). There were no significant differences between the participants watching the 360° stream and the other groups (regular vs. 360°: *p* = 0.12, 360° vs. VR headset: *p* = 0.27).

##### Igroup presence questionnaire

There was a significant difference between the groups’ average IPQ scores [*H*(2) = 12.99, *p* = 0.002]. The VR headset group experienced greater presence than the regular YouTube group (*p* = 0.006) and the 360° group (*p* = 0.007). There was no difference between the groups watching the normal YouTube stream and the 360° view (*p* = 0.21). The IPQ has three subscales: spatial presence, involvement, and experienced realism. Examining the subscale of spatial presence revealed a significant difference between groups [*H*(2) = 12.41, *p* = 0.002] such that the VR headset group experienced more spatial presence than the regular YouTube group (*p* = 0.005), and there was a trend toward the VR headset group experiencing more spatial presence than the 360° group (*p* = 0.054). There was also a trend suggesting that participants who watched the 360° concert may have experienced a greater sense of spatial presence than the regular YouTube stream (*p* = 0.054). Additionally, significant differences between groups were found in the involvement subscale [*H*(2) = 9.20, *p* = 0.01]. The VR headset group experienced more involvement than the regular YouTube group (*p* = 0.018) and the 360° view group (*p* = 0.029). There was no difference between the regular YouTube group and the 360° group. Lastly, there were no significant differences between groups on the experienced realism subscale [*H*(2) = 2.77, *p* = 0.25].

#### Concert 3: Social Context

The MPS was used as the measure of presence in concert 3. The effect of the social context manipulation between a regular YouTube stream and viewing in Zoom was assessed using the Wilcoxon rank sum test (see Figure 2C).

##### Multimodal presence scale

There was no significant difference between groups in the average MPS (*W* = 80, *p* = 0.21). The subscale of social presence revealed a significant difference between groups (*W* = 97, *p* = 0.017) such that the group that watched the concert on Zoom experienced more social presence than the group that watched on YouTube. There was no significant effect of group on the subscale of physical presence (*W* = 57, *p* = 0.84).

### Bayesian Regression

To address the main hypotheses that increased feelings of agency and presence would lead to greater social connection toward the artist and audience, we conducted hierarchical (multi-level) Bayesian ordinal regression. Modeling was conducted using Stan, a high performance statistical computing platform (Stan Development Team, 2019), which interfaced with R using the cmdstanr package [Copyright (c) 2019, Stan Development Team, 2019]. Given prior and likelihood distributions, Stan conducts adaptive Hamiltonian Monte Carlo, a type of Markov chain Monte Carlo estimation method, to generate samples from the posterior distribution of all model parameters.

A cumulative model was employed with logit link to estimate the unobservable, continuous latent variable of social connection that underlies the ordinal data collected with the responses to the measures of social connection with the artist and audience (“To what extent did you feel connected to the artist/audience?” 1 Not at all – 5 Very much) and the Inclusion of Other in the Self scale (“Looking at the figure below, which of these circles describes you and the artist best during the concert experience?”) 1 (visual presentation of completely non-overlapping but touching circles) – 7 (visual presentation of nearly completely overlapping circles; Aron et al., 1992). Bayesian ordinal regression involves estimating both the regression coefficients and the thresholds that separate each response option along the ordinal scale. For the purposes of this study, interest only lies in the effect of each predictor, therefore information on threshold estimation is included in the Open Science Foundation repository of this study^4^. A clear description of cumulative models and Bayesian ordinal regression can be found in a tutorial for psychologists (Bürkner and Vuorre, 2019).

Separate models were fit for each main predictor of interest for each of the three concerts (see the OSF repository for model specification in Stan). Each model used a set of variables to predict both social connection to the artist and the audience with both connection measures [recall that social connection to the artist and audience were measured in two ways: (1) by explicitly asking how connected they felt and (2) using the IOS scale (Aron et al., 1992)]. The main predictors of interest were the experimental manipulations of being allowed to vote (concert 1), viewing technology of VR headset and YouTube 360 as compared to regular YouTube (concert 2), social context of Zoom as compared to regular YouTube (concert 3), and the variables of self-agency, shared agency, and physical and social presence. Models were also fit on the overall data to investigate the effect of the variables that were collected at every concert on social connection. All models were specified so that the thresholds were able to vary by the same amount across participants (Gelman and Hill, 2006; Bürkner and Vuorre, 2019). Specifically, specifying the models in this way accounted for the variability between participants by allowing the thresholds between response options to slide along the latent variable scale for each participant, while holding the space between thresholds the same across participants.

Every model also contained predictors other than the main predictors of interest to control for confounds and examine the influence of individual characteristics. Simple models included only predictors that were assessed prior to the experimental manipulation and these included individual characteristics of age (continuous), gender (categorical), empathic concern (continuous), the negative emotional impact of COVID-19 (loneliness, lack of companionship, isolation, anxiety, worry for themselves, and worry for others; the average of these ordinal variables was treated as continuous). The variables making up the measure of impact of COVID-19 showed acceptable reliability (Cronbach’s α = 0.74). Prior fan status (ordinal) may lead to more social connection toward the artist, and being with others or knowing others in the audience personally could result in more social connection toward the audience, therefore these predictors were used to predict only connection to the artist and audience, respectively. Whether participants had prior experience using a VR headset was included as a predictor in the models for concert 2 (categorical).

To account for uncontrolled elements of concerts, more complex models that included variables that were measured after the experimental manipulation were included to control for and assess their impact on social connection. Specifically, confounds included whether participants experienced any connectivity issues (categorical), the amount of time they watched the concert (continuous), participants’ concentration levels (ordinal), and their reports of perceived audio and video quality (ordinal). Similarly, connection to the audience could be influenced by confounds of whether participants were attending the concert alone or with other people in the same physical space (numerical), and if they knew anyone else in the audience personally (categorical), therefore these variables were modeled to investigate and control for their influence on connection to the audience. The predictors measured after the experimental manipulations are not necessarily causally linked to feelings of social connection and thus the effects of these predictors should be interpreted cautiously. Refer to the OSF repository for the files specifying the models and for summary information on each predictor.

Bayesian modeling requires the specification of priors. Prior specification is still an open and active area of research. Here, generic weakly informative priors were set (Stan Development Team, 2020). The prior for the coefficients was specified as a normal distribution with mean of 0 and standard deviation of 1. The prior for the thresholds was specified such that the proportion of answers would be equal across all response categories. This was performed with an induced Dirichlet based on the number of response options of the outcome variable which pooled the thresholds toward equal spacing with some flexibility (Betancourt, 2019). The prior for the participants’ random effects was multivariate normal (multivariate because there were two outcome measures: connection to the audience and the artist), and the prior for the spread of participants’ random effects (and the concert random effects in the overall models) were specified as a half-normal distribution with a mean of 0 and standard deviation of 1, which restricts the spread to being a positive number and shrinks estimates toward 0. The prior for the correlation between the outcome variables measuring connection to artists and audience was specified with a Cholesky LKJ correlation distribution with η = 4, which favors the model toward smaller correlations (Lewandowski et al., 2009).

Interpretation of the effects occurs on the latent metric scale—that is the underlying continuous measure of social connection that the ordinal response scales have aimed to capture. To improve interpretation of the effects from the figures, age and time watching the concert were scaled to make the variance similar to other variables. Specifically, age was scaled by dividing by 10, and time watched was scaled by dividing by 5. There were three participants who preferred to not report their gender (i.e., two in concert 1, one in concert 2). To avoid creating unstable coefficients due to the sparse data on this third gender category, only the effect of being a woman was modeled such that the comparison group included men and these three participants. When a predictor’s 90% credible interval (CI) does not intersect with and is above (below) zero, then there is at least a 90% probability that the effect of this predictor on the outcome of social connection is positive (negative). On each end of the credible interval there is 5% probability that is not visible, therefore when the 90% credible interval is marginally crossing 0, it means that there is still nearly 95% probability that the effect of the predictor is positive or negative. Using 90% CIs is reasonable as credible intervals are typically wider than confidence intervals because Bayesian analyses account for uncertainty better than frequentist methods (Kruschke, 2014).

Models were assessed using model diagnostics which included checking the sampling transitions’ treedepth, lack of divergent transitions, Hamiltonian Monte Carlo potential energy, effective sample sizes, and split Rhat values. Posterior predictive checks were conducted to ensure that the predicted values approximated values present in the data. Predictive performance of the models was compared using expected log predictive density (elpd) assessed from a leave one out cross validation (LOO-CV) calculated by removing one participant from the model at a time.

#### Concert 1

**FIGURE 3:**
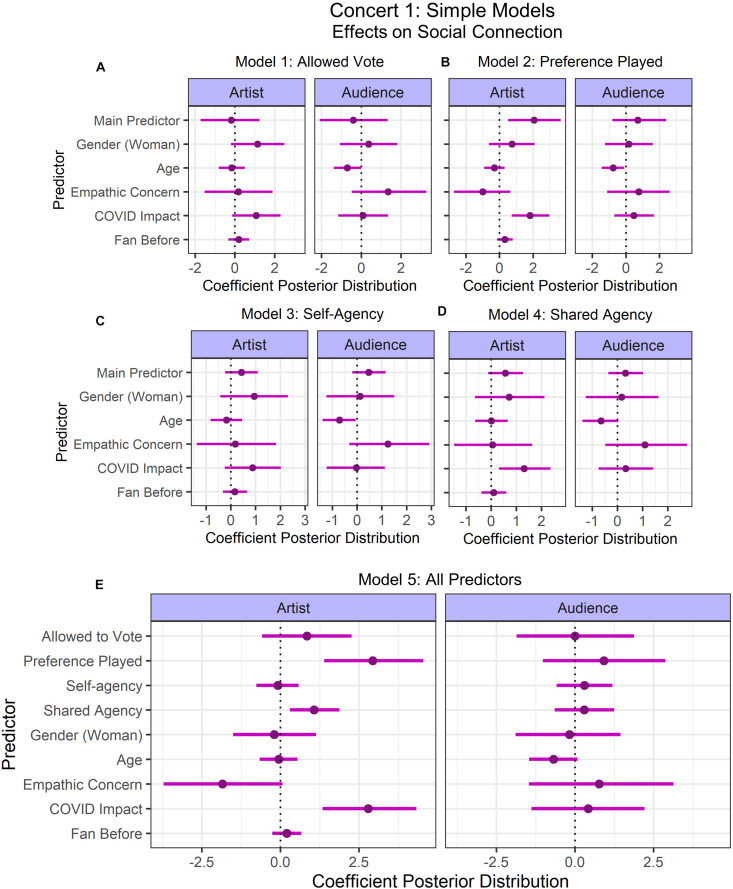
Concert 1’s simple models’ predictor coefficient estimates (points) and 90% credible intervals (lines) for the effects on social connection to the artist (left) and audience (right). Five models were fit to examine the individual contributions of **(A)** Allowed to Vote, **(B)** Preference Played, **(C)** Self-agency, **(D)** Shared Agency, and one model was fit to examine all predictors **(E)**. Lines indicate the 90% credible intervals (i.e., there is a 90% posterior probability that the coefficient of the predictor lies in that range). Credible intervals not intersecting 0 indicate that there is at least a 90% probability that the effect of that predictor on social connection is positive or negative.

**FIGURE 4:**
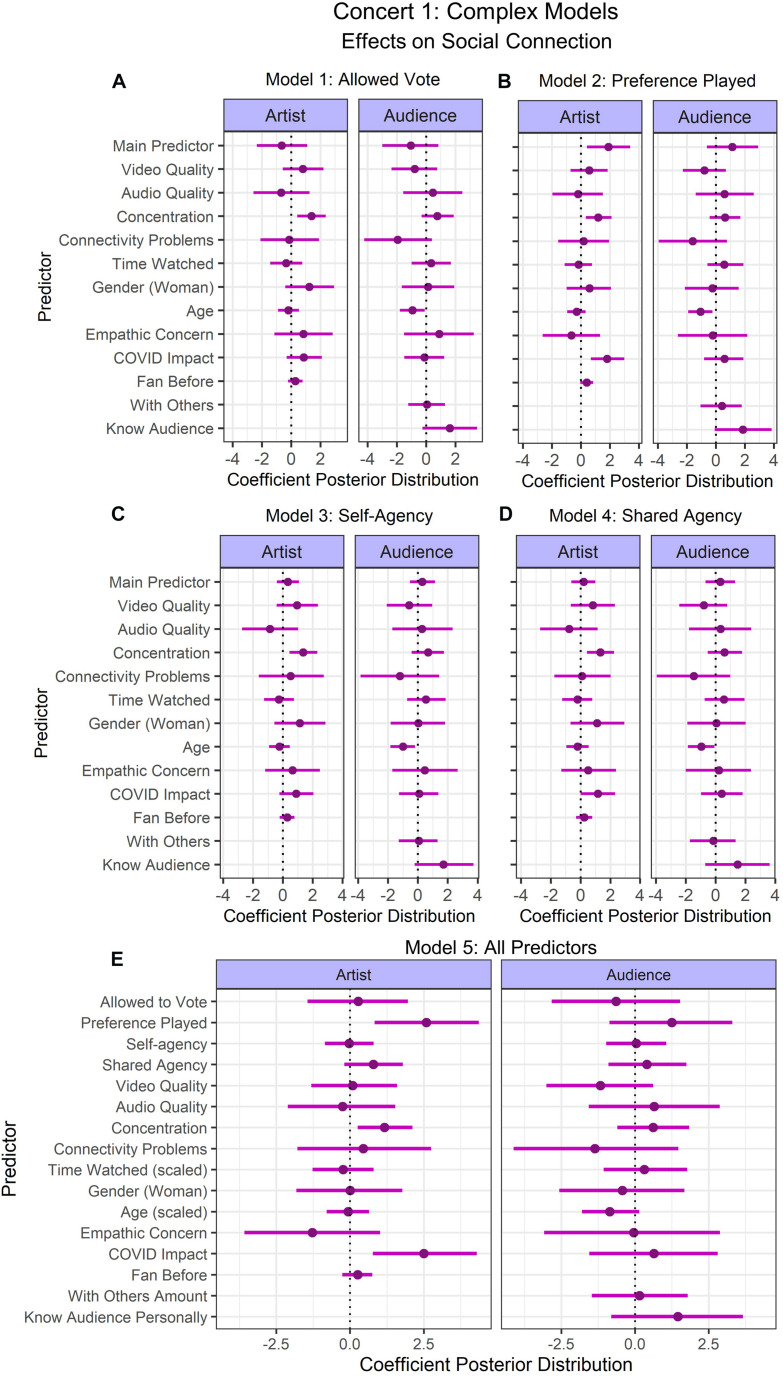
Concert 1’s complex models’ predictor coefficient estimates (points) and 90% credible intervals (lines) for the effects on social connection to the artist (left) and audience (right). Five models were fit to examine the individual contributions of **(A)** Allowed to Vote, **(B)** Preference Played, **(C)** Self-agency, **(D)** Shared Agency, and one model was fit to examine all predictors **(E)**.

Two participants did not respond to the IOS scales for connection to the artist and audience, therefore these values were imputed during model fitting. Five different models were fit for each of the simple and complex predictor selections (see Figures 3, 4), varying in the predictor of interest, such that there were two models each for the predictors of (1) whether participants were able to vote for their preferred song (Model 1: Allowed Vote; Figures 3, 4A), (2) whether their preferred song was played (Model 2: Preference Played; Figures 3, 4B), and their (3) perceived self-agency (Model 3: Self-Agency; Figures 3, 4C) and (4) shared agency (Model 4: Shared Agency; Figures 3, 4D). These separate models allowed us to examine the individual influence of each of these predictors. The fifth model included all of these predictors to examine which predictors are important when accounting for the effect of all predictors of interest (Model 5: All Predictors; Figures 3, 4E).

For the simple models, model comparisons revealed that there were no substantial differences in levels of fit (see Table 3). Results suggested that whether participants had their preferred song played positively predicted social connection to the artist with at least 90% probability in Model 2: Preference Played and Model 5: All Predictors (90%-CI: 1.39–4.54). However, this result should be interpreted carefully because participants who were not given the option to vote reported the song they would have voted for after the concert. In Model 4: Shared Agency, shared agency’s credible intervals intersected with 0 (90%-CI: –0.12–1.27). However, in model 5 with all predictors, shared agency positively predicted connection to the artist (90%-CI: 0.30–1.88). The impact of the coronavirus positively predicted connection to the artist in Model 2: Preference Played (90%-CI: 0.72–2.97), Model 4: Shared Agency (90%-CI: 0.31–2.36), and Model 5: All Predictors (90%-CI: 1.34–4.33). Age negatively predicted connection to the audience in Model 1: Allowed Vote (90%-CI: –1.38 to –0.01), Model 2: Preference Played (90%-CI: –1.44 to –0.09), and Model 3: Self-Agency (90%-CI: –1.39 to –0.05).

**TABLE 3:** Concert 1 models and their predictive performance.

Model fit	Elpd difference	SE difference
**Concert 1**		
**Simple Models**		
Model 2: Preference played	0	0
Model 4: Shared agency	–1.7	4.3
Model 3: Self-agency	–1.7	4.3
Model 5: All	–3.0	3.1
Model 1: Vote	–3.0	3.5
**Complex Models**		
Model 2: Preference played	0	0
Model 3: Self-agency	–5.0	3.4
Model 1: Vote	–5.3	3.6
Model 4: Shared agency	–5.5	3.2
Model 5: All	–9.0	3.2
**Concert 2**		
**Simple Models**		
Model 2: Presence	0	0
Model 1: Group	–4.1	3.5
Model 3: Group + presence	–4.3	3.0
**Complex Models**		
Model 1: Group	0	0
Model 2: Presence	–0.9	4.8
Model 3: Group + presence	–7.3	5.2
**Concert 3**		
**Simple Models**		
Model 2: Group + presence	0	0
Model 1: group	–14.7	3.7
**Complex Models**		
Model 2: Group + presence	0	0
Model 1: Group	–6.8	3.9

*A meaningful difference is 2x the standard error.*

For the complex models, the best model was Model 2: Preference Played. This model had an elpd value 3x greater than the corresponding standard error of the model including all predictors, possibly because Model 5 with all predictors was overfitting the data. In the complex models, despite Model 2 having better fit than Model 5, both models were very similar because almost all of the same predictors’ credible intervals did not intersect with 0. The only exception was that age negatively predicted connection to the audience with a credible interval that did not intersect with 0 in Model 2 (90%-CI: –1.90 to –0.22), while it intersected with 0 in Model 5 (90%-CI: –1.80 to 0.14). For simplicity, only the credible intervals from Model 5 will be reported, however, credible intervals for Model 2 are also available at the OSF repository (see footnote 4). Preference played (90%-CI: 0.84–4.37), concentration (90%-CI: 0.26–2.11), and COVID impact (90%-CI: 0.77–4.30) had credible intervals that did not intersect with 0, thus we can conclude with at least 90% posterior probability that these variables positively predicted social connection with the artist. All other predictors of social connection with the artist and audience intersected with 0.

#### Concert 2

**FIGURE 5:**
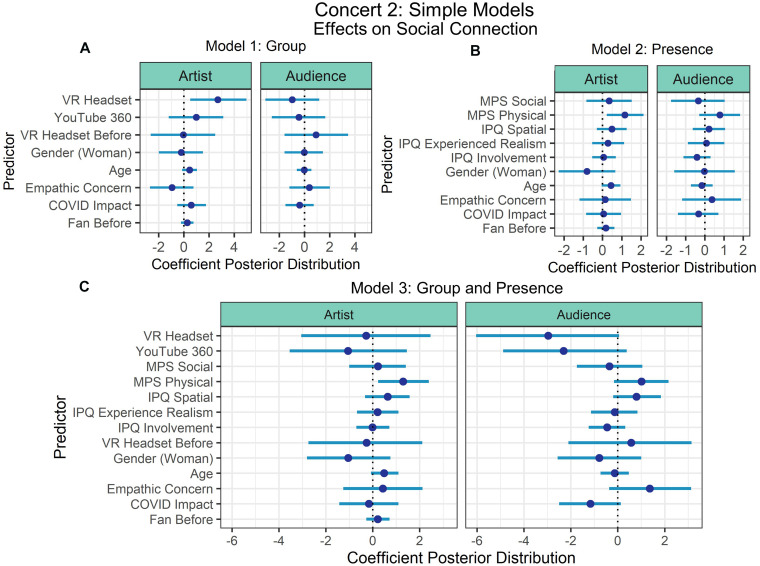
Concert 2’s simple models’ predictor coefficient estimates (points) and 90% credible intervals (lines) for the effects on social connection to the artist (left) and audience (right). Three models were fit to examine the individual effects of **(A)** Group and **(B)** Presence, and the third model examined the combined effects of Group and Presence **(C)**.

**FIGURE 6:**
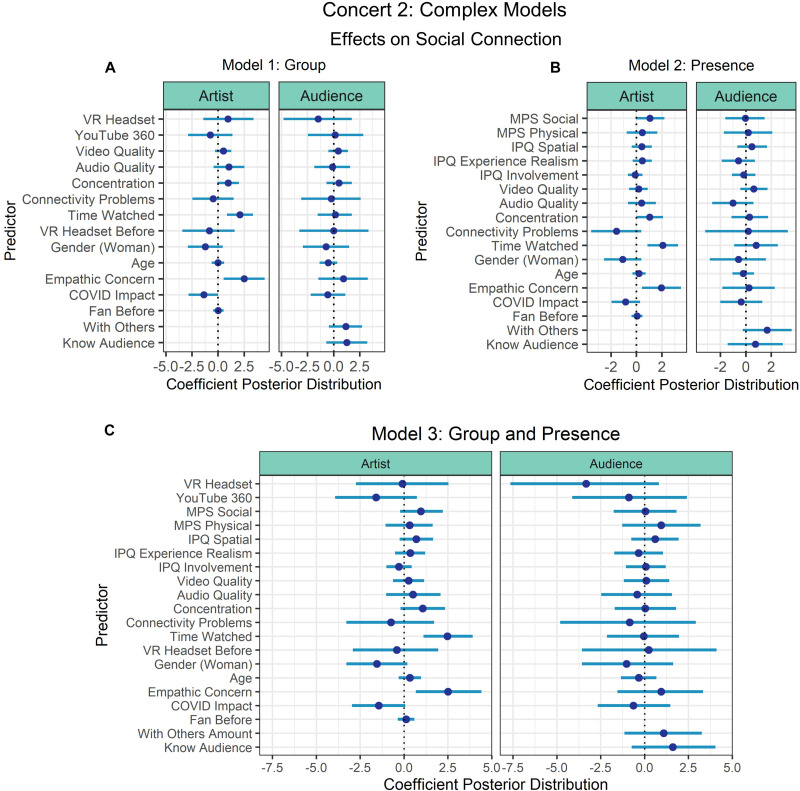
Concert 2’s complex models’ predictor coefficient estimates (points) and 90% credible intervals (lines) for the effects on social connection to the artist (left) and audience (right). Three models were fit to examine the individual effects of **(A)** Group and **(B)** Presence, and the third model examined the combined effects of Group and Presence **(C)**.

Three models were fit for each simple and complex selection of predictors to understand (1) the contribution of the experimental manipulation of group (YouTube vs. YouTube 360 vs. VR Headset) (Model 1: Group; see Figures 5, 6A), (2) the effects of only the presence variables (IPQ and MPS) (Model 2: Presence; see Figures 5, 6B), and (3) both group and presence variables together (Model 3: Group and Presence; see Figures 5, 6C). Model comparisons revealed that the models had approximately equal levels of fit (see Table 3).

When examining the simple models, Model 1: Group showed that usage of a VR headset predicted connection with the artist with at least 90% probability (90%-CI: 0.51–4.97), but when examining the combined effects of group and presence in Model 3, physical presence was the only predictor that did not intersect with 0 (90%-CI: 0.23–2.39).

When examining the complex models, to understand the effect of both group and presence predictors on social connection, we focused on the model that included all predictors of interest. Time watched (scaled) (90%-CI: 1.11–3.91) and empathic concern (90%-CI: 0.67–4.41) had credible intervals that did not intersect with 0, thus we can conclude with at least 90% posterior probability that these variables positively predicted social connection with the artist. All other predictors of social connection with the artist intersected with 0. In Models 1: Group and 2: Presence, time watched (scaled) and empathic concern still predicted connection with the artist. All other predictors intersected with 0 when predicting social connection with the audience.

#### Concert 3

**FIGURE 7:**
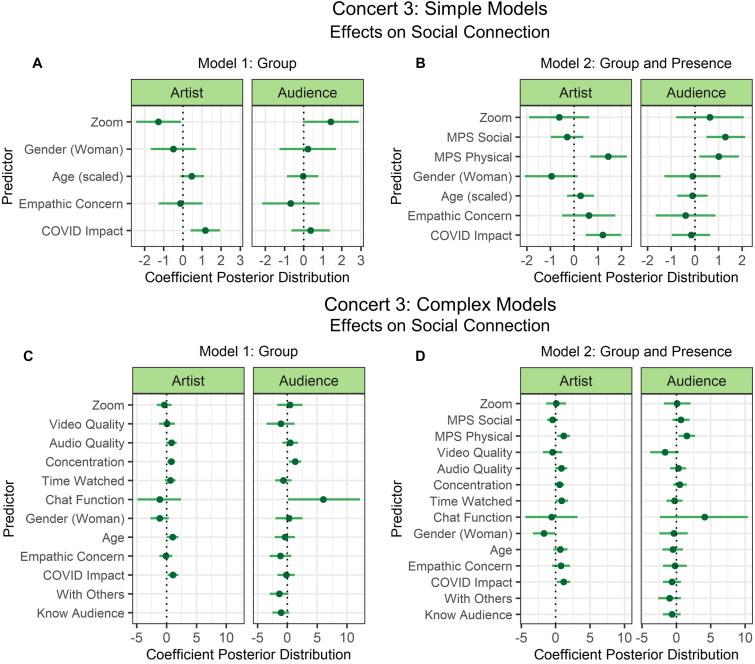
Concert 3’s simple **(A,B)** and complex **(C,D)** models’ predictor coefficient estimates (points) and 90% credible intervals (lines) for the effects on social connection to the artist (left) and audience (right). Two models were fit to examine the individual effect of the experimental manipulation from groups **(A,C)** and the combined effects of group and presence **(B,D)**.

Two models were fit for each simple and complex selection of predictors to examine the influence of the experimental manipulation (Zoom vs. regular YouTube) separately (Model 1: Group; see Figures 7A,C), and then together with the measures of social and physical presence (Model 2: Group and Presence; see Figures 7B,D). Model comparison revealed that Model 2 had much better predictive performance in the simple models and Model 2 had marginally better predictive performance in the complex models (see Table 3). Connectivity problems and fan-status were removed from the predictors, because there was only one participant who experienced connectivity problems, and only one participant was a fan before the concert. Therefore, the model was not fitting appropriately when these predictors were included. It should also be noted that very few people used the chat function (Zoom: *n* = 2; YouTube: *n* = 1), which possibly explains the very large credible interval for this predictor.

When examining the simple models, we can conclude with at least 90% probability that Zoom negatively predicted social connection with the artist (90%-CI: –2.44 to –0.09). Zoom predicted social connection with the audience with nearly 90% posterior probability (90%-CI: –0.03 to 2.88). When presence was included in the modeling, the credible interval of Zoom’s effect on connection with the artist crossed 0 and instead social presence predicted connection with the audience (90%-CI: 0.49–2.13), while physical presence predicted connection with both the audience (90%-CI: 0.20–1.87) and artist (90%-CI: 0.69–2.23) with at least 90% probability.

When examining the complex models, Model 2 with the predictors of Zoom (as compared to YouTube) and presence, showed that physical presence positively predicted social connection with the artist (90%-CI: 0.23–2.10) and the audience (90%-CI: 0.33–2.75) with at least 90% probability. Social connection with the artist was positively predicted by the impact of COVID-19 (90%-CI: 0.19–2.11), time watched (scaled) (90%-CI: 0.01–1.80), and negatively predicted by being a woman (90%-CI: –3.26 to –0.11) with at least 90% probability. All other predictors intersected with 0.

In Model 1, when presence predictors were not included, concentration additionally positively predicted social connection with the artist (90%-CI: 0.14–1.44) and audience (90%-CI: 0.31–2.35) with at least 90% probability. Social connection to the artist was also predicted by age (scaled) (90%-CI: 0.05–1.98), and social connection to the audience was predicted by usage of the chat function (90%-CI: 0.11–12.18). In contrast to Model 2, time watched (scaled) and being a woman had credible intervals that intersected with 0 in Model 1. However, the impact of COVID-19 was still a positive predictor of connection to the audience (90%-CI: 0.19–1.96).

#### Overall Models

**FIGURE 8:**
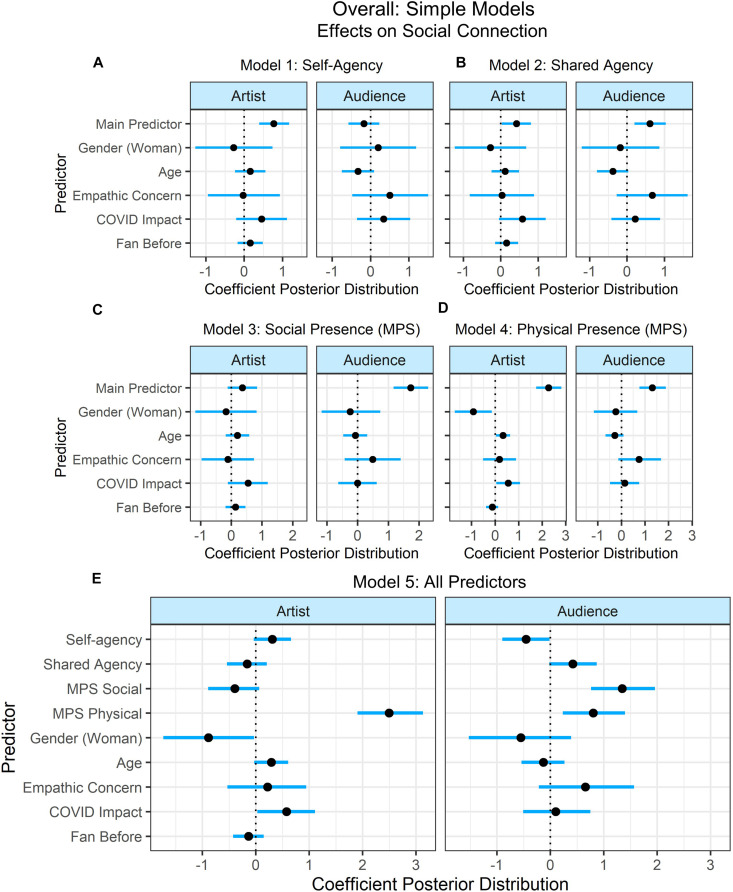
Overall simple models’ predictor coefficient estimates and 90% credible intervals using data from all concerts to determine the effects on social connection to the artist (left) and audience (right). Five models were fit to examine the individual contribution of the variables of interest: **(A)** Self-agency, **(B)** Shared Agency, **(C)** Social Presence, and **(D)** Physical Presence, and the combined effects of all predictors **(E)**. Lines indicate the 90% credible intervals (i.e., there is a 90% posterior probability that the coefficient of the predictor lies in that range). Credible intervals not intersecting 0 indicate that there is at least a 90% probability that the effect of that predictor on social connection is positive or negative.

**FIGURE 9:**
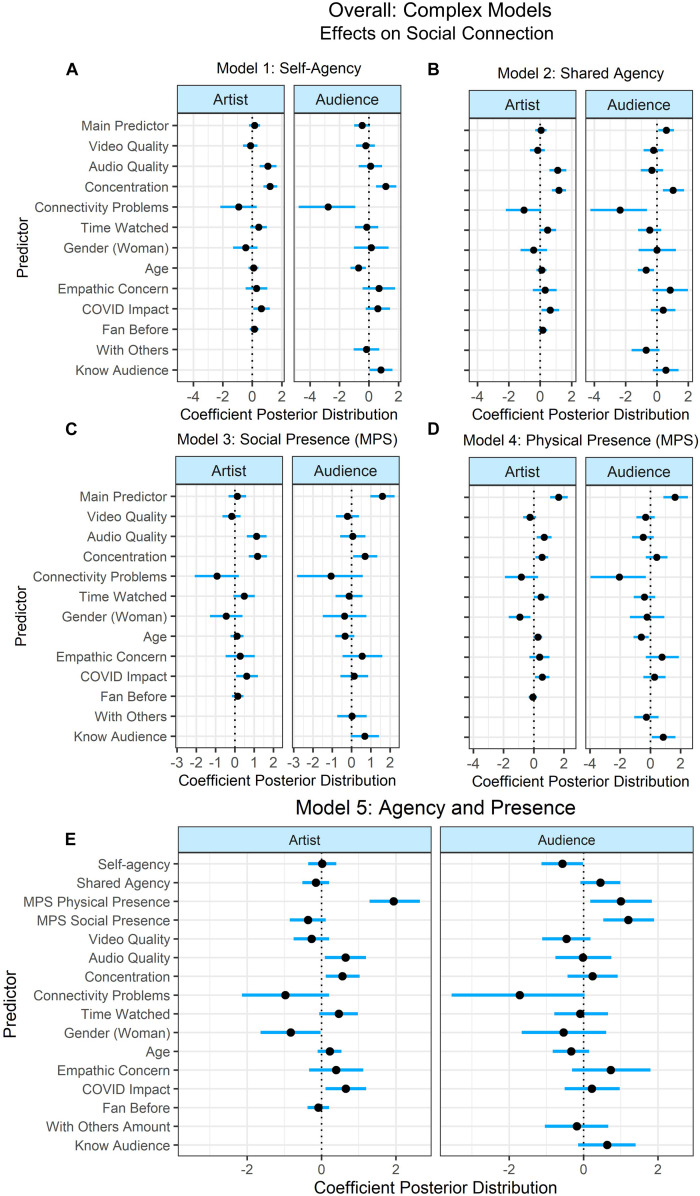
Overall complex models’ predictor coefficient estimates and 90% credible intervals using data from all concerts to determine the effects on social connection to the artist (left) and audience (right). Five models were fit to examine the individual contribution of the variables of interest: **(A)** Self-agency, **(B)** Shared Agency, **(C)** Social Presence, and **(D)** Physical Presence, and the combined effects of all predictors **(E).**

Four models were fit with each a simple and complex selection of predictors for the variables that were collected at every concert (see Figures 8, 9): (1) self-agency, (2) shared agency, (3) social presence, and (4) physical presence. Some participants attended more than one concert, therefore the crossed random effects for participants and concerts were estimated. Model comparison was unimportant for evaluating between these models, therefore only the estimates of the predictors’ effects are reported. To understand the effects of these predictors across all concerts, we examined the effect of each variable of interest individually, and then in relation to the model in which all predictors were included.

When examining the simple models, self-agency positively predicted connection with the artist in Model 1: Self-Agency (90%-CI: 0.39–1.17), but in Model 5: All Predictors, it negatively predicted connection with the audience (90%-CI:–0.90 to –0.02). In Model 2: Shared Agency, shared agency positively predicted connection with the artist (90%-CI: 0.03–0.81) and the audience (90%-CI: 0.20–1.04) with at least 90% posterior probability, while in Model 5, shared agency positively predicted connectedness with the audience with nearly 95% posterior probability (90%-CI: –0.02 to 0.87). Social presence predicted connection with the audience in Model 3: Social Presence (90%-CI: 1.17–2.30) and in Model 5: All Predictors (90%-CI: 0.76–1.96). Physical presence predicted both connection with the artist (90%-CI: 1.75–2.81) and audience (90%-CI: 0.77–1.89) in Model 4: Physical Presence and Model 5: All Predictors (artist: 90%-CI: 1.91–3.13; audience: 90%-CI: 0.23–1.40). COVID impact predicted connection to the artist in Model 4: Physical Presence (90%-CI: 0.06–1.06) and Model 5: All Predictors (90%-CI: 0.03–1.11). All other predictors intersected with 0.

When examining the complex models, Model 1: Self-Agency showed that self-agency’s credible interval intersected with 0 when predicting connection to the audience (90%-CI: –0.22 to 0.54); however, in Model 5: All Predictors, self-agency negatively predicted social connection with the audience (90%-CI:–1.13 to –0.01). In Model 2: Shared Agency, shared agency predicted social connection with the audience with at least 90% probability (90%-CI: 0.09–1.1). However, in Model 5: All Predictors, the credible interval of shared agency intersected with 0 (90%-CI: –0.51 to 0.20). Including the other predictors in the model with all predictors caused the effect of shared agency to become more negative (albeit while crossing 0) which is likely because shared agency is correlated with other predictors of interest (e.g., self-agency) (Knaeble and Dutter, 2017). In Model 3: Social Presence, social presence predicted connection with the audience (90%-CI: 0.97–2.24), and this was also the case in Model 5: All Predictors (90%-CI: 0.53–1.90). In Model 4: Physical Presence, physical presence predicted connection with both the artist (90%-CI: 1.10–2.26) and the audience (90%-CI: 0.86–2.50), and this continued to predict both connection to the artist (90%-CI: 1.29–2.65) and audience (90%-CI: 0.18–1.83) in Model 5 as well. In addition to the main predictors of interest, there were several predictors with credible intervals that did not intersect with 0 in the model with all predictors. Specifically, connection to the artist was positively predicted by perceived audio quality (90%-CI: 0.09–1.20), concentration (90%-CI: 0.11–1.03), and the impact of the COVID-19 pandemic (90%-CI: 0.11–1.21), while social connection with the artist was negatively predicted by being a woman (90%-CI: –1.63 to –0.03) with at least 90% probability.

There were several predictors that had credible intervals intersecting with 0 in Model 5: All predictors, but that predicted social connection with at least 90% probability in the other models with a single main predictor. Specifically, age negatively predicted social connection to the audience in Model 1: Self-agency (90%-CI: –1.25 to –0.20), Model 2: Shared Agency (90%-CI: –1.20 to –0.18), and Model 4: Physical Presence (90%-CI: –1.11 to –0.11), however, its credible intervals intersected with 0 in Model 5 (90%-CI: –0.83 to 0.15). Similarly, connectivity problems negatively predicted connection to the audience in Model 1: Self-agency (90%-CI: –4.77 to –0.92), Model 2: Shared Agency (90%-CI: –4.24 to –0.63), and Model 4: Physical Presence (90%-CI: –3.97 to –0.30), but its credible intervals intersected with 0 in Model 5 (90%-CI: –3.55 to 0.03). Concentration predicted social connection to the audience in Model 1: Self-agency (90%-CI: 0.49–1.86), Model 2: Shared agency (90%-CI: 0.38–1.74), and Model 3: Social Presence (90%-CI: 0.09–1.34), but not in Model 5 (90%-CI: –0.43 to 0.92). Whether participants knew other audience members personally (Know Audience) predicted social connection with the audience in Model 1: Self-agency (90%-CI: 0.03–1.60) and Model 4: Physical Presence (90%-CI: 0.09–1.67), but not in the model with all predictors (90%-CI: 0.09–1.34). Time watched (scaled) predicted connection with the artist in Model 4: Physical Presence (90%-CI: 0.01–0.98), but not in the other models. All other predictors intersected with 0.

### Correlational Analyses

**FIGURE 10:**
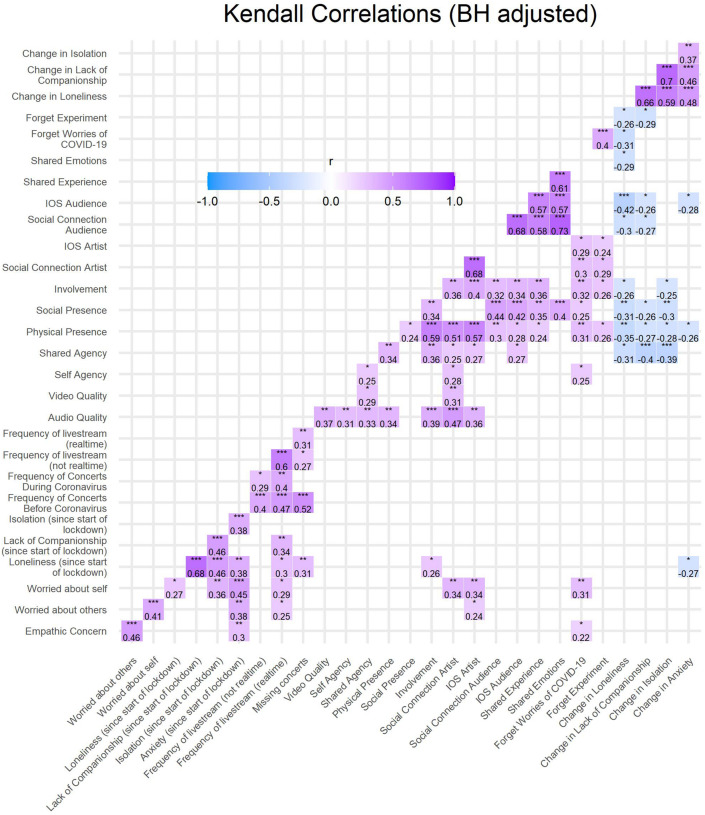
Change in anxiety, isolation, lack of companionship, and loneliness represent questions posed to the audience after each concert where they were asked to what extent they felt these emotions more or less after the concert (1 A lot less – 5 A lot more). Therefore, for example, participants tended to feel less lonely, less lack of companionship, and less isolation when they experienced more social and physical presence, and more physical presence was also associated with less anxiety. Forget experiment represents the item inquiring if the participant forgot they were participating in an experiment. Frequency of livestream (not realtime) and (realtime) represents the frequency that participants attended virtual concerts in the month prior to their registration for the study. Worried about self and worried about others were measures collected during registration that represent how often participants felt worried for their own well-being or other people during the pandemic, respectively. Variables that did not have any significant correlations (and therefore do not appear in Figure 10) include the number of hours listening to music per day, and age.

Kendall correlations were performed across the numeric variables that were collected from every participant during concert registration or after every concert. This was done to explore connections between variables collected during registration relating to well-being during the COVID-19 pandemic (e.g., loneliness, anxiety), real and livestream concert behavior before and during the pandemic, and participants’ experiences during the concert. In particular, participants reported their feelings of involvement (“During the concert I felt involved with the concert.” 1 Do not agree at all – 5 Agree completely), the extent to which they felt they shared emotions with other virtual audience members (1 Not at all – 5 Very much), and the extent to which they felt that they shared the experience with other virtual audience members (1 Not at all – 5 Very much). This was additionally investigated in relation to the measures of presence and agency. Significant correlations and their Kendall’s tau correlation coefficients are displayed in Figure 10. *P*-values were adjusted using the BH false discovery rate method (Benjamini and Hochberg, 1995).

## Discussion

The main aim of this study was to examine how sense of agency, sense of presence, and social context may facilitate social connectedness in virtual concerts. Three livestreamed concerts were hosted in the summer of 2020 when social restrictions of the COVID-19 pandemic resulted in increased popularity of virtual concerts. The following sections provide deeper understanding of our results.

### Impact of Agency, Presence, and Social Context on Social Connectedness

Providing participants with the ability to vote for the final song was unsuccessful in manipulating participants’ feelings of agency (see Supplementary Material 8 for subjective positive/negative effect on concert experience). One possible explanation for this could be that the manipulation was a short one-off event, while the manipulation of agency might be more successful in a paradigm where this is manipulated on a continuous scale (e.g., giving control over musical aspects such as increasing the volume produced by one of the artists), or where it is measured immediately after manipulation. Alternatively, it could be that voting resulted in being distracted from the concert experience, as this is not something that typically occurs at a concert. Nevertheless, social connection with the artist was predicted by whether their preferred song was played, regardless of their ability to vote. This result should be interpreted with caution, as participants who did not vote provided this information after the concert. However, this could hint at a more agreeable assessment of the performer, or could possibly relate to heightened feelings of shared agency in participants who considered the outcome of the vote (i.e., a shared task) to be successful. Such an interpretation is in line with previous research on feelings of agency and success (Tennen and Sharp, 1983; Aarts et al., 2005). Indeed, shared agency was found to predict connectedness with the performer in the simple model (Figure 3E) as well, but intersected with 0 in the complex model (Figure 4E). Further investigation is needed to understand the underlying mechanisms. Nevertheless, musicians can possibly leverage these findings by performing songs that are fan favorites to foster a sense of connection with the audience.

The aim of the second concert was to manipulate sense of presence. Participants that used a VR headset experienced more presence than those who watched a regular YouTube livestream. Examining the differences in the presence questionnaires’ subscales indicated that these differences could be attributed to physical/spatial presence and involvement. The simple models indicated that using a VR headset predicted connectedness with the artist, however, this effect was driven by changes in physical presence. This finding is aligned with other research showing that the usage of virtual reality facilitates greater feelings of presence and connectedness than 2D viewing (Elsey et al., 2019; Shin et al., 2019; Dekker et al., 2020), and highlights that immersive concert technologies enhance virtual concert experiences (Charron, 2017). However, when examining more confounding variables in the complex models, the amount of time participants spent watching the concert and participants’ empathic concern were more important predictors of the connectedness to the artist than VR headsets and physical presence. As time watching increased, it is likely that understanding of the musicians’ roles in the trio and the musical improvisation increased as well. A possible explanation for increased empathic concern resulting in more social connection with the artist can possibly be found in research demonstrating that empathy is a predictor of parasocial interactions (i.e., a unidirectional interaction in which, for example, an audience believes a relationship with the performer to be reciprocal (Horton and Richard Wohl, 1956; Dhanda, 2009). However, this does not explain why empathic concern was only a predictor in the second concert. The specificity of empathic concern in this concert could possibly be related to the improvisational nature of the performance. Neuroscientific research suggests that improvisation is facilitated by decreased self-monitoring, top–down cognitive control and greater expression of the self, which makes improvisation a rather vulnerable form of performance (Landau and Limb, 2017). Recent research suggested that empathic concern made listeners of an improvised jazz concert feel emotions similar to the performer’s emotions when listening to the audio recording online, however empathic concern did not facilitate shared emotions in the live concert (Leshem and Schober, 2020). Therefore empathic concern may facilitate greater emotional understanding in digital environments, which may make audience members high in empathy feel more connected to the performer in virtual concerts. However, this hypothesis should be tested directly with future research.

In the third concert, social context was manipulated. Participants attending via Zoom experienced greater social presence than those attending via a regular YouTube livestream. This difference may be explained by participants being more aware of a social setting in Zoom, as they saw front views of other audience members. Furthermore, Zoom negatively predicted connectedness with the artist in the simple models, and Zoom positively predicted connectedness to the audience with nearly 90% posterior probability (see Figure 7A). It is possible that viewing other audience members distracted from the performance and reduced connectedness to the performer while facilitating greater feelings of connectedness to other audience members. When presence measures were included in the model, the effect of Zoom disappeared. Instead, physical presence predicted connectedness to the performer and audience, while social presence predicted connectedness to the audience (see Figure 7B). In the complex models physical presence remained an important predictor of connectedness with the performer and the audience (see Figure 7D). However, the effects of social presence were reduced. Nevertheless, it is noteworthy that Zoom has been widely used as a concert platform during the COVID-19 pandemic. This is the case for children and parents (Sandra and Kusuma, 2020), patients, caregivers, and hospital staff (Ambler et al., 2020), as well as for choirs and other groups of joint music makers who have used Zoom as a virtual rehearsal space (Grushka et al., 2021; Onderdijk et al., 2021). This previous research suggests that connectedness is a central theme when examining Zoom’s efficacy (Ambler et al., 2020; Grushka et al., 2021). In the present study performers and participants did not take full advantage of Zoom’s features (e.g., only two participants used Zoom’s chat function), and it is likely such communicative features can be further exploited to facilitate connectedness among/between audiences and performers.

Overall models were created to examine the impact of agency and presence on participants’ experiences regardless of their experimental condition. Examining the individual contribution of each predictor in the simple models suggested that feelings of self-agency predicted connectedness with the artist, feelings of social presence predicted connectedness with the audience, and feelings of shared agency and physical presence predicted connectedness with both the artist and audience. When all predictors were included in the model, self-agency negatively predicted connectedness with the audience, social presence predicted connectedness with the audience, and physical presence predicted connectedness with both the audience and the artist. This was the same in the complex models. However, when presence was included in the models with agency, the benefits of agency on connectedness were reduced. This is probably because a sense of control in a virtual environment is tightly linked to a sense of being there (Zahorik and Jenison, 1998), and other research has suggested that presence in virtual environments is linked to feelings of agency (Herrera et al., 2006; Riva, 2008; Lallart et al., 2009; Kothgassner et al., 2018; Riches et al., 2019). However, self-agency was not correlated with measures of presence in the current study, and instead only shared agency was correlated with physical presence. Self-agency negatively predicted connectedness to the audience possibly because feelings of solitary control in a virtual environment may preclude feelings of a shared experience and feelings of connectedness with other audience members. Shared agency predicted connectedness to the audience with nearly 95% posterior probability possibly because a sense of shared control facilitates feelings of connectedness to others. The way in which self-agency negatively predicted connection to the audience, while shared agency positively predicted connection to the audience, is in accordance with an existing theory on self- and shared agency which suggests that these are opposing states within a framework of joint action (Pacherie, 2014). However, self-agency and shared agency were also correlated. Therefore, a more nuanced perspective that views these as complimentary feelings as opposed to oppositional may be warranted (Salmela and Nagatsu, 2017).

Furthermore, previous research has demonstrated the importance of feelings of social presence in facilitating connectedness in virtual environments (Rettie, 2003; Dey and De Giizman, 2006; IJsselsteijn et al., 2007). However, the current research revealed that in the context of virtual concerts, physical presence may be an even more important contributor to connectedness between audiences and both performers and other audience members. Given that the VR headset effectively manipulated physical presence, concert organizers could aim to provide virtual reality viewing options for virtual concertgoers to facilitate feelings of connectedness with performers. Since 3D sound also increases social presence, future research could investigate whether this additionally promotes physical presence and connectedness (Shin et al., 2019).

Moreover, physical presence was correlated with a number of variables including perceived audio quality, involvement, feeling that participants shared experiences with other audience members, and shared agency. Therefore, it is possible that feeling physical presence made participants feel as if they were together and experiencing the same virtual ‘here and now’ as the artist and other audience members. The correlation with audio quality compliments previous research suggesting audio quality may contribute to a sense of ‘being there’ (Lessiter and Freeman, 2001; Nordahl and Nilsson, 2014). Additionally, in line with other research conducted during the COVID-19 pandemic (Swarbrick et al., accepted), the current study found that perceived audio quality also promoted social connectedness to the performer. In contrast to these findings, previous research suggested that audio quality is not an important contributor to music video enjoyment (Schoeffler and Herre, 2014), however, it could be that feelings of connectedness are distinct from enjoyment, or that there are differences in listeners’ expectations of virtual concerts and music videos. Moreover, it should be considered that quality is possibly reduced when streaming or listening through a personal device. It is possible that audio quality facilitates improved physical presence, which subsequently facilitates connectedness with the performer. However, to determine causality, future research should aim to directly manipulate audio quality in virtual concerts. On the other hand, perceived video quality did not predict social connectedness. Yet, VR headsets effectively increased feelings of physical presence and connectedness. Participants reported that video quality was generally worse in the VR headset condition than when viewing the normal YouTube stream. This is likely because the normal YouTube video in both concert 1 and 3 was of higher definition than the videos of concert 2 (see Supplementary Material 9). Therefore, the benefits of VR headsets cannot be explained by video quality, but rather are explained by enhanced physical presence (Elsey et al., 2019).

Additionally, the effects of individual characteristics (e.g., gender, age, empathic concern), and potentially confounding variables were modeled (e.g., concentration levels, connectivity problems, time watched). Interestingly, women experienced less social connection towards the artists, possibly because women generally feel less presence in virtual environments than men (Felnhofer et al., 2012), or because gender interacts with the types of loneliness that foster engagement in parasocial interactions (Wang et al., 2008). Several models that did not include all main predictors of interest had confounding predictors that did not intersect 0. Specifically, in several models, age and connectivity problems negatively predicted connectedness to the audience. As age increased, participants reported less social connectedness with the audience, possibly because older participants do not have as much experience bonding with peers over social media platforms or in virtual environments. Little research has examined the efficacy for social media to promote connectedness on populations outside the age of adolescents and young adults (Ryan et al., 2017), therefore the present findings contribute to this gap in research by showing that age is an important consideration when examining connectedness in virtual environments. Concentration was a predictor of connectedness to the artist and audience in several models, possibly because concentrated participants were more aware of the virtual environment and attention may be necessary for facilitating feelings of presence and connectedness. Finally, whether participants knew other audience members predicted connectedness with the audience, likely because participants already had social connections with those individuals they were acquainted with.

### The COVID-19 Pandemic

Our findings show that the negative impact of the COVID-19 pandemic predicted social connectedness with the artist, but not the audience. This is aligned with other research showing that reminders of the COVID-19 pandemic during virtual concerts led to greater feelings and behaviors (e.g., sharing posts and commenting) of social connectedness (Swarbrick et al., accepted). This may be related to how reminders of threats temporarily strengthen group bonding (Jackson et al., 2019; Gelfand et al., 2020). However, this does not explain the specificity of the bonding with the artist and not the audience in the present study. A possible explanation can be found in literature on parasocial interaction. Parasocial interaction is a term coined by Horton and Wohl that describes a unidirectional interaction (e.g., a person watching a performer sing a song on television), in which the audience might feel as though they are engaged in a reciprocal relationship (Horton and Richard Wohl, 1956; Rubin and McHugh, 1987; Kurtin et al., 2019). Studies show lonely people report more parasocial interaction, which is likely due to parasocial interaction acting as a social surrogate for real social interaction, and at least partially fulfilling social needs (Wang et al., 2008; Dhanda, 2009). Therefore, people who are unable to fulfill their social needs due to the COVID-19 pandemic may be trying to substitute their lack of real-life social interaction for parasocial interactions with performers. This is supported by our findings that frequency of attending livestreamed concerts in realtime during the pandemic was correlated with loneliness, lack of companionship, and worries about oneself and others. Interestingly, these measures were not correlated with the frequency of attending non-realtime livestreamed concerts. This indicates that participants may seek out real-time livestreamed concerts because they may offer greater social satisfaction. This is supported by other research on virtual concerts that showed that attending livestreamed concerts in realtime promotes greater social connection than viewing pre-recorded concerts (Swarbrick et al., accepted). This is likely because viewers recognize that they are sharing the same experience at the same time and ‘place’ as the performer and audience, albeit virtually (Swarbrick et al., accepted). In the present study, feelings of shared experience and shared emotions with other audience members were correlated with feelings of connectedness with the audience.

Before social distancing restrictions, concerts were a source of COVID-19 outbreaks (Koizumi et al., 2020). However, with adequate ventilation, assigned seating, contact tracing, and hygienic equipment and precautions, concerts present a low risk for the transmission of COVID-19 (Moritz et al., 2020). Nonetheless, attending a real concert would present greater risk than watching a virtual concert. The correlational analyses suggested that frequent concertgoers watched more online concerts and were more likely to risk going to real concerts with safety precautions. The frequency of watching livestreamed concerts (both in realtime and not in realtime), and the frequency of attending concerts before the pandemic were also correlated with the extent participants missed going to concerts. Missing attending concerts was further correlated with feelings of loneliness and lack of companionship since the beginning of the pandemic, which may be related to previous research on how concert attendance promotes social well-being (Brown and Knox, 2017). Alternatively, participants who missed attending concerts may have used concerts as their source of companionship before the pandemic, and the sudden loss of this important sociocultural event led them to lose their main source of social fulfillment.

Music has been used for its anxiolytic effects in healthcare settings and there is empirical evidence that live concerts reduce stress on a physiological level (Nilsson, 2008; Fancourt and Steptoe, 2018). In the present study, participants reported the extent to which they forgot their worries related to COVID-19. Participants’ experiences of self-agency, physical presence, social presence, involvement, and connectedness to the artist, but not the audience, were all related to forgetting worries related to the pandemic. This suggests that parasocial interaction with the artist may facilitate reduced worries more than connecting with fellow audience members. These findings contribute to the research on music as a social surrogate by positioning the musician as an important contributor in music’s ability to alleviate loneliness (Schäfer et al., 2020). Forgetting worries after the concert was also correlated with reduced feelings of loneliness after the concert and to feeling worries about oneself reported before the concerts during registration. Therefore it could be that individuals who are more worried benefit more from virtual concert attendance because they receive much needed social stimulation that alleviates loneliness and reduces worries. Interestingly, empathic concern was also correlated with forgetting worries surrounding COVID-19 during the concert, which suggests that empathy might play a role in how we experience, and are susceptible to change in, worrisome feelings during pandemic times.

Lastly, similar to forgetting worries related to COVID-19, reductions in loneliness, isolation, and lack of companionship after concert attendance were associated with feelings of shared agency, physical presence, social presence, and involvement. Reduced anxiety was related to greater connectedness to the audience and physical presence. Social connectedness with the audience, but not the artist, was correlated with reduced loneliness, lack of companionship, and anxiety which suggests that feeling connected to other audience members is also an important component of virtual concert attendance. Real concert attendance may be motivated by a sense of community among audience members (Dearn and Price, 2016). Similarly, previous research shows that viewing livestreams of content as varied as videogaming, eating, and dancing is often motivated by social interaction, meeting new people, and a sense of community (Friedländer, 2017; Hilvert-Bruce et al., 2018). However, other research conducted during the pandemic suggested that the frequency of virtual social interactions was not related to well-being (Towner et al., 2021). Therefore, virtual concerts might hold a special capability to promote well-being in contrast to other virtual interactions. The correlations suggest that to reduce audience members’ loneliness and improve their well-being, concert organizers and musicians should aim to foster feelings of presence, agency, and connectedness.

### Limitations and Future Research

Several limitations exist in the present study. Firstly, self-reported measures were used for all variables, including those that may confound results (e.g., concentration, time watched). Future work could measure these objectively using eye-tracking or video analysis. Secondly, the exploratory nature of this research, exemplified by small sample sizes, means that results should be interpreted with caution. The physically present group was excluded from inferential analyses due to the very small sample size (*n* = 6). These participants also reported confusion when responding to the presence questionnaires that were designed for a virtual audience. Future investigations comparing virtual and real-world experiences could consider developing questionnaires that assess presence in both contexts with questions such as “How frequently did your mind wander?”, “How much were you paying attention?”, or “How much did you notice the other people in the audience?”.

Furthermore, the directionality of the results from Bayesian modeling needs to be interpreted with caution. Social connectedness and feelings of agency and presence were both measured after the concert. Therefore, it is unknown whether presence really caused greater feelings of connectedness, or whether the reverse is possible such that connectedness could have caused greater feelings of physical and social presence. However, the experimental manipulations’ effects on social connectedness can be inferred as causal, as participants were randomly assigned to the experimental conditions. These manipulations also caused changes in agency and presence. Future research should still aim to assess the direction of the relation between presence, agency, and connectedness.

Another important consideration is the extent to which a large portion of our results are generalizable and replicable outside of the context of the COVID-19 pandemic. When real-life concerts return after the pandemic, livestreamed concerts can be further compared with live concerts to understand differences in these experiences, and their effects on social connection. Here it should be noted that while our concerts tried to replicate basic ecological settings of livestreamed concerts, the easy and free online availability of professionally produced live concerts and livestreamed concerts may have influenced the expectations of the audience. For example, a small number of participants suggested the usage of moving cameras from multiple angles. These expectations should be considered when examining livestreamed concerts, as a static video image might be acceptable for some viewers but impeding the experience of others. Nevertheless, the current research provided valuable insights for future research to focus on in general, and specifically provided novel evidence that negative feelings related to COVID-19 can predict connectedness to the artist. This contributes to greater insight into peoples’ musical behavior during times of real-life social deprivation.

Lastly, as our study focused for a large part on music’s ability to improve well-being, and loneliness is related to a number of negative health outcomes (Hawkley and Cacioppo, 2010), future research could consider examining changes in biological and physiological markers of loneliness-induced stress in response to livestreamed concerts. Previous research suggests that attending real concerts reduces biological stress (Fancourt and Williamon, 2016), however, future research could examine if the same biological response occurs in the context of virtual concerts.

## Conclusion

In conclusion, the negative impact of the COVID-19 pandemic predicted social connectedness with the artist, but not the audience, likely because participants fulfilled their missing social needs with parasocial interactions. This research contributes to existing literature on music as a social surrogate. Physical presence was an important predictor of connectedness with the performers and audience members, while social presence was an important predictor of connectedness with only the audience. Thus, concert organizers and performers should aim to promote audience members’ feelings of presence. Virtual reality promoted feelings of physical presence, while Zoom promoted feelings of social presence. Audio quality was also an important predictor of connectedness towards the artist, which could be improved by both concert organizers and audience members alike. Physical presence, social presence, and shared agency were related to reduced negative feelings such as loneliness. Artists and concert organizers should leverage these results to try to foster a sense of shared agency, as well as physical and social presence among their audiences, to alleviate loneliness, increase social connectedness, and momentarily let people forget their worries surrounding the COVID-19 pandemic.

## Data Availability Statement

The datasets presented in this study can be found in an Open Science Foundation online respository: Open Science Foundation. Livestream Experiments: The Role of COVID-19, Agency, Presence, and Social Context in Facilitating Social Connectedness, https://osf.io/d3z8e/.

## Ethics Statement

The studies involving human participants were reviewed and approved by the Ethics Commission Faculty of Arts and Philosophy, Ghent University. The participants provided their written informed consent to participate in this study. Written informed consent was obtained from the individuals for the publication of any potentially identifiable images or data included in this article.

## Author Contributions

KO and DS participated in the design of the study. KO and BV carried out the study. KO, MM, and DS conducted the statistical analyses. KO and DS wrote the manuscript. JV, P-JM, and ML provided supervision throughout the process. All authors read and approved the final manuscript.

## Conflict of Interest

The authors declare that the research was conducted in the absence of any commercial or financial relationships that could be construed as a potential conflict of interest.

## Footnotes

1
https://youtu.be/3Scf3ewg0sQ

2
https://youtu.be/1I4cKAxKCYE

3
https://www.uio.no/ritmo/english/research/labs/fourms/software/musiclab-app/

4
osf.io/d3z8e/
