# Supplementary material for: Livestream Experiments: The Role of COVID-19, Agency, Presence, and Social Context in Facilitating Social Connectedness
Source: Front Psychol. 2021 May 24;12:647929. doi: 10.3389/fpsyg.2021.647929 (PMC8180877; doi:10.3389/fpsyg.2021.647929)

Supplementary Material

# Supplementary Material S1: Musical stimuli

## List of musical pieces concert 1

- Tarantella (composed by Alfonso Rendano)
- Asturias (composed by Isaac Albeniz)
- Libertango (composed by Astor Piazzolla, arrangement by Giusy Caruso)
- Danzas Argentinas (composed by Alberto Ginastera):
  - Danza del viejo boyero
  - Danza del gaucho matrero

## List of musical pieces concert 2

FLIRT. This was a completely improvised contemporary experimental piece on bass clarinet, violin, and percussion.

## List of musical pieces concert Concert 3

- Oyfn veg shteyt a boym (traditional piece, arrangement by Jonas De Rave)
- Byzantian melody (traditional piece, arrangement by Shalan Alhamwy)

*During the concert this was called Ya loor, but the artists revealed this was incorrect afterwards and referred to it as ‘Byzantian melody’.*

- Longa (composed by Wanees Wartenian, arrangement by Shalan Alhamwy)

*Similarly, the artists provide a longer title during the concert, but afterwards revealed it is simply called Longa.*

- Cote d’opale (composed by Jonas De Rave)
- Old Homs (composed by Shalan Alhamwy)

# Supplementary Material S2: Instructions and transcripts

## Concert 1

### Communication via email before concert: English

Here you can find the link to the livestream of the concert by Giusy Caruso:

<https://youtu.be/Ou191NRHBDs>

Do not share this link with others.

**Please watch the livestream on a laptop or desktop screen and listen via head-/earphones**, if you can.

**During the concert we will ask you to vote** on a last piece to be played by the artist. When this is mentioned during the concert, please **Click here to vote [hyperlink].**

If others are watching with you, please do not deliberate and only answer for yourself.

After the concert please answer the questions of the following questionnaire:

Click here for questionnaire [hyperlink].

Your **personal ID code is […]**.

Enjoy!

Kelsey

### Communication via email before concert: Dutch

Bij deze ontvangt u de link naar het livestream concert van Giusy Caruso:

​<https://youtu.be/Ou191NRHBDs>

Deel deze link niet met anderen.

**Gelieve het livestream concert via een laptop of computer scherm te bekijken en te luisteren via oordopjes/hoofdtelefoon**, als dit mogelijk is.

**Tijdens het concert zullen wij u vragen om te stemmen** op een laatste muziekstuk dat gespeeld zal worden door de artiest. Wanneer dit genoemd wordt tijdens het concert, gelieve **Hier te klikken [hyperlink] om uw stem uit te brengen**.

Indien u samen met anderen kijkt, bespreek uw keuze niet en beslis alleen voor uzelf.

Na het concert vragen we u om een vragenlijst in te vullen. Deze is te vinden via de volgende link: Klik hier voor vragenlijst [hyperlink].

Uw **persoonlijke ID code is […]**.

Veel plezier!

Kelsey

### Transcript introduction and explanation during concert

Each line was first delivered in Dutch, and then repeated in English, to accommodate for the varying languages of the audience members.

*Host* [00:08]: Ok, welkom iedereen. Mijn naam is Kelsey Onderdijk, ik ben een onderzoeker en jullie contactpersoon voor deze concertserie. We hebben vandaag een divers publiek dus ik zal straks ook eventjes eerst ons Engelstalige publiek verwelkomen. So, welcome everybody to our concert series. My name is Kelsey Onderdijk, I am a researcher and your contact person for this concert series. We have a global audience so I will first give my introduction in Dutch, and then I will come back to you in English. Dus vandaag beginnen we de concert serie met Giusy Caruso. Giusy Caruso is een musicologe, een concert pianiste, een onderzoeker, maar bovendien ook mijn collega hier bij het IPEM. Vandaag gaat zij vier muziekstukken voor ons spelen, die ze heeft uitgekozen in het kader van ‘Rhythm and Dance’. Na deze vier stukken zal ik eventjes terugkomen en hierna zal ze nog een laatste stuk voor jullie gaan spelen. So, welcome again to everybody. Today our concert series is kicked off by Giusy Caruso, and Giusy Caruso is a musicologist, a researcher, of course a concert pianist, and above all my colleague here at IPEM. She will start off with four musical pieces that she has picked out for you in the context of ‘Rhythm and Dance’. After these four pieces I will shortly come back, and after this, Giusy will play one last song for you. So please give a warm welcome from behind your computers, geef alstublieft een warm welkom vanachter jullie computers aan Giusy Caruso.

*Artist* [02:00]: Good evening. The program of tonight is a musical journey through the rhythm of dances from different countries. I will start playing a Tarantella, a typical dance from south Italy, my country. And I will play a Tarantella by Alfonso Rendano, written for piano. Alfonso Rendano is an Italian composer, who was born nearby my hometown Cosenza, in Calabria, in the late nineteenth century. From Italy we will move to Spain - to Andalusia, with flamenco style composition, written originally for piano by the composer Isaac Albéniz. But this piece is more famous in the guitar version made by Segovia. And from Spain we will move to Argentina, with the popular Libertango by Astor Piazzolla. We will remain in Argentina with another piece, the Argentinian dance by the masters of Astor Piazzolla. The composer Alberto Ginastera, who dedicated a series of dances, and the first of this is dedicated to the old cowboy, la Danza del viejo boyero. Enjoy.

*Host* [17:33]: Ok, sommigen van jullie hebben een uitnodiging gekregen om te stemmen op een laatste nummer. Zoals Giusy net vertelde, ze heeft het eerste stuk gespeeld van Ginastera - de Danzas Argentinas. En jullie kunnen nu gaan stemmen om, of het tweede stuk, of het derde stuk van de Danzas Argentinas te beluisteren. Dus ga alsjeblieft naar uw email en klik op het stemformulier om te gaan stemmen voor degene die dit hebben gekregen. Dan kunnen jullie kiezen uit Danza de la moza donosa, welke een nadruk heeft op de melodie. Of jullie kunnen stemmen op Danza del gaucho matrero, en deze heeft een nadruk op ritme. Dus ga alsjeblieft naar je email, en ga stemmen, en dan kom ik na het stemmen over ongeveer een tot twee minuutjes terug bij jullie.

*Host* [18:24]: So, some of you have received a link to vote for a last piece in your email. Giusy just told us she played the first of three dances, Danzas Argentinas by Ginastera. And now you can vote to either let her play the second piece, with is Danza de la moza donosa, which has an emphasis on melody, or Danza del gaucho matrero, which has an emphasis on rhythm. So please go to your emails and pick the song that you would like Giusy to play. Then I’ll be back in about one to two minutes with the results.

*Host* [23:14]: Ok, so the results are in. De resultaten zijn binnen. En Giusy zal spelen, met de meeste stemmen: Danza del gaucho matrero. Enjoy.

*Host* [26:47]: Thank you so much Giusy for playing for us. En natuurlijk hartelijk dank voor iedereen in het publiek die gekeken heeft en mee heeft gedaan aan dit concert. Vergeet niet om de vragenlijst voor na het concert in te vullen, en ik hoop jullie te zien in een van de andere concerten. So, thank you all very much for joining us for this concert. I hope you enjoyed it, please don’t forget to also fill out the questionnaire for after the concert, and I hope to see you in one of the other concerts that we are having. So yeah, see you then, tot ziens, bye.

## Concert 2

### Communication via email before concert: English

#### Group: standard 2D YouTube livestream

Here you can find the link to the livestream of the concert by Nemø:

<https://youtu.be/1I4cKAxKCYE>

​Do not share this link with others.

**Please watch the livestream on a laptop or desktop screen and listen via head-/earphones**, if you can.

After the concert please answer the questions of the following questionnaire:

Click here for questionnaire [hyperlink].

Your **personal ID code is […]**.

Enjoy!

Kelsey​

#### Group: 360 degrees view with laptop/computer screen

Here you can find the link to the livestream of the concert by Nemø:

<https://youtu.be/1I4cKAxKCYE>

​Do not share this link with others. This is a video recorded with a 360 camera. This means you can move through the image by clicking and dragging your cursor on the image.

**Please watch the livestream on a laptop or desktop screen and listen via head-/earphones**, if you can.

After the concert please answer the questions of the following questionnaire:

Click here for questionnaire [hyperlink].

Your **personal ID code is […]**.

Enjoy!

Kelsey​

#### Group: 360 degrees view with VR headset

Here you can find the link to the livestream of the concert by Nemø:

<https://youtu.be/1I4cKAxKCYE>

​Do not share this link with others.

Attached you will find instructions for the VR headset you have received. Please read these instructions before the concert. If there are any questions you can contact me.

After the concert please answer the questions of the following questionnaire:

Click here for questionnaire [hyperlink].

Your **personal ID code is […]**.

Enjoy!

Kelsey​

### Communication via email before concert: Dutch

#### Group: standard 2D YouTube livestream

Bij deze ontvangt u de link naar het livestream concert van Nemø:

<https://youtu.be/1I4cKAxKCYE>

Deel deze link niet met anderen.

**Gelieve het livestream concert via een laptop of computer scherm te bekijken en te luisteren via oordopjes/hoofdtelefoon**, als dit mogelijk is.

Na het concert vragen we u om een vragenlijst in te vullen. Deze is te vinden via de volgende link: Klik hier voor vragenlijst [hyperlink].

Uw **persoonlijke ID code is […]**.

Veel plezier!

Kelsey

#### Group: 360 degrees view with laptop/computer screen

Bij deze ontvangt u de link naar het livestream concert van Nemø:

<https://youtu.be/1I4cKAxKCYE>

Deel deze link niet met anderen.

**Gelieve het livestream concert via een laptop of computer scherm te bekijken en te luisteren via oordopjes/hoofdtelefoon**, als dit mogelijk is.

Na het concert vragen we u om een vragenlijst in te vullen. Deze is te vinden via de volgende link: Klik hier voor vragenlijst [hyperlink].

Uw **persoonlijke ID code is […]**.

Veel plezier!

Kelsey

#### Group: 360 degrees view with VR headset

Bij deze ontvangt u de link naar het livestream concert van Nemø:

<https://youtu.be/1I4cKAxKCYE>

Deel deze link niet met anderen.

**Bijgaand vindt u de instructies** voor de VR headset die u heeft ontvangen. **Loop deze voor het concert een keer over.** Mochten er vragen zijn kunt u mij contacteren.

Na het concert vragen we u om een vragenlijst in te vullen. Deze is te vinden via de volgende link: [Klik hier voor vragenlijst.](https://forms.office.com/Pages/ResponsePage.aspx?id=3hyB1-_sbEmPkaF4YkG5nALXsL0-KLJNtpd29vb7EehUNVFJUzlGWTBHUDhESzI0R0o1NVNDVUFURi4u)

Uw **persoonlijke ID code is D107**.

Veel plezier!

Kelsey

### Instructions VR headset

Before you start, make sure your phone is charged!

Try it out before the concert itself with one of the following links (these are just examples):

<https://www.youtube.com/watch?v=hNAbQYU0wpg&t=87s> (rollercoasters)

<https://www.youtube.com/watch?v=v64KOxKVLVg> (underwater)

<https://www.youtube.com/watch?v=pCve1w1GFOs> (flying to the edge of space)


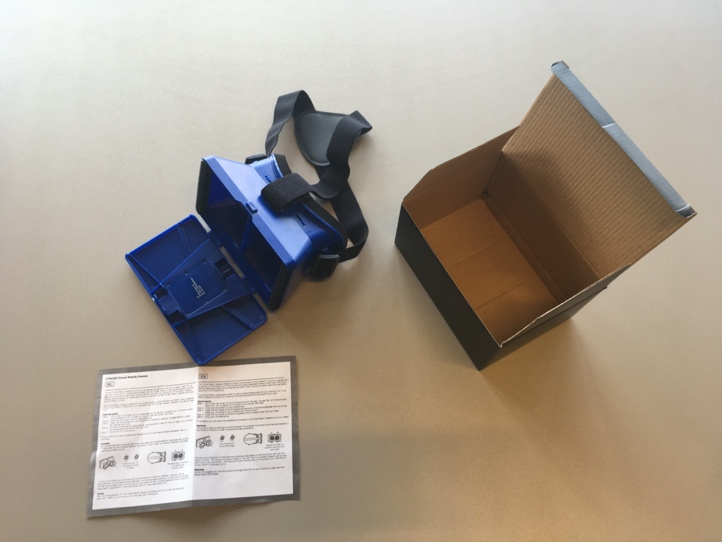


1.

Opening the box should give you a headset
 and leaflet with some info.


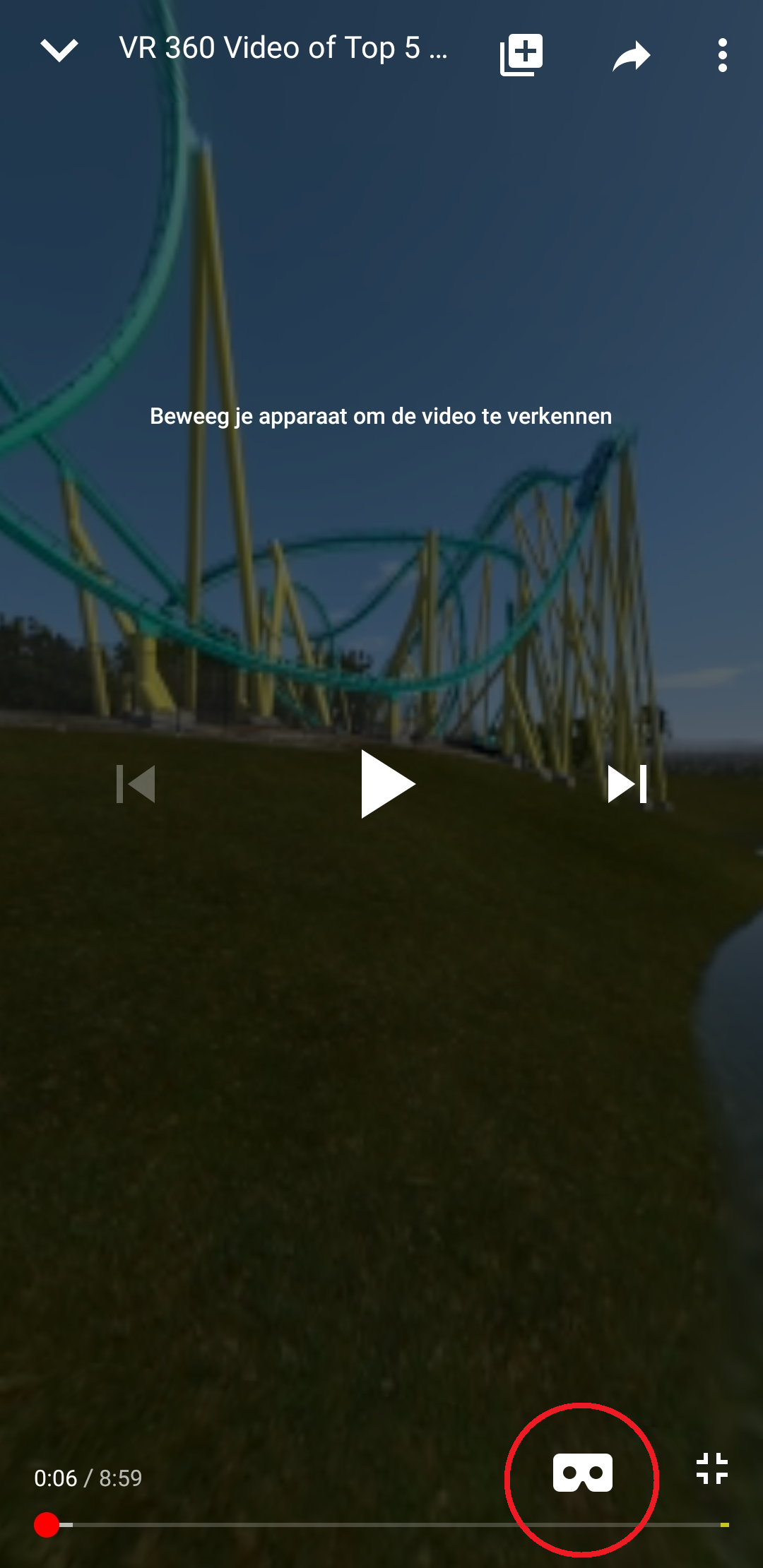


2.

When you open the link in the YouTube app your screen will look something like this. Click the VR glasses icon at the bottom (indicated by the red circle).


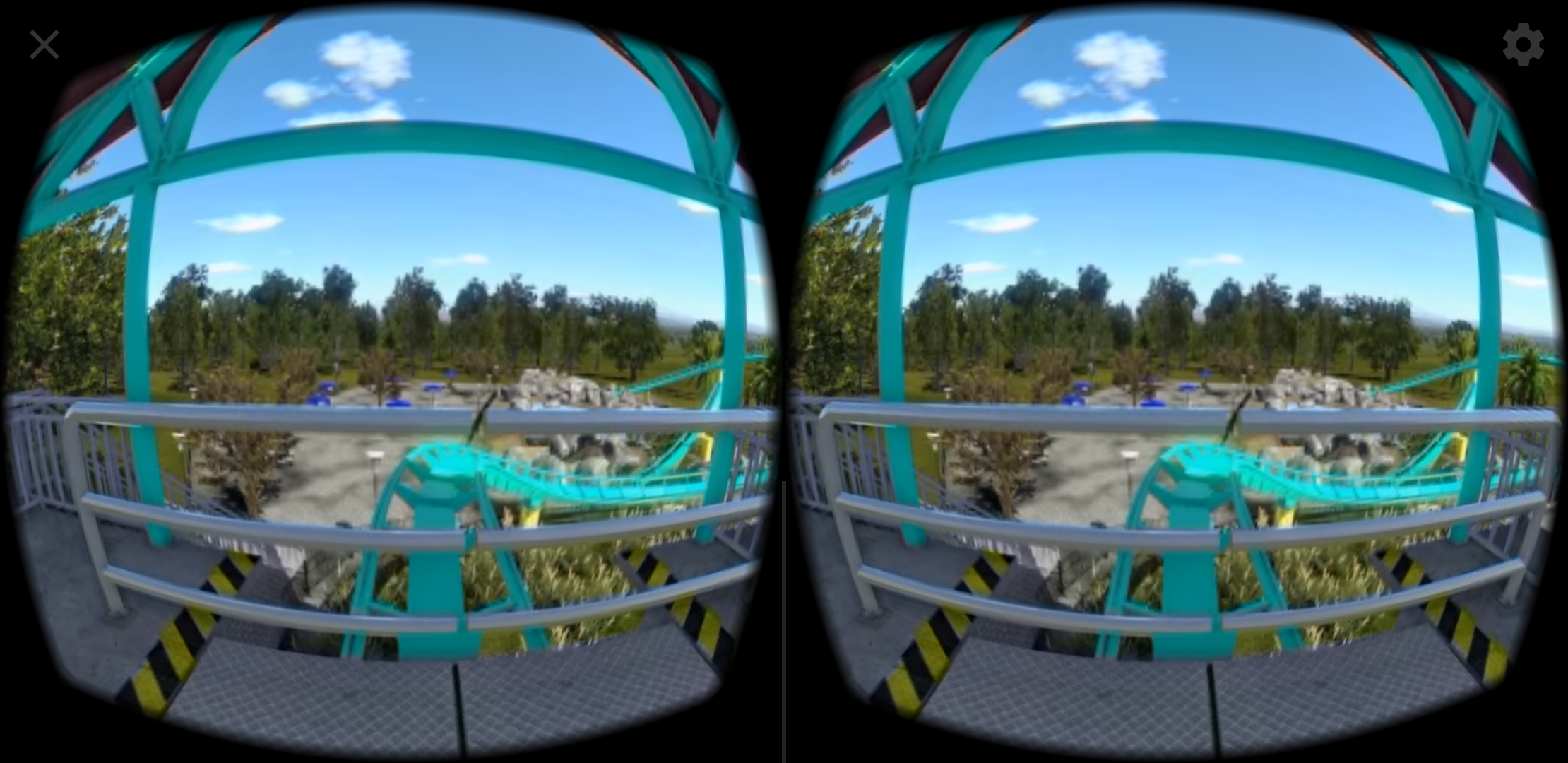
3.

This will give you the following view. Now you are ready to put the phone in the VR glasses!

4. You click your phone into the plastic holders.


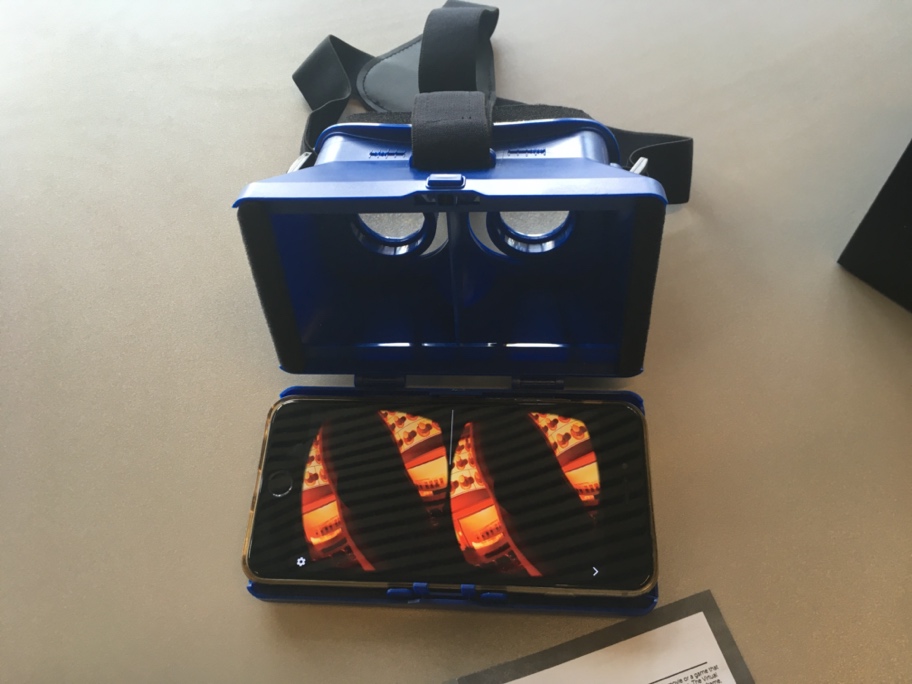


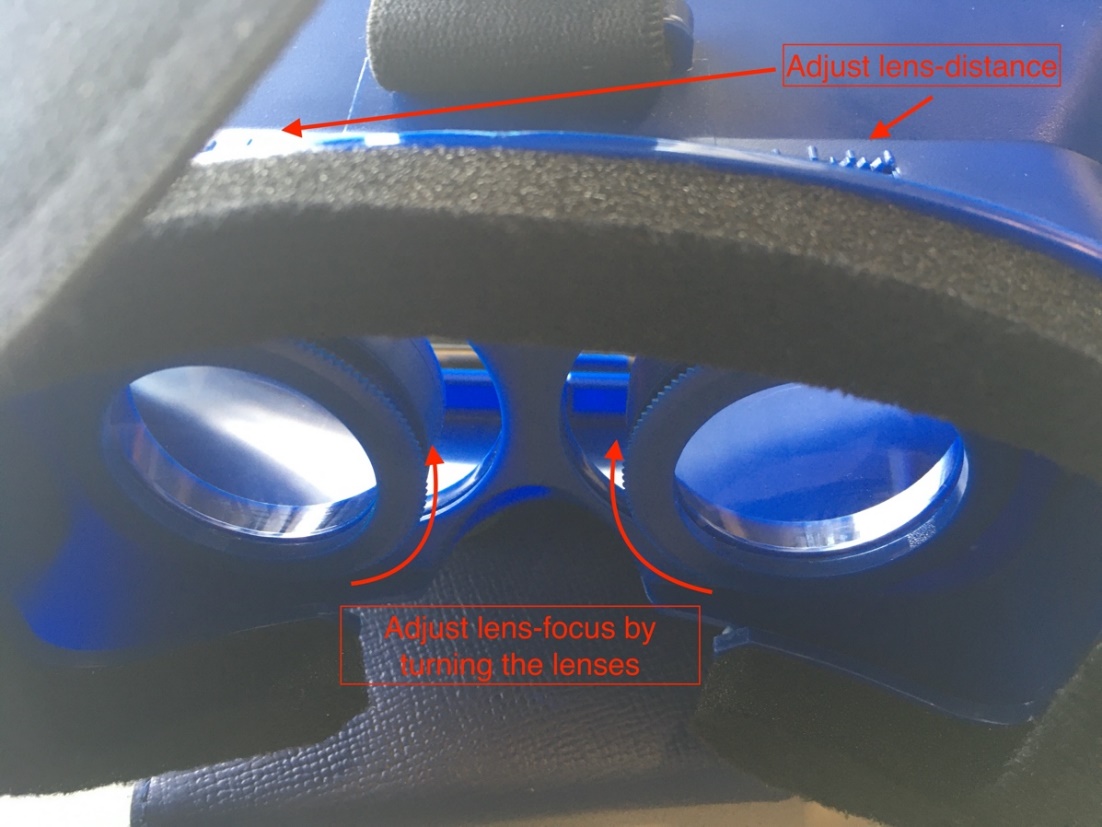
5. You can adjust the lens-distance and focus.

6.

Finally, plug in ear-/headphones and watch the show! For more comfort, you can place a soft piece of fabric between your nose and the goggles.


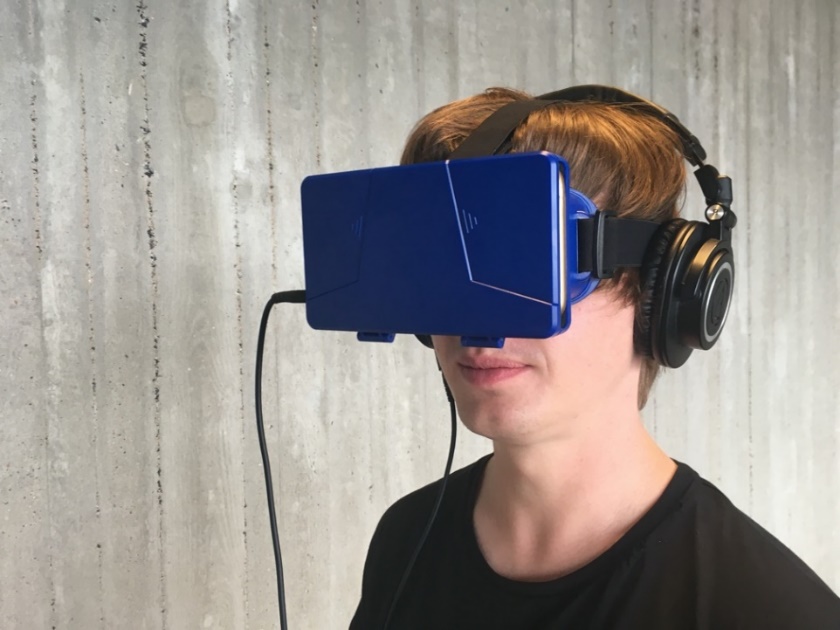


### Transcript introduction and explanation during concert

Each line was first delivered in Dutch, and then repeated in English, to accommodate for the varying languages of the audience members.

Host [01:40]: Welkom iedereen bij het tweede concert in onze reeks IPEM’s Experimental Sessions. Vandaag hebben we weer een divers publiek, dus ik zal de presentatie eerst even in het Nederlands doen en dan in het Engels. Dus ik ga eerst even de Engelse kijkers verwelkomen. Welcome everybody who is watching us today, at the second concert of our series IPEM’s Experimental Sessions. Again, today we have a diverse public – audience – and yeah, I will do the introduction of our musicians today, first in Dutch and then I’ll come back to you in English. So, vandaag beginnen we deze – dit concert – met Nemø. We hebben drie muzikanten van het Nemø ensemble en zij beschrijven hun muziek als mysterieuze melancholische rivieren die uitmonden in een klankenzee. Het wordt een improvisatie, maar verwacht in ieder geval een flirterig samenspel tussen de muzikanten. En ja, geniet ervan zou ik zeggen. Then in English, I’ll come back to you. Today we have the musicians of Nemø, we have three musicians from the Nemø ensemble. They describe their music as mysterious melancholic rivers that emerge into a sea of sounds. It will be an improvisation, but you can expect that there will be flirtatious interplay between the musicians in their program called FLIRT. So please give a warm welcome, geef alstublieft een warm welkom aan, Ben Bertrand, Wim Pelgrims, en Elisabeth Klinck. Hier is Nemø.

*Host* [27:12]: Dat was het dan helaas voor vanavond. Ik wil de muzikanten heel erg bedanken voor het spelen. So thank you very much for playing for us. En voor jullie thuis, ga alsjeblieft naar die email en vul de vragenlijst in. En ik hoop jullie weer te zien bij het volgende concert. So, for the audience at home, please go to your emails and fill out the questionnaire and I hope to see you at one of the other concerts. Ok, thank you, and bye.

## Concert 3

### Communication via email before concert: English

#### Group: YouTube

Here you can find the link to the livestream of the concert by Shalan Alhamwy & Jonas De Rave:

<https://youtu.be/RZbyA6a_rPk>

​Do not share this link with others. **Please watch the livestream on a laptop or desktop screen and listen via head-/earphones**, if you can.

After the concert please answer the questions of the following questionnaire:

Click here for questionnaire [hyperlink].

Your **personal ID code is […]**.

**MusicLab App (Optional participation)**

Attached you can find instructions for an app. This is an *optional feature* to our research. The app is developed by our partner at the university of Oslo and it would give us information about your movement during the concert(s). You can read through the attachment and decide whether you want to do this.

iOS: <https://apps.apple.com/us/app/musiclab/id1512077801>

Android: <https://play.google.com/store/apps/details?id=no.uio.mobileapps.musiclab&hl=en>​

Enjoy!

Kelsey​

#### Group: Zoom

Here you can find the link to the livestream of the concert by Shalan Alhamwy & Jonas De Rave:

[link]

​Do not share this link with others. **Please watch the livestream on a laptop or desktop screen and listen via head-/earphones**, if you can.

You indicated you were already familiar with Zoom. During this concert we ask you to **join the meeting with audio and video**, but please **mute your mic**. You will NOT be recorded. Comments might be anonymously exported. You can find more information attached.

After the concert please answer the questions of the following questionnaire:

Click here for questionnaire [hyperlink].

**Your personal ID code is […]**.

**MusicLab App (Optional participation)**

Collaborators at the University of Oslo have developed an exciting mobile phone application that measures movement in response to music. We would love for you to participate. Please find the instructions on how to use the app in the attachment. Download the app here:

iOS: <https://apps.apple.com/us/app/musiclab/id1512077801>

Android: <https://play.google.com/store/apps/details?id=no.uio.mobileapps.musiclab&hl=en>​

Enjoy!

Kelsey​

### Communication via email before concert: Dutch

#### Group: YouTube

Bij deze ontvangt u de link naar het livestream concert van Shalan Alhamwy & Jonas De Rave:

<https://youtu.be/RZbyA6a_rPk>

Deel deze link niet met anderen. **Gelieve het livestream concert via een laptop of computer scherm te bekijken en te luisteren via oordopjes/hoofdtelefoon**, als dit mogelijk is.

Na het concert vragen we u om een vragenlijst in te vullen. Deze is te vinden via de volgende link: Klik hier voor de vragenlijst [hyperlink].

**Uw persoonlijke ID code is […]**.

**MusicLab App (Optioneel)**

In de bijlage bevinden zich instructies voor een app. Dit is een *optionele mogelijkheid* van ons onderzoek. De app is ontwikkeld door onze partners aan de universiteit van Oslo en zou uw bewegingen kunnen registreren tijdens het concert. De bijlage kunt u alvast doorlezen om te beslissen of u dit wilt.

iOS: <https://apps.apple.com/us/app/musiclab/id1512077801>

Android: <https://play.google.com/store/apps/details?id=no.uio.mobileapps.musiclab&hl=en>​

Veel plezier!

Kelsey

#### Group: Zoom

Bij deze ontvangt u de link naar het livestream concert van Shalan Alhamwy & Jonas De Rave:

[link]

Deel deze link niet met anderen. **Gelieve het livestream concert via een laptop of computer scherm te bekijken en te luisteren via oordopjes/hoofdtelefoon**, als dit mogelijk is.

U heeft aangegeven al bekend te zijn met Zoom.Tijdens dit concert vragen we je de meeting in te gaan via **‘join with audio**’ en uw **camera aan te zetten**. Gelieve uw **microfoon te muten**. U zult tijdens het concert NIET opgenomen worden. Eventuele comments in de chat kunnen wel anoniem geëxporteerd worden. Meer uitleg vindt u in de bijlage.

Na het concert vragen we u om een vragenlijst in te vullen. Deze is te vinden via de volgende link: Klik hier voor vragenlijst [hyperlink].

**Uw persoonlijke ID code is […]**.

**MusicLab App (Optioneel)**

In de bijlage bevinden zich instructies voor een app. Dit is een *optionele mogelijkheid* van ons onderzoek. De app is ontwikkeld door onze partners aan de universiteit van Oslo en zou uw bewegingen kunnen registreren tijdens het concert. De bijlage kunt u alvast doorlezen om te beslissen of u dit wilt.

iOS: <https://apps.apple.com/us/app/musiclab/id1512077801>

Android: <https://play.google.com/store/apps/details?id=no.uio.mobileapps.musiclab&hl=en>​

Veel plezier!

Kelsey

### Instructions Zoom

**1.** Open the Zoom link: <https://uio.zoom.us/j/65135461439>

**2.** Open the Zoom app or click ‘join from your browser’


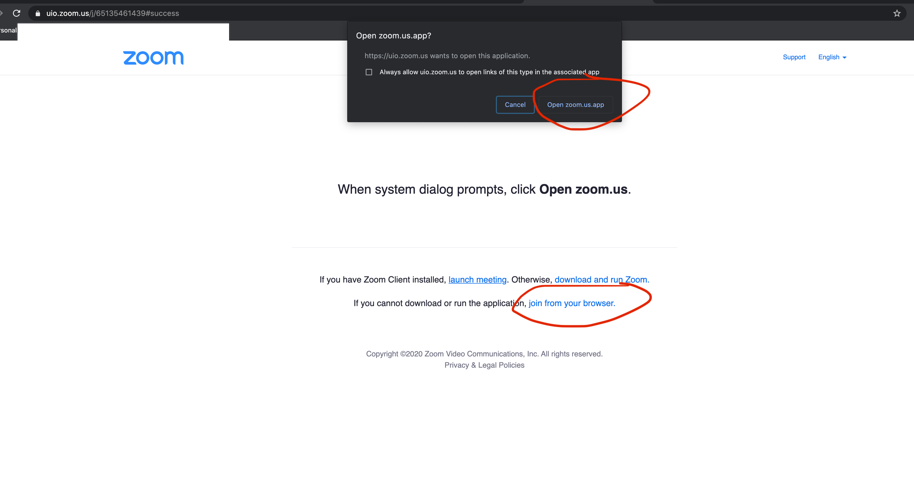


**3.** Click ‘Join with Video’


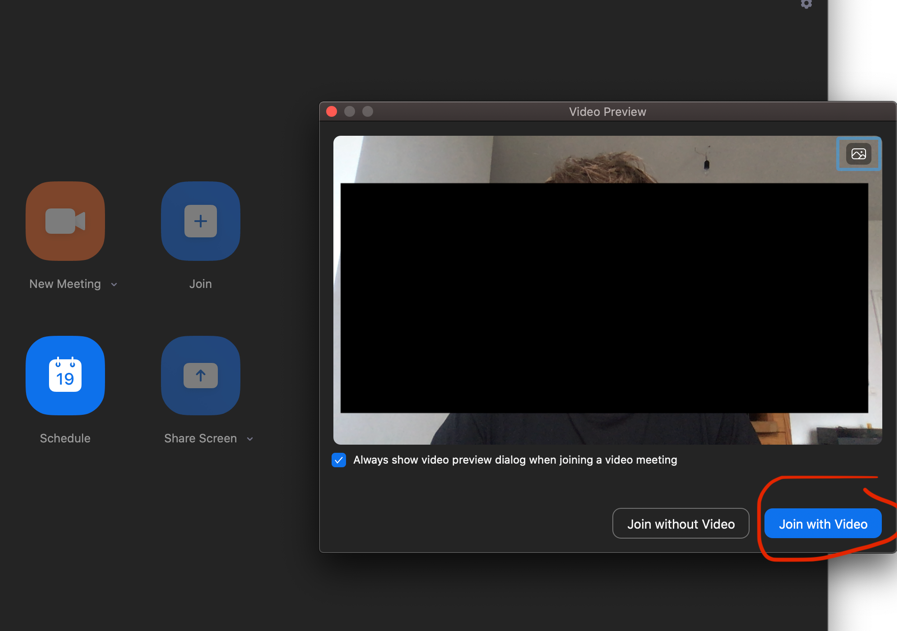


**4.** Click ‘Join with Computer Audio’


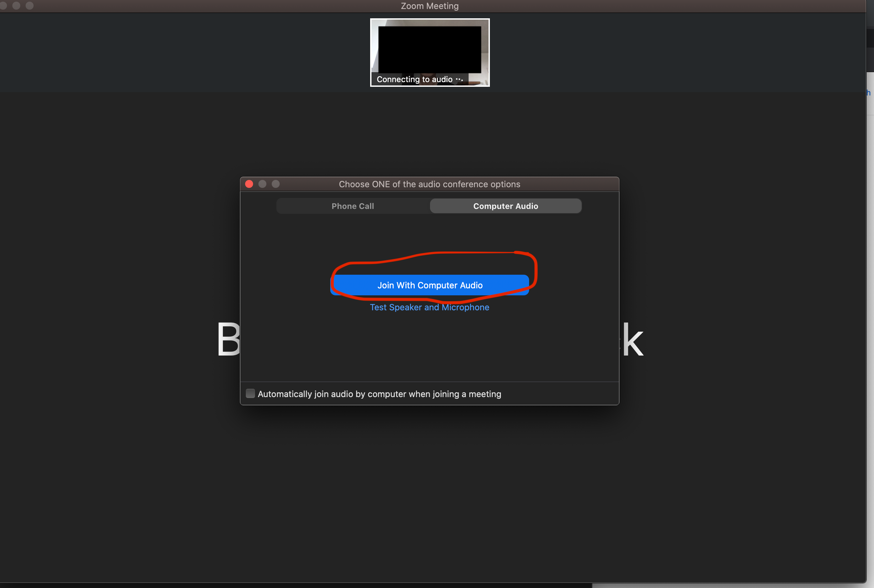


**5.** Make sure you are watching the concert in ‘Speaker View’. In green below you see the correct ‘Speaker View’ or button on which to select it, in red you see the wrong ‘Gallery View’ and where to select it

**
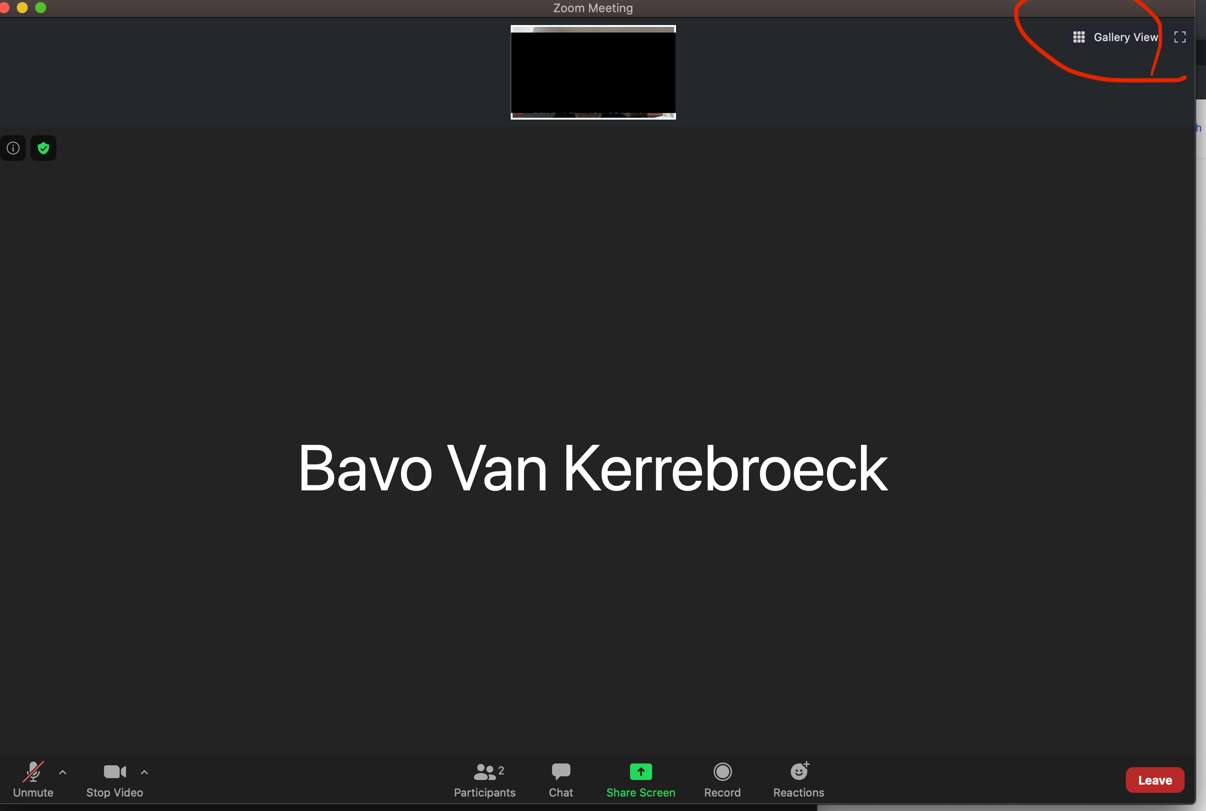
**


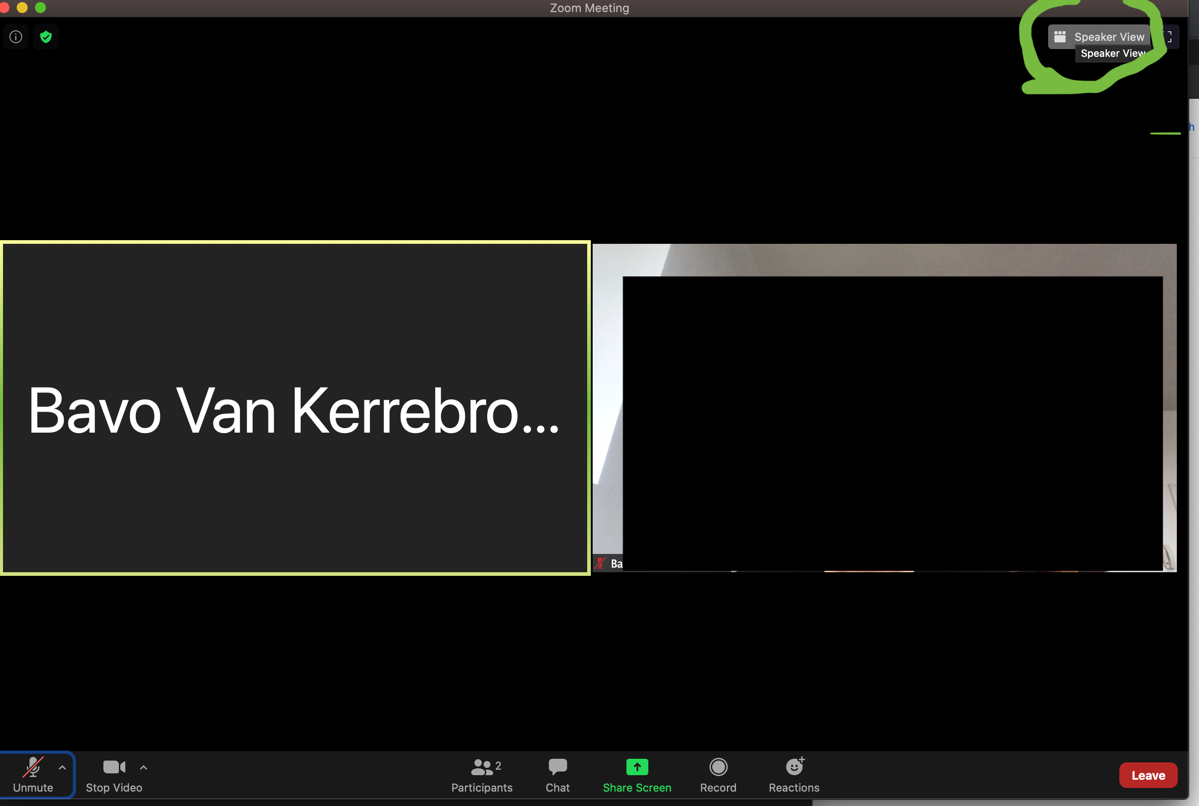


**6.** Keep your microphone muted at all times!

### Instructions MusicLabApp

### Transcript introduction and explanation during concert

Each line was first delivered in Dutch, and then repeated in English, to accommodate for the varying languages of the audience members.

*Host* [01:42]: Hello everyone, we’ll take five more minutes. We’re waiting for more audience to come in. We wachten nog even vijf minuten, we wachten nog op wat meer publiek wat hier nog binnen gaat komen. We zijn zo bij u terug.

*Host* [07:31]: Hallo iedereen en welkom bij de derde en laatste concert van IPEM’s Experimental Sessions. Ook vandaag hebben we weer een zeer divers publiek, dus ik zal het ook nog even in het Engels onze gasten verwelkomen. Hi everyone, welcome to the third and final concert of IPEM’s Experimental Sessions. Today, we have got once again, a global audience, so I’m gonna do this introduction in Dutch and in English.

*Host* [08:01]: Voordat ik de artiesten ga aankondigen, komt hier nog een kleine tutorial. Dit is voor de mensen hier in de zaal, of de mensen thuis die gebruik maken van de MusicLabApp. Mocht u daar gebruik van maken, pak dan uw telefoon erbij, en druk op het app icoontje. Als u de app opent geeft u toestemming en vervolgens vult u daar uw persoonlijke ID code in. Die persoonlijke ID code vindt u ook terug in de e-mail die u gisteren van ons heeft ontvangen. Vervolgens gaat u naar het tabje data en daar komt een pop-up tevoorschijn die vraagt of, die vraagt toestemming of de locatiegegevens gebruikt mogen worden, en ook daarop klikt u ‘ja’. Want die hebben wij dan nodig. Vervolgens drukt u op, of nee.. U zoekt eigenlijk een platte ondergrond, dat mag bijvoorbeeld een bureautafel zijn waar u achter zit, of de vloer. U legt uw telefoon daarop, u drukt op ‘start capture’ en u laat uw telefoon tien seconden liggen. Na die tien seconden drukt u op stop. Vervolgens pakt u de telefoon en legt u hem tegen uw borst aan. En ook daar drukt u op ‘start capture’ en probeert u in de tussentijd zo stil mogelijk te blijven staan. En na tien seconden drukt u weer op ‘stop’. Nu is uw telefoon gekalibreerd en klaar om gebruikt te worden. Nou als het concert zo meteen start, drukt u wederom op ‘start capture’. En zodra u de eerste noten hoort, beweegt u uw telefoon twee keer en kunt u het in uw borstzak steken, of houdt u het tegen uw borst aan. Nou mocht u nog vragen hebben over de app en hoe dat verder gebruikt moet worden kunt u gewoon een reactie achterlaten in de comment sectie op YouTube of Zoom, en onze moderators die zullen u verder helpen.

*Host* [09:55]: Alright, in English. For those of you that are here in the audience, or for the people watching at home that are using the MusicLabApp, here follows a short tutorial on how to use the app. So please grab your phone and open the app. First you have to give consent and afterwards you have to fill in your personal ID code. The personal ID code you can find in the email you received from us yesterday. Then you go to the app data, and there comes a pop-up that asks ‘can we use your geolocation?’ and please say ‘yes’, cause we need that information. Now we have to calibrate the app. So first off, you have to look for a flat surface, for example, a desk or the floor. So please put your phone on the flat surface, press ‘start capture’ and leave it on there for ten seconds. After those ten seconds, press ‘stop’. Then grab your phone and put it to your chest, press ‘start capture’ again, and try to stay still in the meanwhile cause you have to put it there for about ten seconds once again. And after those ten seconds you press ‘stop’ again. Now your phone is calibrated and ready to use. So when the concert starts in a couple of moments, you can press once again ‘start capture’, and when you hear the first music, you wave your phone twice and then you can put it in your chest pocket. If you have any further questions about the use of the app, don’t hesitate to ask us, you can leave a comment inside the comment section on YouTube and in Zoom.

*Host* [11:44]: Zo dan hebben wij dat gehad en gaan we door naar de introductie. Want vandaag ook hebben wij weer een leuk optreden voor u in petto. Allereerst hebben we Shalan Alhamwy. Shalan is violist en componist en is thuis in klassieke en Arabische muziek. En we verwelkomen Jonas De Rave, hij speelt accordeon en toetsen. Hij is thuis in jazzy worldgroove en klezmer. Vandaag geven zij twee een exclusief optreden voor jullie hier en voor de mensen thuis.

*Host* [12:11]: Now introducing today’s musicians. First we got Shalan Alhamwy, he is a violinist and composer. He studied classical and Arabic music and feels at home in both musical traditions. And we have got Jonas De Rave, he plays accordion and keys. His speciality is jazzy worldgroove and klezmer. Together Shalan and Jonas will give us an exclusive concert. So without further ado, please give a warm welcome for Shalan Alhamwy and Jonas de Rave. Een hartelijk applaus.

*Artist* *1- Shalan Alhamwy* [23:12]: Thank you very much. Good evening. This was ‘Oyfn veg shteyt a boym’. That was a Yiddish song, arranged by Jonas. And the second tune was ‘Ya loor’. An old Byzantian tune from the Syrian orthodox Christian liturgy, and arranged by me. And next we are going to play, a Syrian piece, a Syrian tune by a composer from the Syrian city Aleppo. It is a very traditional form. It is called Longa. It is not only in Syria, but it is also in Turkey, in the Middle East, a very very common musical form. And it is known to be fast and virtuosic. The name is Longa.

*Artist 2 – Jonas De Rave* [27:57]: We gaan verder zetten met een liedje dat ik zelf geschreven heb. Het gaat over de Opaalkust, net over de Franse grens. (Translation: We will continue with a song I have written myself. It is about the Opal Coast, just over the France border.)

*Artist 1* [32:00]: Thank you very much. Now we are going to play the last tune for this evening. It is a piece I wrote four years ago. Inspired by my city and I named it ‘Old Homs’, and old Homs – the historical part of my city Homs in Syria. You are going to hear that it’s really an Arabic tune, Arabic music, Arabic character, but also approached with a modern way.

*Host [35:30]:* Dankjewel Shalan en dankjewel Jonas. Jullie ook dankjewel voor de mensen die aanwezig zijn en voor de kijkers thuis, ook geweldig dat jullie hebben gekeken. Voor iedereen geldt, vergeet vooral niet de vragenlijst in te vullen. Thank you everyone here in the audience and for everyone at home for watching, and please don’t forget to fill in the questionnaire. This is it. Goodbye. Dank-jullie-wel.

# Supplementary Material S3: Questionnaires in English

## Registration

*Registration IPEM livestream concerts*

Thank you for participating in our experimental concert sessions!

Before and after the concert we want to ask you some questions. With the current form we will ask you to provide some general information, such as demographic characteristics, concert behavior, and a small set of questions about the current pandemic. At the end you will also provide an e-mail address which we can use to send you a link to the livestream and give further information.

From then on you will be provided with a personal ID so we can ensure your information will be totally anonymous. You will use this code when filling out the questionnaire after the concert. This questionnaire will ask about your experience of the concert.

If you register for the concert of Shalan Alhamwy & Jonas De Rave we will send you information about an optional feature: an app with which we would be able to register your movement during the concert. You are completely free to decide whether you want to use this feature or not.

After the concert your e-mail address will be deleted by the researcher in charge.

For any questions or remarks feel free to contact us at ipem.sessions@gmail.com

Thanks again,

Kelsey Onderdijk

Section 1

*Informed consent*

If you decide to participate in our study, we kindly ask you to state your approval regarding the following:

I declare that I wish to participate in a survey from Ghent University, and ...

(1) I have read the information on the nature of the experiment, the data that will be gathered, and I got the opportunity to ask additional questions (contact info on previous and last page)

(2) I am participating completely voluntarily in this study

(3) I give permission to process, preserve, and report my results anonymously

(4) I am aware my anonymous results can be shared between researchers internationally, possibly through an online repository

(5) I am aware that I can discontinue my participation at any time

(6) I realize that not participating or discontinuing to participate will in no way influence my evaluation and/or any tutoring I receive from Ghent University

(7) I can get a summary of the research findings, on request (contact info on previous and last page)

1. Have you read the above statements and are you 18 years or older?

- Yes, I am 18 years or older and hereby consent to participate in this study according to the statements provided above.

Section 2

*General information*

2. Which concert(s) do you wish to attend?

- Giusy Caruso - 21st July - 20:00 REGISTRATION FOR THIS CONCERT IS CLOSED
- Nemø - 22nd July - 19:00 REGISTRATION FOR THIS CONCERT IS NOW CLOSED
- Shalan Alhamwy & Jonas De Rave - 27th July - 20:00

3. Would you be interested in watching the livestream of a concert while being physically present in the same room with other people?

*This would be organized from our research lab at De Krook (miriam makebaplein 1, Gent). The usual safety precautions would be taken (social distancing, desinfect hands, mouth masks).*

- Yes
- No

4.Please indicate which platforms you are familiar with:

- YouTube (Live)
- Twitch
- Zoom
- None of the above

5. Do you have a smartphone?

- Yes
- No *(redirected to question 7)*

6. Is your smartphone bigger than 150 mm or 5.9 inch?

- Yes
- No

7. What is your age?

8. What is your gender?

- Woman
- Man
- Prefer not to say
- Other…

9. Do you live in Ghent?

- Yes *(redirected to question 11)*
- No

10. What is your country of residence?

11. Do you have a hearing impairment?

- Yes
- No *(redirected to question 13)*

12.Is this hearing impairment corrected? (e.g., hearing aid)

- Yes
- No

13.Do you have a visual impairment? (e.g., wearing glasses, lenses)

- Yes
- No *(redirected to question 15)*

14.Is this visual impairment corrected? (multiple answers possible)

- Yes, I wear glasses
- Yes, I wear lenses
- No
- Other…

15. How often did you feel the following since the beginning of the lockdown measures (on a scale from 1-5)?

1 Hardly ever 2 3 Some of the time 4 5 Often

Loneliness

Lack of companionship

Isolation from others

Anxiety

16. Indicate to what extent the following statements apply to you (on a scale from 1-5):

1 Almost never 2 3 4 5 Often

I worry about other people during the current pandemic

I worry about my own well-being during the current pandemic

17. On average, how many hours per day do you actually spend listening to music, either while doing something else or as your main activity?

- 0
- 1-2
- 3-4
- 5-8
- 9 or more

18. What is your usual level of attention or involvement when you listen to music (on a scale from 1-5)?

- 1 Background only
- 2
- 3
- 4
- 5 Total concentration

19. How often did you attend concerts before there were any lockdown restrictions?

- Never
- Less than once a month
- Once or twice a month
- About once a week
- More than once a week

20. How often do you attend concerts now that you are allowed to attend with precautions?

- Never
- Less than once a month
- Once or twice a month
- About once a week
- More than once a week

21. How often have you watched livestreamed concerts in the past month, while watching in real-time?

- Never
- Less than once a month
- Once or twice a month
- About once a week
- More than once a week

22. How often have you watched livestreamed concerts in the past month, after it had been recorded?

- Never
- Less than once a month
- Once or twice a month
- About once a week
- More than once a week

23. How much do you miss attending concerts while physically present (on a scale from 1-5)?

- 1 Not at all
- 2
- 3
- 4
- 5 Very much

24. To what extent do the following statements apply to you (on a scale from 1-5)?

*Please read carefully.*

1 Does not describe me well 2 3 4 5 Describes me very well

I often have tender, concerned feelings for people less fortunate than me.

Other people’s misfortunes do not usually disturb me a great deal.

When I see someone being taken advantage of, I feel kind of protective toward them.

Sometimes I don’t feel sorry for other people when they are having problems.

When I see someone being treated unfairly, I sometimes don’t feel very much pity for them.

I would describe myself as a pretty soft‐hearted person.

I am often quite touched by things that I see happen.

Section 3

That is it for now!

If you have any remaining questions or comments please contact us at: ipem.sessions@gmail.com

25. Please provide an e-mail address to which we can send the link to the livestream.

*As mentioned on the first page, we will only use your e-mail address for this organizational purpose. It will be removed after the concert has taken place.*

Keep an eye on your spam folder for the link!

## Concert 1

### Concert 1: voting

English and Dutch speakers received the same form.

*Stemmen/Vote*

1. Wat is je persoonlijke ID code? / What is your personal ID code?

Section 2

2. Welk stuk wil je graag horen? / Which piece would you like to hear?

- Danza de la moza donosa (melodisch/melodic)
- Danza del gaucho matrero (ritmisch/rhythmic)

### Concert 1: experience

*Concert experience - Giusy Caruso*

Please answer the following questions regarding your experience of the concert.

Section 1

1 What is your personal ID code?

Section 2

2 What type of screen did you use for watching the livestream?

- Phone screen
- Tablet
- Laptop
- Desktop screen
- (Big) TV screen
- Large projection
- VR headset
- Other…

3 What type of audio did you use for listening to the concert?

- Simple headphones/earplugs
- High-quality headphones/earplugs
- Built-in speakers
- External speakers
- High-quality speakers
- Other…

4 How did you experience the video quality (on a scale from 1-5)?

- 1 Very bad
- 2
- 3
- 4
- 5 Excellent

5 How did you experience the sound quality (on a scale from 1-5)?

- 1 Very bad
- 2
- 3
- 4
- 5 Excellent

6 Do you know the artist personally?

- Yes
- No

7 Before the concert, to what extent were you already a fan of the artist (on a scale from 1-5)?

- 1 I did not know the (music of the) artist
- 2
- 3
- 4
- 5 I am a huge fan

8 How long did you watch the concert (in minutes)?

*If you watched the whole concert you can write 'whole'*

9 What was your level of attention when you were watching the concert (on a scale from 1-5)?

- 1 Background only
- 2
- 3
- 4
- 5 Total concentration

10 How often did you walk away from the concert?

11 Did you have any (internet)connectivity issues during the concert? If yes, please explain below. If no, you can just answer 'no'.

12 Were there other people in the same room watching/listening with you?

- Yes
- No *(redirected to question 14)*

13 How many?

Section 3

14 To what extent did you feel connected with the ARTIST during the concert (on a scale from 1-5)?

- 1 Not at all
- 2
- 3
- 4
- 5 Very much

15 Looking at the figure below, indicate which of these circles describes YOU (self) and the ARTIST (other) best during the concert experience?


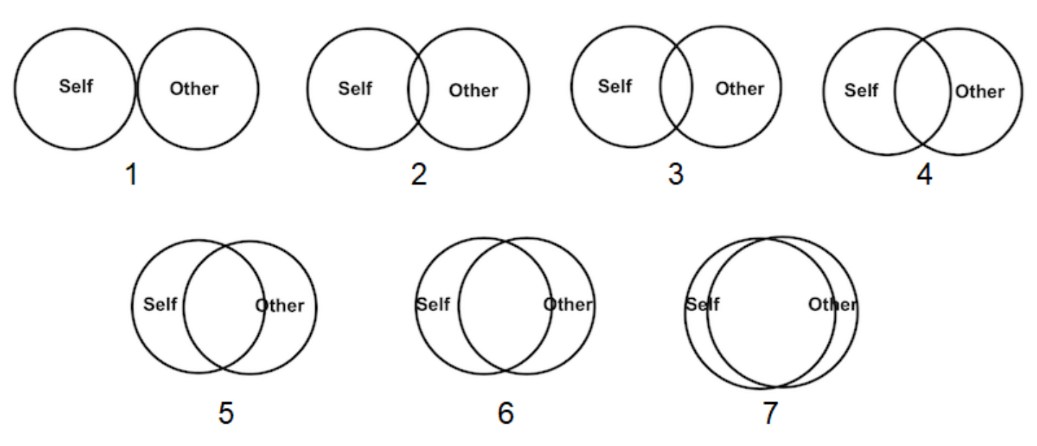


*If unsure, answer with your gut feeling.*

- 1
- 2
- 3
- 4
- 5
- 6
- 7

16 To what extent were you aware of other people in the (virtual) audience (on a scale from 1-5)?

*With the virtual audience we mean the people who watch the livestream online*

- 1 Not at all
- 2
- 3
- 4
- 5 Very much

17 To what extent did you feel as if you shared emotions with others in the (virtual) audience (on a scale from 1-5)?

- 1 Not at all
- 2
- 3
- 4
- 5 Very much

18 To what extent did you feel you were sharing the experience with others in the (virtual) audience?

- 1 Not at all
- 2
- 3
- 4
- 5 Very much

19 How connected did you feel with the (virtual) AUDIENCE during the concert (on a scale from 1-5)?

- 1 Not at all
- 2
- 3
- 4
- 5 Very much

20 Looking at the figure below, indicate which of these circles describes YOU (self) and the (virtual) AUDIENCE (other) best during the concert experience?


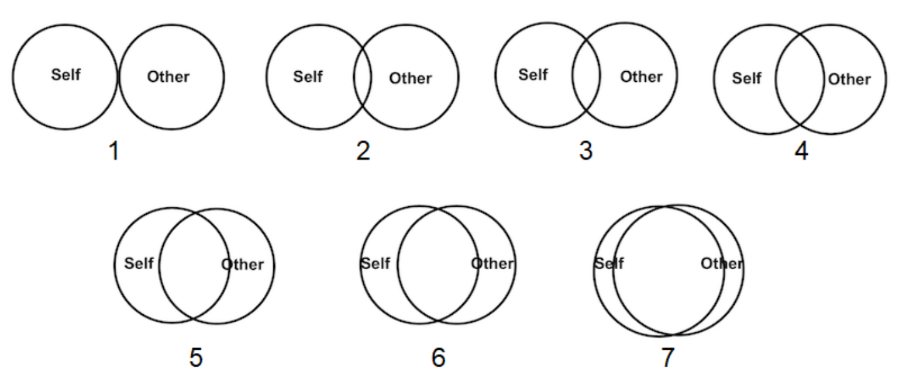


*If unsure, answer with your gut feeling.*

- 1
- 2
- 3
- 4
- 5
- 6
- 7

21 Did you know any other members of the (virtual) audience?

- Yes
- No / not that I know of

Section 4

22 Feelings of agency can be described as having a subjective feeling of control. In other words, agency is feeling a general sense of control over what you are doing or over the situation you are in.

Given this definition, to what extent did you feel you had agency over your concert experience (on a scale from 1-5)?

- 1 Not at all
- 2
- 3
- 4
- 5 Very much

23 When two or more people partake in something together, they can feel a sense of shared agency over what they are doing or the situation they are in. When you feel this shared agency, you feel as if you are doing something together (your experience is the product of something done together). When you do not feel shared agency, you feel as if others did not have any control over your experience, and that you acted completely independently.

Given this definition, to what extent did you feel shared agency over your concert experience (on a scale from 1-5)?

- 1 No shared control
- 2
- 3
- 4
- 5 Complete shared control

24 Were you allowed to vote for a last piece?

- Yes *(redirected to question 26)*
- No

25 If you would have been allowed to vote, which piece would you have chosen?

- The melodic piece: Danza de la moza donosa
- The rhythmic piece: Danza del gaucho matrero
- Don't know/ no preference at all

26 Did you already know one or both of the pieces that were voted on? (multiple answers possible)

- Yes, I already knew Danza de la moza donosa
- Yes, I already knew Danza del gaucho matrero
- No

27 Did you like the last piece that was played by the artist (on a scale from 1-5)?

- 1 Not at all
- 2
- 3
- 4
- 5 It was fantastic

28 Did the fact you were allowed / not allowed to vote influence your concert experience?

- Yes, I think it improved my experience
- Yes, I think it worsened my experience
- No, I don't think it was of influence
- I do not know

Section 5

29 Lastly we have some statements of which we would like to know if you agree with them or not.

1 Do not agree at all 2 3 4 5 Agree completely

While I was at the streamed concert, I had a sense of “being there” with the performers and audience members.

I was completely captivated by the concert.

I felt like I was in the presence of other people who were online in the streamed concert.

I felt that the other people in the streamed concert were aware of my presence.

The people in the streamed concert appeared to be engaged and active to me.

My experience in the streamed concert seemed similar to my experiences in a real concert.

During the streamed concert there were times where the computer interface seemed to disappear, and I felt like I was actually at the concert.

During the concert I felt involved with the concert.

This way of attending concerts fulfilled my expectations.

This way of attending concerts gave me the same fulfillment as physically attending concerts as before the pandemic.

The concert (temporarily) made me forget my worries surrounding covid19.

I completely forgot I was partaking in an experiment.

30 To what extent did you feel the following more or less after attending the concert?

A lot less Less Unchanged More A lot more

Loneliness

Lack of companionship

Isolation from others

Anxiety

Section 6

Thank you!

Thank you very much for participating in our experimental sessions.

If you have any remaining questions or comments, or would wish to be informed about the results of the study, please leave them here or get in contact via ipem.sessions@gmail.com

Do not forget to press send!

31 Comments (optional)

## Concert 2

*Concert experience - Nemø*

Please answer the following questions regarding your experience of the concert.

Section 1

1 What is your personal ID code?

Section 2

2 What type of screen did you use for watching the livestream?

Phone screen

- VR headset with phone
- Tablet *(redirected to question 4)*
- Laptop *(redirected to question 4)*
- Desktop screen *(redirected to question 4)*
- (Big) TV screen *(redirected to question 4)*
- Large projection *(redirected to question 4)*
- Other… *(redirected to question 4)*

3 Did you ever try a VR headset before? (multiple possible answers)

- Yes, in my free time
- Yes, through the videos mentioned in the instructions
- No, this was the first time
- Other…

4 What type of audio did you use for listening to the concert?

- Simple headphones/earplugs
- High-quality headphones/earplugs
- Built-in speakers
- External speakers
- High-quality speakers
- Other…

5 How did you experience the video quality (on a scale from 1-5)?

- 1 Very bad
- 2
- 3
- 4
- 5 Excellent

6 How did you experience the sound quality (on a scale from 1-5)?

- 1 Very bad
- 2
- 3
- 4
- 5 Excellent

7 Do you know the artists personally?

- Yes
- No

8 Before the concert, to what extent were you already a fan of the artists (on a scale from 1-5)?

- 1 I did not know the (music of the) artist
- 2
- 3
- 4
- 5 I am a huge fan

9 How long did you watch the concert (in minutes)?

*If you watched the whole concert you can write 'whole'*

10 What was your level of attention when you were watching the concert (on a scale from 1-5)?

- 1 Background only
- 2
- 3
- 4
- 5 Total concentration

11 How often did you walk away from the concert?

12 Did you have any (internet)connectivity issues during the concert? If yes, please explain below. If no, you can just answer 'no'.

13 Were there other people in the same room watching/listening with you?

- Yes
- No *(redirected to question 15)*

14 How many?

15 Did you like the music that was played by the artists (on a scale from 1-5)?

- 1 Not at all
- 2
- 3
- 4
- 5 It was fantastic

Section 3

16 To what extent did you feel connected with the ARTISTS during the concert (on a scale from 1-5)?

- 1 Not at all
- 2
- 3
- 4
- 5 Very much

17 Looking at the figure below, indicate which of these circles describes YOU (self) and the ARTISTS (other) best during the concert experience?


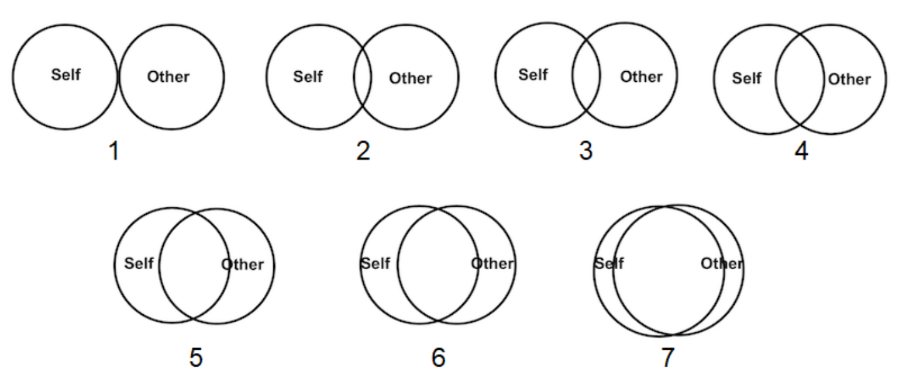


*If unsure, answer with your gut feeling.*

- 1
- 2
- 3
- 4
- 5
- 6
- 7

18 To what extent were you aware of other people in the (virtual) audience (on a scale from 1-5)?

*With the virtual audience we mean the people who watch the livestream online*

- 1 Not at all
- 2
- 3
- 4
- 5 Very much

19 To what extent did you feel as if you shared emotions with others in the (virtual) audience (on a scale from 1-5)?

- 1 Not at all
- 2
- 3
- 4
- 5 Very much

20 To what extent did you feel you were sharing the experience with others in the (virtual) audience?

- 1 Not at all
- 2
- 3
- 4
- 5 Very much

21 How connected did you feel with the (virtual) AUDIENCE during the concert (on a scale from 1-5)?

- 1 Not at all
- 2
- 3
- 4
- 5 Very much

22 Looking at the figure below, indicate which of these circles describes YOU (self) and the (virtual) AUDIENCE (other) best during the concert experience?


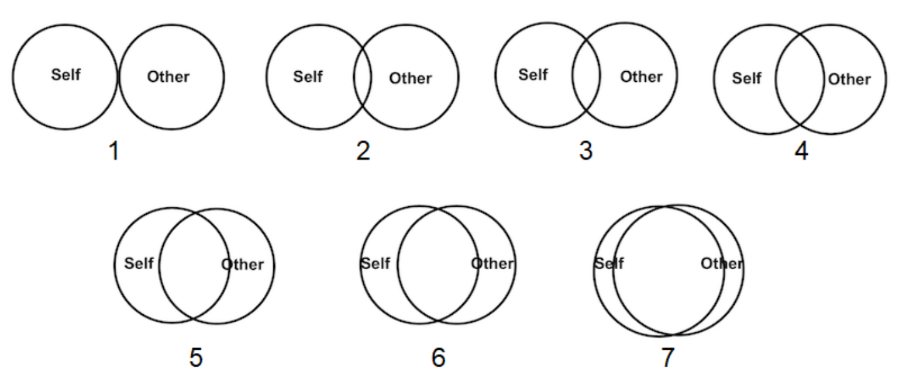


*If unsure, answer with your gut feeling.*

- 1
- 2
- 3
- 4
- 5
- 6
- 7

23 Did you know any other members of the (virtual) audience?

- Yes
- No / not that I know of

Section 4

24 Please indicate to what extent the following statements apply to your experience of the concert:

*With virtual world we mean the online (virtual) concert.*

1 Fully disagree 2 3 4 5 6 7 Fully agree

Somehow I felt that the virtual world surrounded me.

I felt like I was just perceiving pictures.

I had a sense of acting in the virtual space, rather than operating something from outside.

I felt present in the virtual space.

I was not aware of my real environment.

I still paid attention to the real environment.

I was completely captivated by the virtual world.

The virtual world seemed more realistic than the real world.

25 In the computer generated world I had a sense of "being there"

1 Not at all 2 3 4 5 6 7 Very much

26 I did not feel present in the virtual space.

1 Did not feel present 2 3 4 5 6 7 Felt present

27 How aware were you of the real world surrounding while navigating in the virtual world? (i.e. sounds, room temperature, other people, etc.)?

1 Extremely aware 2 3 4 Moderately aware 5 6 7 Not aware at all

28How real did the virtual world seem to you?

1 Completely real 2 3 4 5 6 7 Not real at all

29How much did your experience in the virtual environment seem consistent with your real world experience ?

1 Not consistent 2 3 4 Moderately consistent 5 6 7 Very consistent

30 How real did the virtual world seem to you?

1 About as real as an imagined world 2 3 4 5 6 7 Indistinguishable from the real world

Section 5

31 Feelings of agency can be described as having a subjective feeling of control. In other words, agency is feeling a general sense of control over what you are doing or over the situation you are in.

Given this definition, to what extent did you feel you had agency over your concert experience (on a scale from 1-5)?

- 1 Not at all
- 2
- 3
- 4
- 5 Very much

32 When two or more people partake in something together, they can feel a sense of shared agency over what they are doing or the situation they are in. When you feel this shared agency, you feel as if you are doing something together (your experience is the product of something done together). When you do not feel shared agency, you feel as if others did not have any control over your experience, and that you acted completely independently.

Given this definition, to what extent did you feel shared agency over your concert experience (on a scale from 1-5)?

- 1 No shared control
- 2
- 3
- 4
- 5 Complete shared control

Section 6

33 Lastly we have some statements of which we would like to know if you agree with them or not.

1 Do not agree at all 2 3 4 5 Agree completely

While I was at the streamed concert, I had a sense of “being there” with the performers and audience members.

I was completely captivated by the concert.

I felt like I was in the presence of other people who were online in the streamed concert.

I felt that the other people in the streamed concert were aware of my presence.

The people in the streamed concert appeared to be engaged and active to me.

My experience in the streamed concert seemed similar to my experiences in a real concert.

During the streamed concert there were times where the computer interface seemed to disappear, and I felt like I was actually at the concert.

During the concert I felt involved with the concert.

This way of attending concerts fulfilled my expectations.

This way of attending concerts gave me the same fulfillment as physically attending concerts as before the pandemic.

The concert (temporarily) made me forget my worries surrounding covid19.

I completely forgot I was partaking in an experiment.

34 To what extent did you feel the following more or less after attending the concert?

A lot less Less Unchanged More A lot more

Loneliness

Lack of companionship

Isolation from others

Anxiety

Section 7

Thank you!

Thank you very much for participating in our experimental sessions.

If you have any remaining questions or comments, or would wish to be informed about the results of the study, please leave them here or get in contact via ipem.sessions@gmail.com

Do not forget to press send!

35 Comments (optional)

## Concert 3

*Concert experience - Shalan Alhamwy & Jonas De Rave*

Please answer the following questions regarding your experience of the concert.

Section 1

1 What is your personal ID code?

Section 2

2 How did you attend the concert?

- Physical presence *(redirected to question 7)*
- Virtual presence

3 Did you have any (internet) connectivity issues during the concert? If yes, please explain below. If no, you can just answer 'no'.

4 Did you use the chat function or other ways of communicating your experience (e.g., reactions)?

- Yes
- No

5 Were there other people in the same room watching/listening with you?

- Yes
- No *(redirected to question 8)*

6 How many?

7 You were physically present at:

- Watching the livestream on a big screen in group *(redirected to question 10)*
- Attending the concert itself in group *(redirected to question 11)*

8 What type of screen did you use for watching the livestream?

- Phone screen
- Tablet
- Laptop
- Desktop screen
- (Big) TV screen
- Large projection
- VR headset
- Other…

9 What type of audio did you use for listening to the concert?

- Simple headphones/earplugs
- High-quality headphones/earplugs
- Built-in speakers
- External speakers
- High-quality speakers
- Other…

10 How did you experience the video quality (on a scale from 1-5)?

- 1 Very bad
- 2
- 3
- 4
- 5 Excellent

11 How did you experience the sound quality (on a scale from 1-5)?

- 1 Very bad
- 2
- 3
- 4
- 5 Excellent

12 Do you know (one of) the artist(s) personally?

- Yes
- No

13 Before the concert, to what extent were you already a fan of the artist(s) (on a scale from 1-5)?

- 1 I did not know the (music of the) artist
- 2
- 3
- 4
- 5 I am a huge fan

14 How long did you watch the concert (in minutes)?

*If you watched the whole concert you can write 'whole'*

15 What was your level of attention when you were watching the concert (on a scale from 1-5)?

- 1 Background only
- 2
- 3
- 4
- 5 Total concentration

16 How often did you walk away from the concert?

17 Did you like the music that was played by the artists (on a scale from 1-5)?

- 1 Not at all
- 2
- 3
- 4
- 5 It was fantastic

Section 3

18 To what extent did you feel connected with the ARTISTS during the concert (on a scale from 1-5)?

- 1 Not at all
- 2
- 3
- 4
- 5 Very much

19 Looking at the figure below, indicate which of these circles describes YOU (self) and the ARTISTS (other) best during the concert experience?


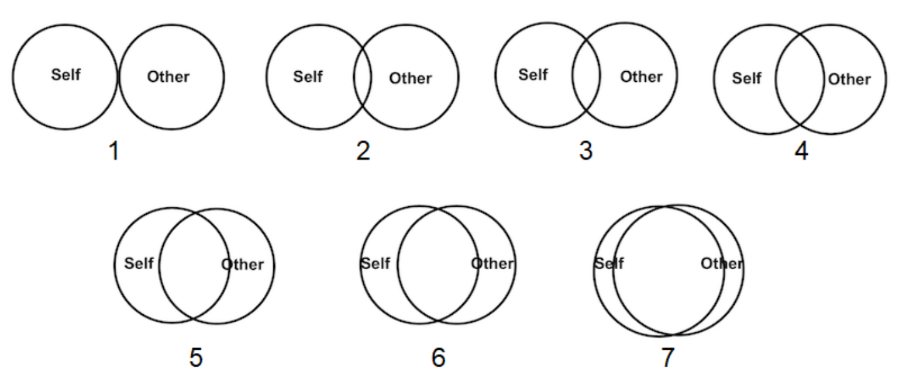


*If unsure, answer with your gut feeling.*

- 1
- 2
- 3
- 4
- 5
- 6
- 7

20 To what extent were you aware of other people in the audience (on a scale from 1-5)?

- 1 Not at all
- 2
- 3
- 4
- 5 Very much

21 To what extent did you feel as if you shared emotions with others in the audience (on a scale from 1-5)?

- 1 Not at all
- 2
- 3
- 4
- 5 Very much

22 To what extent did you feel you were sharing the experience with others in the audience?

- 1 Not at all
- 2
- 3
- 4
- 5 Very much

23 How connected did you feel with the AUDIENCE during the concert (on a scale from 1-5)?

- 1 Not at all
- 2
- 3
- 4
- 5 Very much

24 Looking at the figure below, indicate which of these circles describes YOU (self) and the AUDIENCE (other) best during the concert experience?


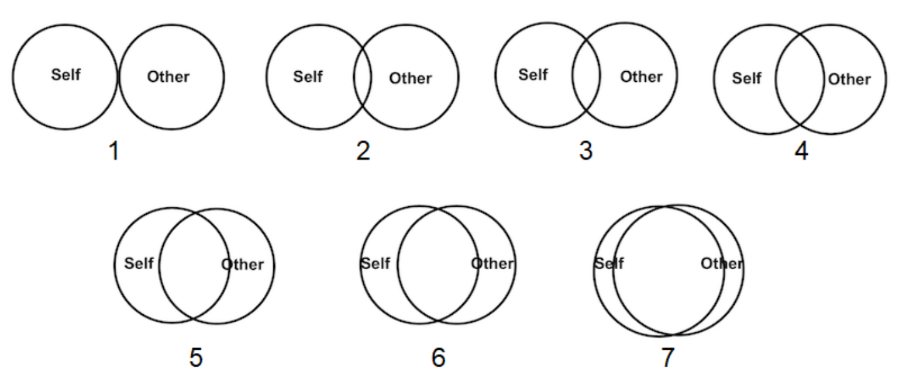


*If unsure, answer with your gut feeling.*

- 1
- 2
- 3
- 4
- 5
- 6
- 7

25 Did you know any other members of the audience?

- Yes, in the audience that was physically present
- Yes, in the audience that was virtually present
- Yes, in both the physically and virtually present audience
- No / not that I know of

Section 4

26 Feelings of agency can be described as having a subjective feeling of control. In other words, agency is feeling a general sense of control over what you are doing or over the situation you are in.

Given this definition, to what extent did you feel you had agency over your concert experience (on a scale from 1-5)?

- 1 Not at all
- 2
- 3
- 4
- 5 Very much

27 When two or more people partake in something together, they can feel a sense of shared agency over what they are doing or the situation they are in. When you feel this shared agency, you feel as if you are doing something together (your experience is the product of something done together). When you do not feel shared agency, you feel as if others did not have any control over your experience, and that you acted completely independently.

Given this definition, to what extent did you feel shared agency over your concert experience (on a scale from 1-5)?

- 1 No shared control
- 2
- 3
- 4
- 5 Complete shared control

Section 5

28 Lastly we have some statements of which we would like to know if you agree with them or not.

1 Do not agree at all 2 3 4 5 Agree completely

While I was at the streamed concert, I had a sense of “being there” with the performers and audience members.

I was completely captivated by the concert.

I felt like I was in the presence of other people who were online in the streamed concert.

I felt that the other people in the streamed concert were aware of my presence.

The people in the streamed concert appeared to be engaged and active to me.

My experience in the streamed concert seemed similar to my experiences in a real concert.

During the streamed concert there were times where the computer interface seemed to disappear, and I felt like I was actually at the concert.

During the concert I felt involved with the concert.

This way of attending concerts fulfilled my expectations.

This way of attending concerts gave me the same fulfillment as physically attending concerts as before the pandemic.

The concert (temporarily) made me forget my worries surrounding covid19.

I completely forgot I was partaking in an experiment.

29 To what extent did you feel the following more or less after attending the concert?

A lot less Less Unchanged More A lot more

Loneliness

Lack of companionship

Isolation from others

Anxiety

Section 6

Thank you!

Thank you very much for participating in our experimental sessions.

If you have any remaining questions or comments, or would wish to be informed about the results of the study, please leave them here or get in contact via ipem.sessions@gmail.com

Do not forget to press send!

30 Comments (optional)

# Supplementary Material S4: Questionnaires in Dutch

## Registration

*Registratie IPEM livestream concerten*

Hartelijk dank voor het meedoen aan onze experimentele concertsessies!

Voor en na het concert willen wij u enkele vragen stellen. Met dit huidige formulier vragen wij u om algemene informatie, zoals demografische kenmerken, uw concertgedrag, en een klein aantal vragen omtrent de huidige pandemie. Aan het einde vragen wij u om uw e-mailadres zodat wij u een link kunnen sturen naar de livestream en u verder informatie te verstrekken.

Daarna zal u een persoonlijke ID-code krijgen zodat wij kunnen garanderen dat alle informatie totaal anoniem verwerkt wordt. Deze code vult u in bij de vragenlijst na het concert. Deze vragenlijst zal uw ervaring van het concert nagaan.

Als u zich registreert voor het concert van Shalan Alhamwy & Jonas De Rave dan sturen wij u informatie over een bijkomende optie: een app waarmee wij uw beweging kunnen registreren tijdens het concert. U bent geheel vrij om te beslissen of u deze optie wilt gebruiken of niet.

Na het concert zal uw e-mailadres worden verwijderd door de verantwoordelijke onderzoeker.

Voor verdere vragen of opmerkingen contacteer ons via ipem.sessions@gmail.com

Nogmaals bedankt,

Kelsey Onderdijk

Section 1

*Geïnformeerde toestemming*

Als u besluit om aan dit onderzoek deel te nemen, vragen wij u vriendelijk om uw goedkeuring te geven met betrekking tot het volgende:

Ik verklaar dat ik wil deelnemen aan een onderzoek van de Universiteit Gent, en ...

(1) Ik heb de informatie gelezen over de aard van het experiment, de gegevens die zullen worden verzameld, en ik heb de mogelijkheid om aanvullende vragen te stellen (contactinformatie op de vorige en laatste pagina)

(2) Ik werk volledig vrijwillig mee aan dit experiment

(3) Ik geef toestemming om mijn resultaten volledig anoniem te verwerken, bewaren en rapporteren

(4) Ik realiseer me dat mijn anonieme resultaten gedeeld kunnen worden tussen onderzoekers internationaal, mogelijk via een online opslagplaats

(5) Ik realiseer me dat ik mijn deelname aan het experiment op elk moment kan stoppen.

(6) Ik realiseer me dat het niet deelnemen aan of stoppen met deelnemen op geen enkele manier van invloed is op mijn evaluatie en/of bijles die ik krijg van de Universiteit Gent

(7) Ik kan een overzicht krijgen van de onderzoeksresultaten op verzoek (contactinformatie op de vorige en laatste pagina)

1. Heeft u de bovenstaande verklaringen gelezen en bent u 18 jaar of ouder?

- Ja, Ik ben 18 jaar of ouder en geef hierbij toestemming om deel te nemen aan dit onderzoek volgens de bovenstaande verklaringen

Section 2

*Algemene informatie*

2. Welk(e) concert(en) wilt u bijwonen?

- Giusy Caruso - 21 juli - 20:00 REGISTRATIE VOOR DIT CONCERT IS GESLOTEN
- Nemø - 22 juli - 19:00 REGISTRATIE VOOR DIT CONCERT IS GESLOTEN
- Shalan Alhamwy & Jonas De Rave - 27 juli - 20:00

3.Zou u geïnteresseerd zijn in het bekijken van een livestream van een concert terwijl u fysiek aanwezig bent in een zelfde kamer met andere mensen?

*Dit zou georganiseerd worden vanuit ons onderzoekslab in De Krook (Miriam Makebaplein 1, Gent). De gebruikelijke veiligheidsmaatregelen worden in acht genomen (social distancing, het desinfecteren van de handen, mondmaskers).*

- Ja
- Nee

4. Geef aan met welke platforms u bekend bent:

- YouTube (Live)
- Twitch
- Zoom
- Geen van bovenstaande

5. Heeft u een smartphone?

- Ja
- Nee *(redirected to question 7)*

6. Is uw smartphone groter dan 150 mm of 5.9 inch?

- Ja
- Nee

7. Wat is uw leeftijd?

8.Wat is uw gender?

- Vrouw
- Man
- Zeg ik liever niet
- Other…

9.Woont u in Gent?

- Ja *(redirected to question 11)*
- Nee

10. In welk land woont u?

11. Heeft u gehoorproblemen?

- Ja
- Nee *(redirected to question 13)*

12.Zijn deze gehoorproblemen gecorrigeerd? (bijv. gehoorapparaat)

- Ja
- Nee

13.Heeft u een problemen met zicht? (bijv. bril, lenzen)

- Ja
- Nee *(redirected to question 15)*

14.Is dit probleem met zicht gecorrigeerd? (meerdere antwoorden mogelijk)

- Ja, ik draag een bril
- Ja, ik draag lenzen
- Nee
- Other…

15.Sinds het begin van de lockdown regelingen, hoe vaak voelde u het volgende (op een schaal van 1-5):

1 Bijna nooit 2 3 Soms 4 5 Vaak

Eenzaamheid

Gebrek aan gezelschap

Afzondering van anderen

Angstig

16. Geef aan in hoeverre de volgende statements op jou van toepassing zijn (op een schaal van 1-5):

1 Bijna nooit 2 3 4 5 Vaak

Ik maak mij erg zorgen om anderen tijdens de huidige pandemie

Ik maak mij erg zorgen om mijn eigen gesteldheid tijdens de huidige pandemie.

17. Hoeveel uur per dag besteedt u gemiddeld aan het luisteren naar muziek, zowel terwijl u iets anders doet of als uw hoofdactiviteit?

- 0
- 1-2
- 3-4
- 5-8
- 9 of meer

18. Wat is uw gebruikelijke aandachtsniveau of betrokkenheid als u naar muziek luistert (op een schaal van 1-5)?

- 1 Alleen achtergrond
- 2
- 3
- 4
- 5 Complete concentratie

19. Hoe vaak woonde u concerten bij voor de lockdown beperkingen?

- Nooit
- Minder dan een keer per maand
- Een of twee keer per maand
- Een keer per week
- Meer dan een keer per week

20. Hoe vaak gaat u naar een concert nu het weer is toegestaan om concerten te bezoeken met voorzorgsmaatregelen?

- Nooit
- Minder dan een keer per maand
- Een of twee keer per maand
- Een keer per week
- Meer dan een keer per week

21. Hoe vaak heeft u in de afgelopen maand een livestream-concert bekeken, terwijl u in realtime keek?

- Nooit
- Minder dan een keer per maand
- Een of twee keer per maand
- Een keer per week
- Meer dan een keer per week

22. Hoe vaak heeft u in de afgelopen maand een livestream-concert bekeken, nadat het was opgenomen?

- Nooit
- Minder dan een keer per maand
- Een of twee keer per maand
- Een keer per week
- Meer dan een keer per week

23. Hoe erg mis je het bezoeken van concerten in levende lijve? (op een schaal van 1-5)?

- 1 Totaal niet
- 2
- 3
- 4
- 5 Heel erg

24.Geef aan in hoeverre de volgende statements op u van toepassing zijn (op een schaal van 1-5):

*Gelieve zorgvuldig te lezen*

1 Beschrijft mij niet 2 3 4 5 Beschrijft mij goed

Ik heb vaak tedere, bezorgde gevoelens voor mensen die minder gelukkig zijn dan ik.

Soms heb ik niet veel medelijden met andere mensen wanneer ze problemen hebben.

Wanneer ik iemand zie waarvan wordt geprofiteerd, voel ik me nogal beschermend tegenover hen.

Andermans ongelukken verstoren me meestal niet veel.

Wanneer ik zie dat iemand unfair wordt behandeld, voel ik soms weinig medelijden met hen.

Ik ben vaak nogal geraakt door dingen die ik zie gebeuren.

Ik zou mijzelf beschrijven als een vrij teerhartig persoon.

Section 3

Dat was het voor nu!

Mocht u nog verdere vragen of opmerkingen hebben, contacteer ons dan via: ipem.sessions@gmail.com

25. Gelieve hier een e-mailadres op te geven waarnaar wij u een link mogen sturen van het livestream concert.

*Zoals aangegeven op de eerste pagina gebruiken wij uw e-mailadres alleen voor deze organisatorische doeleinden. Het zal worden verwijderd nadat het concert heeft plaatsgevonden.*

Houd uw spam folder eventueel in de gaten voor de link!

## Concert 1

### Concert 1: voting

English and Dutch speakers received the same form.

*Stemmen/Vote*

1. Wat is je persoonlijke ID code? / What is your personal ID code?

Section 2

2. Welk stuk wil je graag horen? / Which piece would you like to hear?

- Danza de la moza donosa (melodisch/melodic)
- Danza del gaucho matrero (ritmisch/rhythmic)

### Concert 1: experience

*Concertervaring - Giusy Caruso*

Geef antwoord op de volgende vragen over uw ervaringen van het concert.

Section 1

1Wat is uw persoonlijke ID-code?

Section 2

2 Op welk type scherm heeft u de livestream bekeken?

- Telefoonscherm
- Tablet
- Laptop
- Desktop
- (Grote) televisie
- Grote projectie
- VR headset
- Other…

3 Met welk type audio heeft u het concert beluisterd?

- Simpele koptelefoon/oordopjes
- Hoge kwaliteits koptelefoon/oordopjes
- Ingebouwde speakers
- Externe speakers
- Hoge kwaliteit speakers
- Other…

4 Hoe heeft u de videokwaliteit ervaren (op een schaal van 1-5)?

- 1 Heel slecht
- 2
- 3
- 4
- 5 Excellent

5 Hoe heeft u de geluidskwaliteit ervaren (op een schaal van 1-5)?

- 1 Heel slecht
- 2
- 3
- 4
- 5 Excellent

6 Kent u de artiest persoonlijk?

- Ja
- Nee

7 Voor het concert, in welke mate was u fan van de artiest (op een schaal van 1-5)?

- 1 Ik was niet bekend met (de muziek van) de artiest
- 2
- 3
- 4
- 5 Ik ben een groot fan

8 Hoe lang heeft u het concert bekeken (in minuten)?

*Als u het gehele concert heeft gezien kunt u 'geheel' invullen*

9 Wat was het niveau van uw concentratie tijdens het kijken van het concert (op een schaal van 1-5)?

- 1 Alleen achtergrond
- 2
- 3
- 4
- 5 Totale concentratie

10 Hoe vaak bent uw weggelopen tijdens het concert?

11 Had u problemen met uw (internet)connectie tijdens het concert? Zo ja, leg aub uit. Zo nee, kunt u gewoon 'nee' antwoorden.

12 Waren er anderen in dezelfde ruimte aanwezig die met u meeluisterden/keken?

- Ja
- Nee *(redirected to question 14)*

13 Hoeveel mensen?

Section 3

14 In welke mate voelde u zich verbonden met de ARTIEST tijdens het concert (op een schaal van 1-5)?

- 1 Totaal niet
- 2
- 3
- 4
- 5 Heel erg

15 Kijkend naar het figuur hieronder, geef aan welke van deze cirkels U (self) en de ARTIEST (other) het beste omschrijft tijdens de concertervaring?


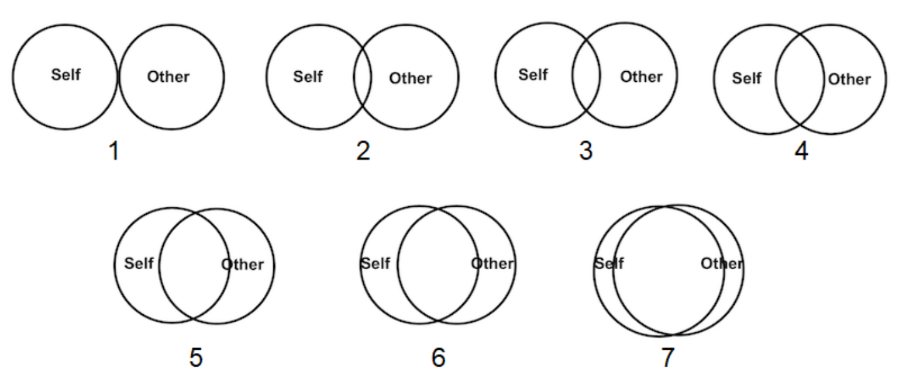


*Bij twijfel, antwoord met uw onderbuikgevoel.*

- 1
- 2
- 3
- 4
- 5
- 6
- 7

16 In welke mate was u zich bewust van andere mensen in het (virtuele) publiek (op een schaal van 1-5)?

*Met het virtuele publiek bedoelen wij de mensen die online naar de livestream kijken*

- 1 Totaal niet
- 2
- 3
- 4
- 5 Heel erg

17 In welke mate had u het gevoel dat u emoties deelde met andere mensen in het (virtuele) publiek (op een schaal van 1-5)?

- 1 Totaal niet
- 2
- 3
- 4
- 5 Heel erg

18 In welke mate had u het gevoel de ervaring te delen met andere mensen in het (virtuele) publiek?

- 1 Totaal niet
- 2
- 3
- 4
- 5 Heel erg

19 Hoe verbonden voelde u zich met het (virtuele) PUBLIEK tijdens het concert (op een schaal van 1-5)?

- 1 Totaal niet
- 2
- 3
- 4
- 5 Heel erg

20 Kijkend naar het figuur hieronder, geef aan welke van deze cirkels U (self) en het (virtuele) PUBLIEK (other) het beste omschrijft tijdens de concertervaring?


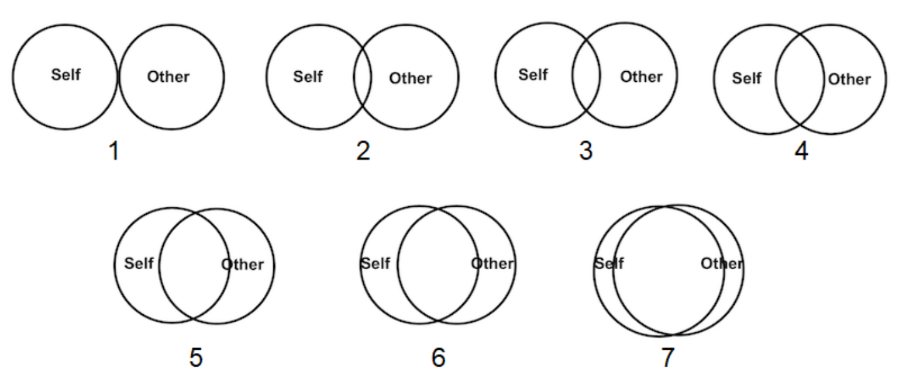


*Bij twijfel, antwoord met uw onderbuikgevoel.*

- 1
- 2
- 3
- 4
- 5
- 6
- 7

21 Kende je andere mensen in het (virtuele) publiek?

- Ja
- Nee / niet dat ik weet

Section 4

22 Gevoelens van 'agency' kunnen beschreven worden als het voelen van subjectieve controle. In andere woorden, 'agency' is een algemeen gevoel van controle over wat je aan het doen bent of de situatie waarin je je bevindt.

Met deze definitie in gedachten, in hoeverre had je een gevoel van 'agency' over jouw concert ervaring (op een schaal van 1-5)?

- 1 Totaal niet
- 2
- 3
- 4
- 5 Heel erg

23 Wanneer twee of meer mensen samen iets ondernemen, kunnen zij gedeelde agency voelen over wat zij aan het doen zijn of de situatie waarin zij zich bevinden. Wanneer je gedeelde agency voelt, voelt het alsof je iets gezamenlijk doet (je ervaring is het product van wat jullie samen doen). Als je geen gedeelde agency voelt, voelt het alsof anderen geen controle hebben over jouw ervaring, en voelt het alsof je totaal onafhankelijk van elkaar handelt.

Met deze definitie in gedachten, in hoeverre had je een gevoel dat er sprake was van gedeelde agency over jouw concert ervaring (op een schaal van 1-5)?

- 1 Totaal niet
- 2
- 3
- 4
- 5 Heel erg

24 Mocht u stemmen op een laatste muziekstuk?

- Ja *(redirected to question 26)*
- Nee

25 Als u de mogelijkheid zou hebben gehad om te stemmen, welk muziekstuk had u gekozen?

- Het melodische stuk: Danza de la moza donosa
- Het ritmische stuk: Danza del gaucho matrero
- Geen idee / totaal geen voorkeur

26 Kende u één of beide stukken al waarop gestemd werd? (meerdere antwoorden mogelijk)

- Ja, ik kende Danza de la moza donosa al
- Ja, ik kende Danza del gaucho matrero al
- Nee

27 Beviel het laatste stuk dat door de artiest werd gespeeld je (op een schaal van 1-5)?

- 1 Totaal niet
- 2
- 3
- 4
- 5 Het was fantastisch

28 Heeft het feit dat u wel / niet was toegestaan om te stemmen uw concertervaring beïnvloed?

- Ja, ik denk dat het mijn ervaring heeft verbeterd
- Ja, ik denk dat het mijn ervaring heeft verslechterd
- Nee, ik denk dat het geen invloed had
- Ik weet het niet

Section 5

29 Tot slot hebben wij een aantal stellingen waarvan wij willen weten of u het daarmee eens of oneens bent.

1 Totaal niet 2 3 4 5 Geheel

Tijdens het livestream concert had ik het gevoel alsof ik "aanwezig was" met de artiest en het publiek.

Ik was totaal in de ban van het concert.

Ik had het gevoel dat ik in de aanwezigheid was van anderen in het livestream concert.

Ik had het gevoel dat anderen zich bewust waren van mijn aanwezigheid.

De mensen in het livestream concert leken mij geëngageerd en actief.

Mijn ervaring in het livestream concert leken op mijn ervaringen tijdens een echt concert.

Tijdens het livestream concert waren er momenten waarop de computerinterface leek te verdwijnen, en ik het gevoel had daadwerkelijk bij het concert aanwezig te zijn.

Tijdens het livestream concert had ik het gevoel betrokken te zijn bij het concert.

Deze manier van een concert bezoeken voldeed aan mijn verwachtingen.

Deze manier van een concert bezoeken gaf mij dezelfde voldoening als fysiek bij een concert aanwezig zijn (zoals voor de pandemie).

Het concert deed mij (kortstondig) mijn zorgen rondom covid19 vergeten.

Ik vergat compleet dat ik meedeed aan onderzoek.

30 Geef aan in hoeverre je de volgende items meer of minder voelde na het bijwonen van het concert:

Veel minder Minder Onveranderd Meer Veel meer

Eenzaamheid

Gebrek aan gezelschap

Geïsoleerd van anderen

Angstig

Section 6

Dank u wel!

Heel erg bedankt voor het meedoen aan dit experiment.

Als u nog vragen of opmerkingen heeft, of op de hoogte zou willen blijven over de resultaten van de studie, laat ze hieronder achter of kom in contact via ipem.sessions@gmail.com

Vergeet niet om op verzenden te drukken!

31 Opmerkingen (optioneel)

## Concert 2

*Concertervaring - Nemø*

Geef antwoord op de volgende vragen over uw ervaringen van het concert.

Section 1

1 Wat is uw persoonlijke ID-code?

Section 2

2 Op welk type scherm heeft u de livestream bekeken?

- Telefoonscherm *(redirected to question 4)*
- VR headset met telefoon
- Tablet *(redirected to question 4)*
- Laptop *(redirected to question 4)*
- Desktop *(redirected to question 4)*
- (Grote) televisie *(redirected to question 4)*
- Grote projectie *(redirected to question 4)*
- Other… *(redirected to question 4)*

3 Heb je ooit eerder een VR headset uitgeprobeerd? (meerdere antwoorden mogelijk)

- Ja, in mijn eigen tijd
- Ja, ik heb de filmpjes uit de instructies uitgeprobeerd
- Nee, dit was de eerste keer

4 Met welk type audio heeft u het concert beluisterd?

- Simpele koptelefoon/oordopjes
- Hoge kwaliteits koptelefoon/oordopjes
- Ingebouwde speakers
- Externe speakers
- Hoge kwaliteit speakers
- Other…

5 Hoe heeft u de videokwaliteit ervaren (op een schaal van 1-5)?

- 1 Heel slecht
- 2
- 3
- 4
- 5 Excellent

6 Hoe heeft u de geluidskwaliteit ervaren (op een schaal van 1-5)?

- 1 Heel slecht
- 2
- 3
- 4
- 5 Excellent

7 Kent u de artiest persoonlijk?

- Ja
- Nee

8 Voor het concert, in welke mate was u fan van de artiesten (op een schaal van 1-5)?

- 1 Ik was niet bekend met (de muziek van) de artiest
- 2
- 3
- 4
- 5 Ik ben een groot fan

9 Hoe lang heeft u het concert bekeken (in minuten)?

*Als u het gehele concert heeft gezien kunt u 'geheel' invullen*

10 Wat was het niveau van uw concentratie tijdens het kijken van het concert (op een schaal van 1-5)?

- 1 Alleen achtergrond
- 2
- 3
- 4
- 5 Totale concentratie

11 Hoe vaak bent uw weggelopen tijdens het concert?

12 Had u problemen met uw (internet)connectie tijdens het concert? Zo ja, leg aub uit. Zo nee, kunt u gewoon 'nee' antwoorden.

13 Waren er anderen in dezelfde ruimte aanwezig die met u meeluisterden/keken?

- Ja
- Nee *(redirected to question 15)*

14 Hoeveel mensen?

15 Beviel de muziek die door de artiesten werd gespeeld je (op een schaal van 1-5)?

- 1 Totaal niet
- 2
- 3
- 4
- 5 Het was fantastisch

Section 3

16 In welke mate voelde u zich verbonden met de ARTIESTEN tijdens het concert (op een schaal van 1-5)?

- 1 Totaal niet
- 2
- 3
- 4
- 5 Heel erg

17 Kijkend naar het figuur hieronder, geef aan welke van deze cirkels U (self) en de ARTIESTEN (other) het beste omschrijft tijdens de concertervaring?


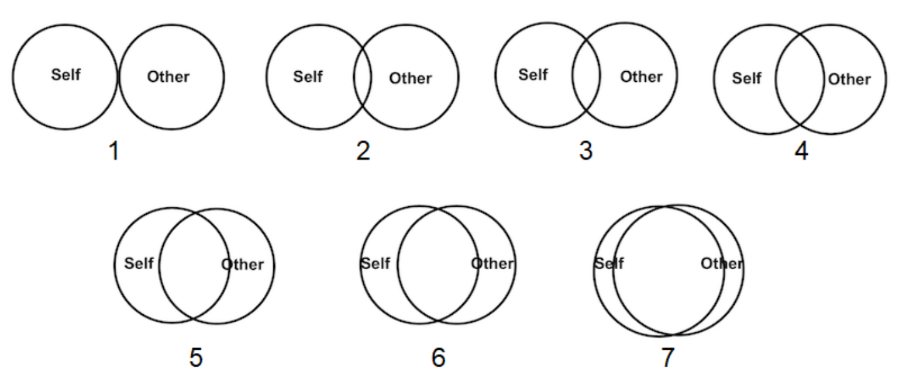


*Bij twijfel, antwoord met uw onderbuikgevoel.*

- 1
- 2
- 3
- 4
- 5
- 6
- 7

18 In welke mate was u zich bewust van andere mensen in het (virtuele) publiek (op een schaal van 1-5)?

*Met het virtuele publiek bedoelen wij de mensen die online naar de livestream kijken*

- 1 Totaal niet
- 2
- 3
- 4
- 5 Heel erg

19 In welke mate had u het gevoel dat u emoties deelde met andere mensen in het (virtuele) publiek (op een schaal van 1-5)?

- 1 Totaal niet
- 2
- 3
- 4
- 5 Heel erg

20 In welke mate had u het gevoel de ervaring te delen met andere mensen in het (virtuele) publiek?

- 1 Totaal niet
- 2
- 3
- 4
- 5 Heel erg

21 Hoe verbonden voelde u zich met het (virtuele) PUBLIEK tijdens het concert (op een schaal van 1-5)?

- 1 Totaal niet
- 2
- 3
- 4
- 5 Heel erg

22 Kijkend naar het figuur hieronder, geef aan welke van deze cirkels U (self) en het (virtuele) PUBLIEK (other) het beste omschrijft tijdens de concertervaring?


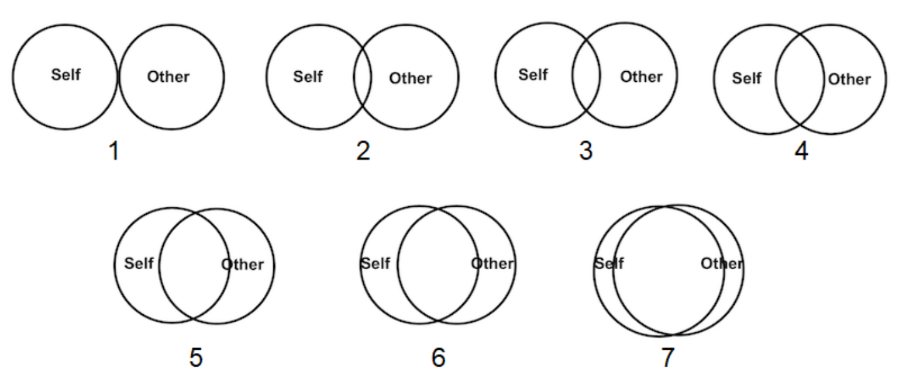


*Bij twijfel, antwoord met uw onderbuikgevoel.*

- 1
- 2
- 3
- 4
- 5
- 6
- 7

23 Kende je andere mensen in het (virtuele) publiek?

- Ja
- Nee / niet dat ik weet

Section 4

24 Geef aub aan in hoeverre de volgende stellingen van toepassing waren op jouw ervaring van het concert:

*Met de virtuele wereld bedoelen we het online (virtuele) concert.*

1 Helemaal mee oneens 2 3 4 5 6 7 Helemaal mee eens

Ik had het gevoel omgeven te zijn door de virtuele wereld.

Ik had het gevoel slechts plaatjes te aanschouwen.

Ik had niet het gevoel in de virtuele ruimte aanwezig te zijn.

Ik had meer het gevoel bezig te zijn in de virtuele ruimte, dan dat ik het gevoel had iets van buitenaf te bedienen.

Ik voelde me aanwezig in de virtuele ruimte.

Ik was me niet bewust van mijn echte omgeving.

Ik lette nog op de echte omgeving.

Ik ging volledig op in de virtuele wereld.

De virtuele wereld kwam echter op mij over dan de werkelijke wereld.

25 Ik had het gevoel aanwezig te zijn in de computerwereld.

1 Helemaal niet 2 3 4 5 6 7 Heel erg

26 Hoe bewust was u zich van de echte omgeving (bv. geluiden van buiten, kamertemperatuur), terwijl u zich bevond in de virtuele ruimte?

1 Zeer bewust 2 3 4 5 6 7 Helemaal niet bewust

27 Hoe echt kwam de virtuele omgeving op u over?

1 Heel echt 2 3 4 5 6 7 Helemaal niet echt

28 In hoeverre kwam uw ervaring in de virtuele omgeving overeen met uw ervaringen in de echte wereld?

1 Geen overeenstemming 2 3 4 5 6 7 Volledige overeenstemming

29 Hoe werkelijk kwam de virtuele wereld op u over?

1 Zoals een denkbeeldige wereld 2 3 4 5 6 7 Niet te onderscheiden van de echte wereld

Section 5

30 Gevoelens van 'agency' kunnen beschreven worden als het voelen van subjectieve controle. In andere woorden, 'agency' is een algemeen gevoel van controle over wat je aan het doen bent of de situatie waarin je je bevindt.

Met deze definitie in gedachten, in hoeverre had je een gevoel van 'agency' over jouw concert ervaring (op een schaal van 1-5)?

- 1 Totaal niet
- 2
- 3
- 4
- 5 Heel erg

31 Wanneer twee of meer mensen samen iets ondernemen, kunnen zij gedeelde agency voelen over wat zij aan het doen zijn of de situatie waarin zij zich bevinden. Wanneer je gedeelde agency voelt, voelt het alsof je iets gezamenlijk doet (je ervaring is het product van wat jullie samen doen). Als je geen gedeelde agency voelt, voelt het alsof anderen geen controle hebben over jouw ervaring, en voelt het alsof je totaal onafhankelijk van elkaar handelt.

Met deze definitie in gedachten, in hoeverre had je een gevoel dat er sprake was van gedeelde agency over jouw concert ervaring (op een schaal van 1-5)?

- 1 Totaal niet
- 2
- 3
- 4
- 5 Heel erg

Section 6

32 Tot slot hebben wij een aantal stellingen waarvan wij willen weten of u het daarmee eens of oneens bent.

1 Totaal niet 2 3 4 5 Geheel

Tijdens het livestream concert had ik het gevoel alsof ik "aanwezig was" met de artiest en het publiek.

Ik was totaal in de ban van het concert.

Ik had het gevoel dat ik in de aanwezigheid was van anderen in het livestream concert.

Ik had het gevoel dat anderen zich bewust waren van mijn aanwezigheid.

De mensen in het livestream concert leken mij geëngageerd en actief.

Mijn ervaring in het livestream concert leken op mijn ervaringen tijdens een echt concert.

Tijdens het livestream concert waren er momenten waarop de computerinterface leek te verdwijnen, en ik het gevoel had daadwerkelijk bij het concert aanwezig te zijn.

Tijdens het livestream concert had ik het gevoel betrokken te zijn bij het concert.

Deze manier van een concert bezoeken voldeed aan mijn verwachtingen.

Deze manier van een concert bezoeken gaf mij dezelfde voldoening als fysiek bij een concert aanwezig zijn (zoals voor de pandemie).

Het concert deed mij (kortstondig) mijn zorgen rondom covid19 vergeten.

Ik vergat compleet dat ik meedeed aan onderzoek.

33 Geef aan in hoeverre je de volgende items meer of minder voelde na het bijwonen van het concert:

Veel minder Minder Onveranderd Meer Veel meer

Eenzaamheid

Gebrek aan gezelschap

Geïsoleerd van anderen

Angstig

Section 7

Dank u wel!

Heel erg bedankt voor het meedoen aan dit experiment.

Als u nog vragen of opmerkingen heeft, of op de hoogte zou willen blijven over de resultaten van de studie, laat ze hieronder achter of kom in contact via ipem.sessions@gmail.com

Vergeet niet om op verzenden te drukken!

34 Opmerkingen (optioneel)

## Concert 3

*Concertervaring - Shalan Alhamwy & Jonas De Rave*

Geef antwoord op de volgende vragen over uw ervaringen van het concert.

Section 1

1 Wat is uw persoonlijke ID-code?

Section 2

2 Hoe heeft u het concert bijgewoond?

- Fysiek aanwezig *(redirected to question 7)*
- Virtueel aanwezig

3 Had u problemen met uw (internet)connectie tijdens het concert? Zo ja, leg aub uit. Zo nee, kunt u gewoon 'nee' antwoorden.

4 Heeft u de chat functie of andere manieren van communicatie (bijv. reacties) gebruikt om uw ervaring te communiceren?

- Ja
- Nee

5 Waren er anderen in dezelfde ruimte aanwezig die met u meeluisterden/keken?

- Ja
- Nee *(redirected to question 8)*

6 Hoeveel mensen?

7 U was fysiek aanwezig bij:

- Het in groep naar de livestream kijken op groot scherm *(redirected to question 10)*
- Het in groep aanwezig zijn bij het concert zelf *(redirected to question 11)*

8 Op welk type scherm heeft u de livestream bekeken?

- Telefoonscherm
- Tablet
- Laptop
- Desktop
- (Grote) televisie
- Grote projectie
- VR headset
- Other…

9 Met welk type audio heeft u het concert beluisterd?

- Simpele koptelefoon/oordopjes
- Hoge kwaliteits koptelefoon/oordopjes
- Ingebouwde speakers
- Externe speakers
- Hoge kwaliteit speakers
- Other…

10 Hoe heeft u de videokwaliteit ervaren (op een schaal van 1-5)?

- 1 Heel slecht
- 2
- 3
- 4
- 5 Excellent

11 Hoe heeft u de geluidskwaliteit ervaren (op een schaal van 1-5)?

- 1 Heel slecht
- 2
- 3
- 4
- 5 Excellent

12 Kent u (één van) de artiest(en) persoonlijk?

- Ja
- Nee

13 Voor het concert, in welke mate was u fan van de artiest(en) (op een schaal van 1-5)?

- 1 Ik was niet bekend met (de muziek van) de artiest
- 2
- 3
- 4
- 5 Ik ben een groot fan

14 Hoe lang heeft u het concert bekeken (in minuten)?

*Als u het gehele concert heeft gezien kunt u 'geheel' invullen*

15 Wat was het niveau van uw concentratie tijdens het kijken van het concert (op een schaal van 1-5)?

- 1 Alleen achtergrond
- 2
- 3
- 4
- 5 Totale concentratie

16 Hoe vaak bent uw weggelopen tijdens het concert?

17 Beviel de muziek die door de artiesten werd gespeeld je (op een schaal van 1-5)?

- 1 Totaal niet
- 2
- 3
- 4
- 5 Het was fantastisch

Section 3

18 In welke mate voelde u zich verbonden met de ARTIESTEN tijdens het concert (op een schaal van 1-5)?

- 1 Totaal niet
- 2
- 3
- 4
- 5 Heel erg

19 Kijkend naar het figuur hieronder, geef aan welke van deze cirkels U (self) en de ARTIESTEN (other) het beste omschrijft tijdens de concertervaring?


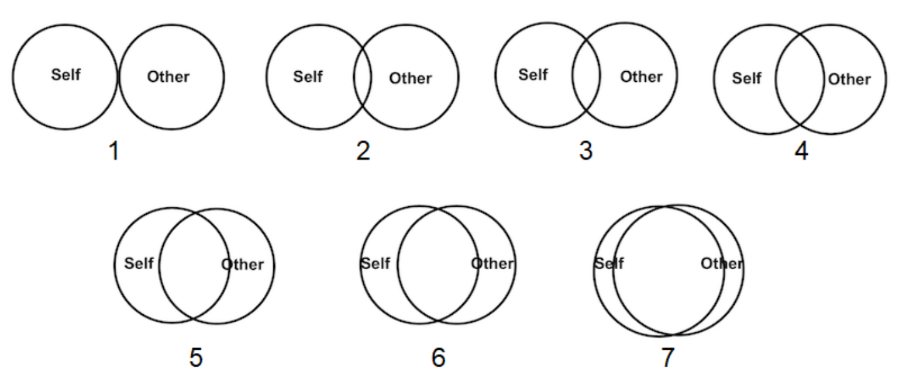


*Bij twijfel, antwoord met uw onderbuikgevoel.*

- 1
- 2
- 3
- 4
- 5
- 6
- 7

20 In welke mate was u zich bewust van andere mensen in het publiek (op een schaal van 1-5)?

- 1 Totaal niet
- 2
- 3
- 4
- 5 Heel erg

21 In welke mate had u het gevoel dat u emoties deelde met andere mensen in het publiek (op een schaal van 1-5)?

- 1 Totaal niet
- 2
- 3
- 4
- 5 Heel erg

22 In welke mate had u het gevoel de ervaring te delen met andere mensen in het publiek?

- 1 Totaal niet
- 2
- 3
- 4
- 5 Heel erg

23 Hoe verbonden voelde u zich met het PUBLIEK tijdens het concert (op een schaal van 1-5)?

- 1 Totaal niet
- 2
- 3
- 4
- 5 Heel erg

24 Kijkend naar het figuur hieronder, geef aan welke van deze cirkels U (self) en het PUBLIEK (other) het beste omschrijft tijdens de concertervaring?


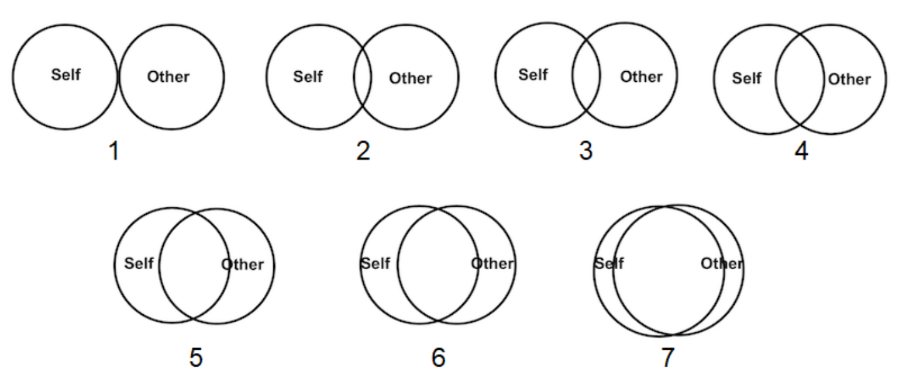


*Bij twijfel, antwoord met uw onderbuikgevoel.*

- 1
- 2
- 3
- 4
- 5
- 6
- 7

25 Kende je andere mensen in het publiek?

- Ja, in het fysiek aanwezige publiek
- Ja, in het virtueel aanwezige publiek
- Ja, in zowel het fysiek als virtueel aanwezige publiek
- Nee / niet dat ik weet

Section 4

26 Gevoelens van 'agency' kunnen beschreven worden als het voelen van subjectieve controle. In andere woorden, 'agency' is een algemeen gevoel van controle over wat je aan het doen bent of de situatie waarin je je bevindt.

Met deze definitie in gedachten, in hoeverre had je een gevoel van 'agency' over jouw concert ervaring (op een schaal van 1-5)?

- 1 Totaal niet
- 2
- 3
- 4
- 5 Heel erg

27 Wanneer twee of meer mensen samen iets ondernemen, kunnen zij gedeelde agency voelen over wat zij aan het doen zijn of de situatie waarin zij zich bevinden. Wanneer je gedeelde agency voelt, voelt het alsof je iets gezamenlijk doet (je ervaring is het product van wat jullie samen doen). Als je geen gedeelde agency voelt, voelt het alsof anderen geen controle hebben over jouw ervaring, en voelt het alsof je totaal onafhankelijk van elkaar handelt.

Met deze definitie in gedachten, in hoeverre had je een gevoel dat er sprake was van gedeelde agency over jouw concert ervaring (op een schaal van 1-5)?

- 1 Totaal niet
- 2
- 3
- 4
- 5 Heel erg

Section 5

28 Tot slot hebben wij een aantal stellingen waarvan wij willen weten of u het daarmee eens of oneens bent.

1 Totaal niet 2 3 4 5 Geheel

Tijdens het livestream concert had ik het gevoel alsof ik "aanwezig was" met de artiest en het publiek.

Ik was totaal in de ban van het concert.

Ik had het gevoel dat ik in de aanwezigheid was van anderen in het livestream concert.

Ik had het gevoel dat anderen zich bewust waren van mijn aanwezigheid.

De mensen in het livestream concert leken mij geëngageerd en actief.

Mijn ervaring in het livestream concert leken op mijn ervaringen tijdens een echt concert.

Tijdens het livestream concert waren er momenten waarop de computerinterface leek te verdwijnen, en ik het gevoel had daadwerkelijk bij het concert aanwezig te zijn.

Tijdens het livestream concert had ik het gevoel betrokken te zijn bij het concert.

Deze manier van een concert bezoeken voldeed aan mijn verwachtingen.

Deze manier van een concert bezoeken gaf mij dezelfde voldoening als fysiek bij een concert aanwezig zijn (zoals voor de pandemie).

Het concert deed mij (kortstondig) mijn zorgen rondom covid19 vergeten.

Ik vergat compleet dat ik meedeed aan onderzoek.

29 Geef aan in hoeverre je de volgende items meer of minder voelde na het bijwonen van het concert:

Veel minder Minder Onveranderd Meer Veel meer

Eenzaamheid

Gebrek aan gezelschap

Geïsoleerd van anderen

Angstig

Section 6

Dank u wel!

Heel erg bedankt voor het meedoen aan dit experiment.

Als u nog vragen of opmerkingen heeft, of op de hoogte zou willen blijven over de resultaten van de studie, laat ze hieronder achter of kom in contact via ipem.sessions@gmail.com

Vergeet niet om op verzenden te drukken!

30 Opmerkingen (optioneel)

# Supplementary Material S5: Multimodal Presence Scale

The Multimodal Presence Scale was designed and developed by Makransky et al., 2017. In the Corona Concerts project (Swarbrick et al., 2021), relevant items were re-worded to increase their relevance for the virtual concert experience as recommended by the MPS authors (Makransky et al., 2017). Specifically, items from the physical and social presence dimensions were used (see S3 and S4 for the questions).

To evaluate if the structure of our adjusted presence scale was the same as the multimodal presence scale, we conducted a principal component analysis using the principal function from the psych toolbox (Revelle, 2020). It is theoretically likely that social presence and physical presence items would correlate, therefore we conducted an oblimin rotation to facilitate the interpretation of the component loadings. As expected, the principal component analysis revealed that there were two principal components with eigenvalues greater than 1 (component 1 = 3.477, component 2 = 1.216). We then proceeded by rotating the two retained components using oblimin rotation. The cumulative variance explained was 67% (component 1 = 36% and component 2 = 31%). We employed a cut-off threshold of +/-0.4 to determine which items loaded onto each factor. The items that loaded onto the first transformed component were #1. “My experience in the streamed concert seemed similar to my experiences in a real concert.” (0.75), #2. “While I was at the streamed concert, I had a sense of ‘being there’ with the performers and audience members.” (0.73), #3. "I was completely captivated by the streamed concert." (0.86), and #4. "During the streamed concert there were times where the computer interface seemed to disappear, and I felt like I was actually at the concert with the audience and performers" (0.69). The items that loaded onto the second transformed component were #5. "I felt like I was in the presence of other people who were online in the streamed concert." (0.70), #6. "I felt that the other people in the streamed concert were aware of my presence." (0.83), and #7. "The people in the streamed concert appeared to be engaged and active to me." (0.89). Based on the results of this analysis, the first component was interpreted as the physical presence subscale and the second component was interpreted as the social presence subscale. These subscales were averaged to provide separate measures of social and physical presence. These results are slightly different than the original MPS because item #4 originally loaded onto the social presence subscale but our re-wording made it load onto the physical presence subscale.

The item that changed between the subscales was originally worded “During the simulation there were times where the computer interface seemed to disappear, and I felt like I was working directly with another person.” by Makransky et al. (2017) and it was adjusted to “During the streamed concert there were times where the computer interface seemed to disappear, and I felt like I was actually at the concert with the audience and performers” by Swarbrick et al. (2021) and this new wording resulted it in loading onto the physical presence subscale.

# Supplementary Material S6: Descriptive Statistics Concert 3

Table S5: The mean and standard deviation of each group in concert 3. The physically present group was excluded from the analyses because only 6 participants were in this group. Full data on the physically present group and all data collected during these concerts is located on the OSF repository (osf.io/D3Z8E).

| Predictor | Physically present | | Zoom | | YouTube | |
| --- | --- | --- | --- | --- | --- | --- |
|  | ***Mean*** | ***(SD)*** | ***Mean*** | ***(SD)*** | ***Mean*** | ***(SD)*** |
| *Audio quality* | 4.7 | (0.8) | 3.7 | (1.1) | 4.1 | (1) |
| *Concentration* | 4.5 | (0.5) | 3.5 | (0.8) | 3.7 | (1.2) |
| *Self-agency* | 3.7 | (0.5) | 2.9 | (1) | 3.6 | (0.9) |
| *Shared agency* | 3 | (0.9) | 3.1 | (1.1) | 2.4 | (1.6) |
| *Social presence* | 2.7 | (0.7) | 3.3 | (0.9) | 2.4 | (0.9) |
| *Physical presence* | 3.5 | (0.5) | 2.0 | (0.7) | 2.3 | (1) |
| *Social connection (artist)* | 4 | (0.6) | 2.4 | (0.7) | 3.1 | (0.9) |
| *Inclusion other in self (artist)* | 4 | (0.6) | 2.3 | (1.3) | 2.9 | (1.5) |
| *Social connection (audience)* | 2.7 | (0.5) | 2.8 | (1.1) | 2 | (1.1) |
| *Inclusion other in self (audience)* | 2. 7 | (0.5) | 3.1 | (1.5) | 2.1 | (1.3) |
| *Shared emotions with audience* | 2. 7 | (1) | 2.5 | (0.9) | 1.7 | (1.1) |
| *Shared experience with audience* | 3. 7 | (1.2) | 2.9 | (1) | 2.1 | (1.2) |

# Supplementary Material S7: Effect Size Estimates

Effect size estimates were calculated using the Wilcoxon effect size estimator provided in the rstatix package (Tomczak and Tomczak, 2014; Kassambara, 2020), however they should be interpreted with caution because a trade-off for using robustness afforded by the non-parametric Wilcoxon test is that the effect size estimates lack meaning.

Concert 2: VR headset vs. YouTube 360° vs. normal YouTube livestream

| Dependent variable | Comparison | Effect size *r* | *P*-value |
| --- | --- | --- | --- |
| Average presence (MPS) | VR headset > normal YouTube | *r* = .60 | *p* = .013 |
| Physical presence (MPS) | VR headset > normal YouTube | *r* = .67 | *p* = .004 |
| Average presence (IPQ) | VR headset > normal YouTube | *r* = .65 | *p* = .006 |
| Average presence (IPQ) | VR headset > YouTube 360° | *r* = .64 | *p* = .007 |
| Spatial Presence (IPQ) | VR headset > normal YouTube | *r* = .66 | *p* = .005 |
| Spatial Presence (IPQ) | Trending difference:  VR headset > YouTube 360° | *r* = .47 | *p* = .054 |
| Spatial Presence (IPQ) | Trending difference:  YouTube 360° > normal YouTube | *r* = .43 | *p* = .054 |
| Involvement (IPQ) | VR headset > normal YouTube | *r* = .58 | *p* = .018 |
| Involvement (IPQ) | VR headset > YouTube 360° | *r* = .53 | *p* = .029 |

Concert 3: Zoom vs. normal YouTube livestream

| Dependent variable | Comparison | Effect size *r* | *P*-value |
| --- | --- | --- | --- |
| Social presence (MPS) | Zoom > normal YouTube | *r* = .51 | *p* = .017 |

# Supplementary Material S8: Influence of Voting on Experience

Table S7: Frequency of participants responses. Participants were asked “Did the fact you were allowed / not allowed to vote influence your concert experience?” with response options:

- Yes, I think it improved my experience
- Yes, I think it worsened my experience
- No, I don't think it was of influence
- I do not know

Allowed Vote: 0 = Did not vote; 1 = Voted;

| Allowed Vote | Influence on Experience | Count |
| --- | --- | --- |
| 0 | No change | 4 |
| 0 | Yes improved | 0 |
| 0 | Yes worsened | 8 |
| 0 | I don't know | 1 |
| 1 | No change | 8 |
| 1 | Yes improved | 6 |
| 1 | Yes worsened | 1 |
| 1 | I don't know | 1 |

# Supplementary Material S9: Audio and Video Quality

Participants were encouraged to watch the concerts on laptops with headphones; however participants reported using a variety of other equipment as well. We also asked participants “How did you experience the video/audio quality? (1 Very bad–5 Excellent)”. To understand how the video and audio device affected quality, here we display the frequency participants reported each quality rating across devices.


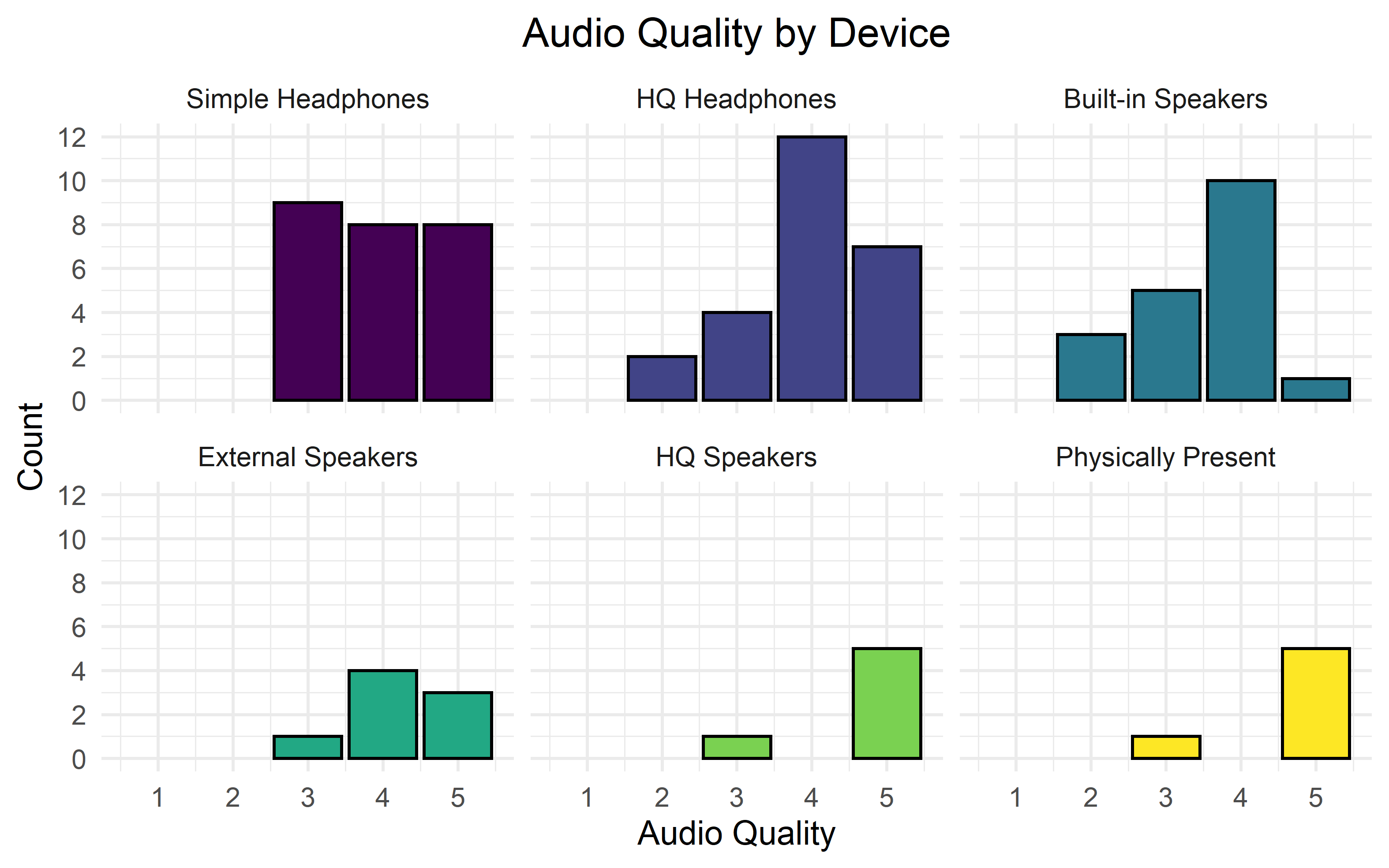

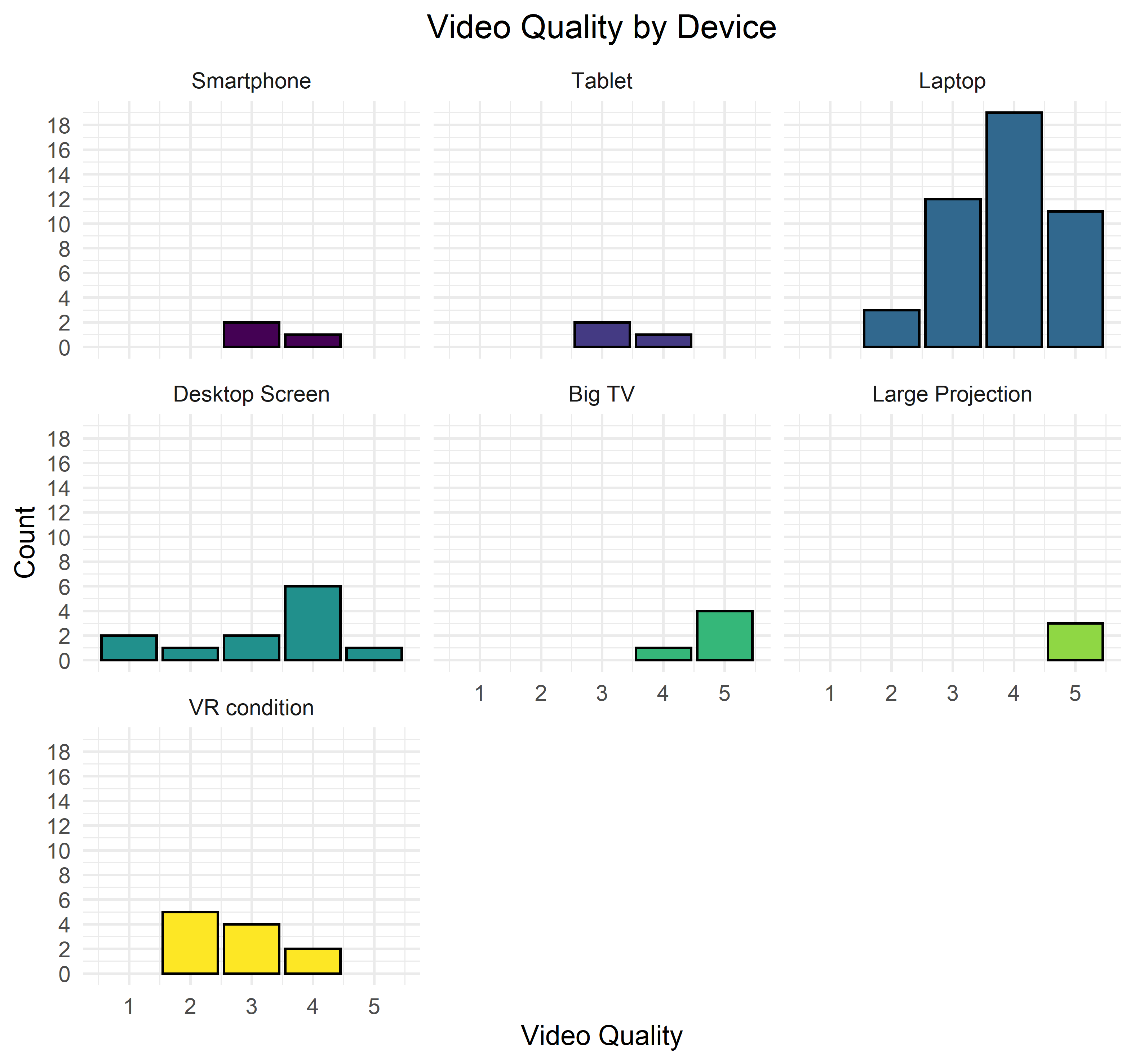


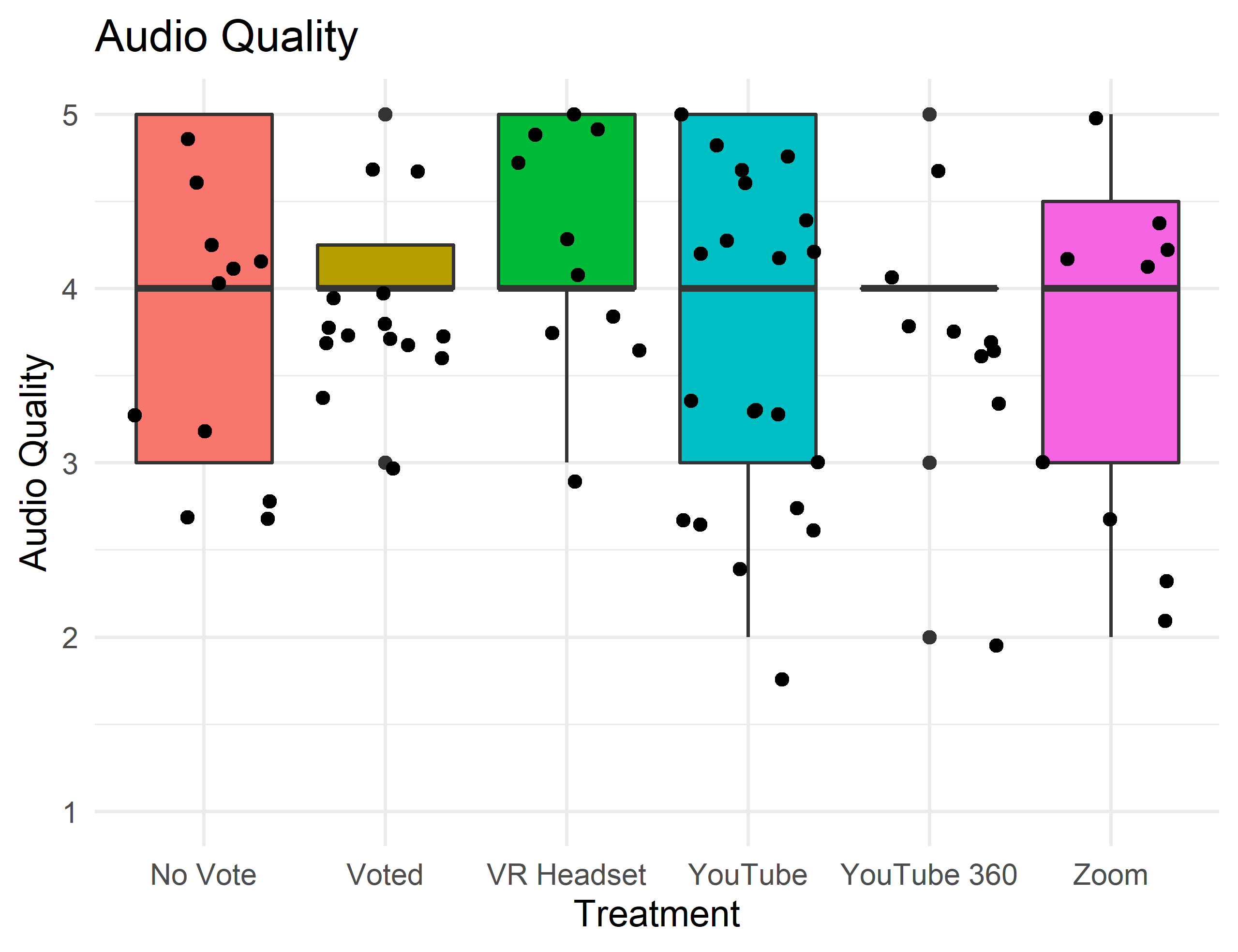


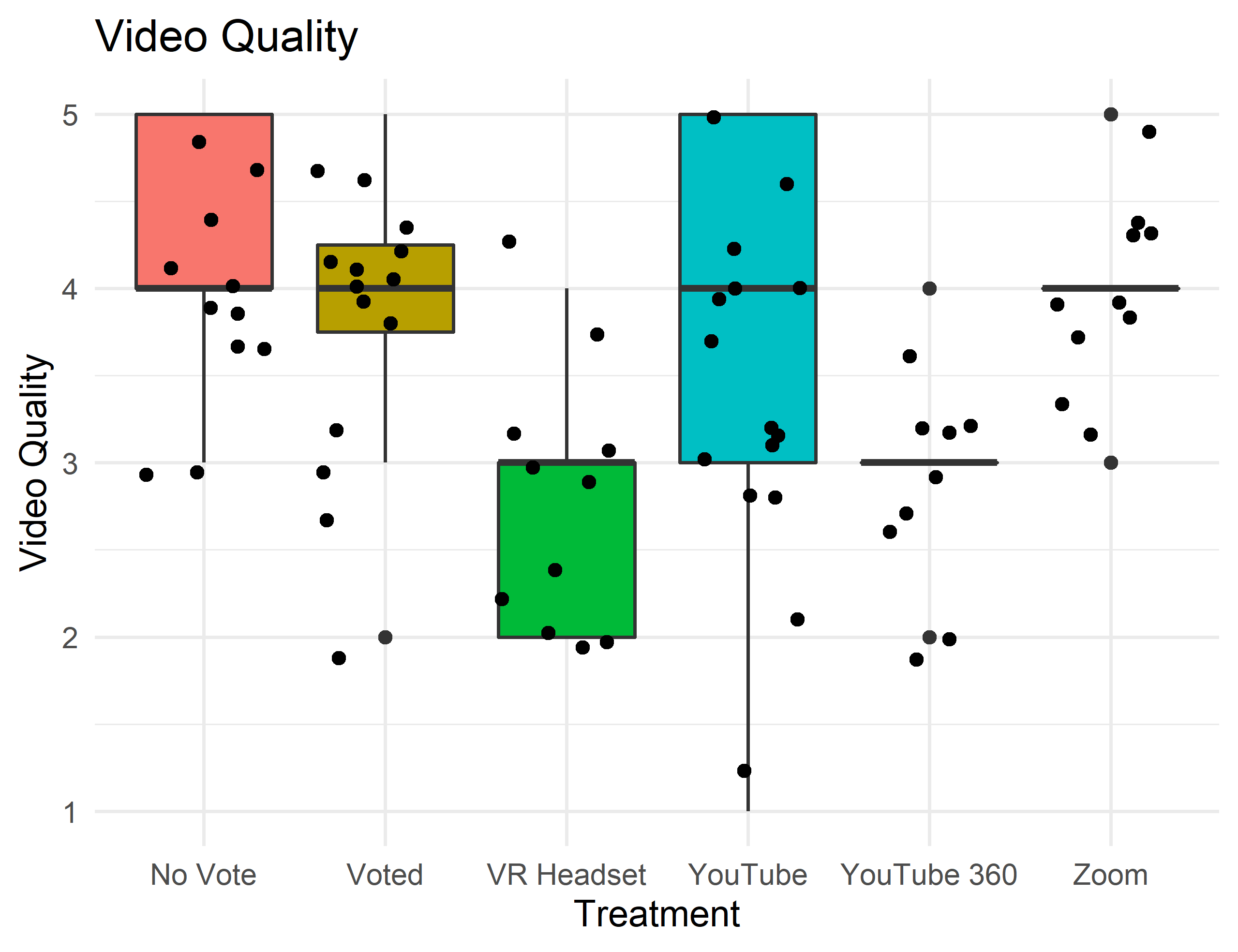

Supplement: Supplementary file 1 [file Data_Sheet_1.docx]
